# Global impacts of drifting fish aggregating devices on marine protected areas

**DOI:** 10.1126/sciadv.aee6998

**Published:** 2026-06-17

**Authors:** Laurenne Schiller, Juan Carlos Villaseñor-Derbez, John Lynham, Boris Worm

**Affiliations:** ^1^ Biology Department, Dalhousie University, Halifax, Canada.; ^2^ Department of Environmental Science and Policy, Rosenstiel School for Marine, Earth, and Atmospheric Science and Frost Institute for Data Science and Computing, University of Miami, Miami, FL, USA.; ^3^ Department of Economics and UHERO, University of Hawaiʻi at Mānoa, Honolulu, HI, USA.

## Abstract

Protected areas are a critical component of efforts to reverse biodiversity loss by 2050. In the ocean, free-drifting fish aggregating devices (dFADs) are released in large numbers by industrial purse seine fishing companies to help catch tuna. These devices can enter marine protected areas (MPAs) undetected, potentially leading to wildlife entanglement, plastic pollution, and habitat degradation. Here we investigate processes by which dFADs may compromise MPA objectives and assess the burden they put on existing MPAs. By analyzing drift, strandings, and expert interview data, we show that dFADs have likely interacted with 53% of the global MPA network by area and stranded in 174 protected areas, which are home to at least 490 at-risk species. While recent improvements to dFAD design should reduce harm to wildlife, our findings suggest that improved regulation, transparency, and industry accountability are required to mitigate additional effects on MPAs, especially around documented hotspots in the central Pacific, western Indian Ocean, and Caribbean.

## Teaser

Free-drifting fish aggregating devices pose complex ecological and social challenges to marine protected areas worldwide.

## INTRODUCTION

Over the past 25 years, human impacts on marine biodiversity have become increasingly pervasive, motivating a rapid increase in targeted conservation efforts, such as protected areas and other effective area-based conservation measures aimed at restoring what has been lost (*1*). Currently, 16,516 marine protected areas (MPAs) cover 8.4% (30.3 million km^2^) of Earth’s ocean surface (*2*), and increasing coverage to 30% by 2030 is a key priority of the Kunming-Montréal Global Biodiversity Framework (*3*). While the specific ecological objectives associated with MPA implementation are largely country- and site-specific, such areas are generally established for the purpose of minimizing or reversing human impacts on marine habitats, protecting endangered wildlife, or rebuilding depleted fish stocks.

To-date, small island nations and overseas territories have been frontrunners in designating large MPAs and exceeding domestic targets (*4*–*6*). Many of these jurisdictions are located in prime fishing grounds for tuna, where the establishment of large, highly protected MPAs may provide spatial refuges for vulnerable life stages (*7*, *8*) and provide “spillover” benefits to regional fisheries (*9*–*11*), outcomes that can complement sustainable fisheries management strategies [e.g. (*12*)]. Still, only 3% of ocean area is currently in “highly protected” areas that exclude commercial fishing vessels (*13*), and no MPAs are protected from abandoned or lost fishing gear, including drifting fish aggregating devices (dFADs), free-drifting rafts used extensively by tuna purse seine fishing companies.

While FADs anchored to the seafloor have been used to improve coastal fisheries catches for millennia (*14*, *15*), the use of free-drifting FADs is much more recent. Popularized by European fleets in the 1990s, dFADs exploit the tendency of tuna to aggregate around floating debris in the open ocean. Traditional designs usually included submerged nets—reaching 50 to 80 m deep—to increase fish aggregation and slow the rate of drift (*16*, *17*). Now outfitted with Global Positioning System (GPS) and echosounder buoys, these devices allow fishing companies to aggregate, track, and target tuna schools across ocean basins. This increase in predictability can create economic benefits to purse seine fleets by reducing search times for fish (*18*) and stabilizing catch rates (*19*), and many small island developing states have a substantial dependence on revenue derived from these fisheries (*20*, *21*). Today, an estimated 100,000 to 150,000 dFADs are deployed annually (*22*, *23*), and one-third of global tuna landings are now caught with the help of these devices. Moreover, since 2018, more than 90 dFAD fishing companies have been recognized as “sustainable” by the world’s leading seafood eco-certification body, the Marine Stewardship Council (MSC) (*23*). At the same time, the MSC acknowledges that, “[t]ackling the problems associated with FAD fishing is critical to our ocean’s health and productivity” (*24*). Such longstanding problems include the overfishing of juvenile tuna, bycatch of sharks and other wildlife, potential changes in fish habitat, and plastic pollution, among others (*19*, *25*–*27*).

**Fig. 1.**
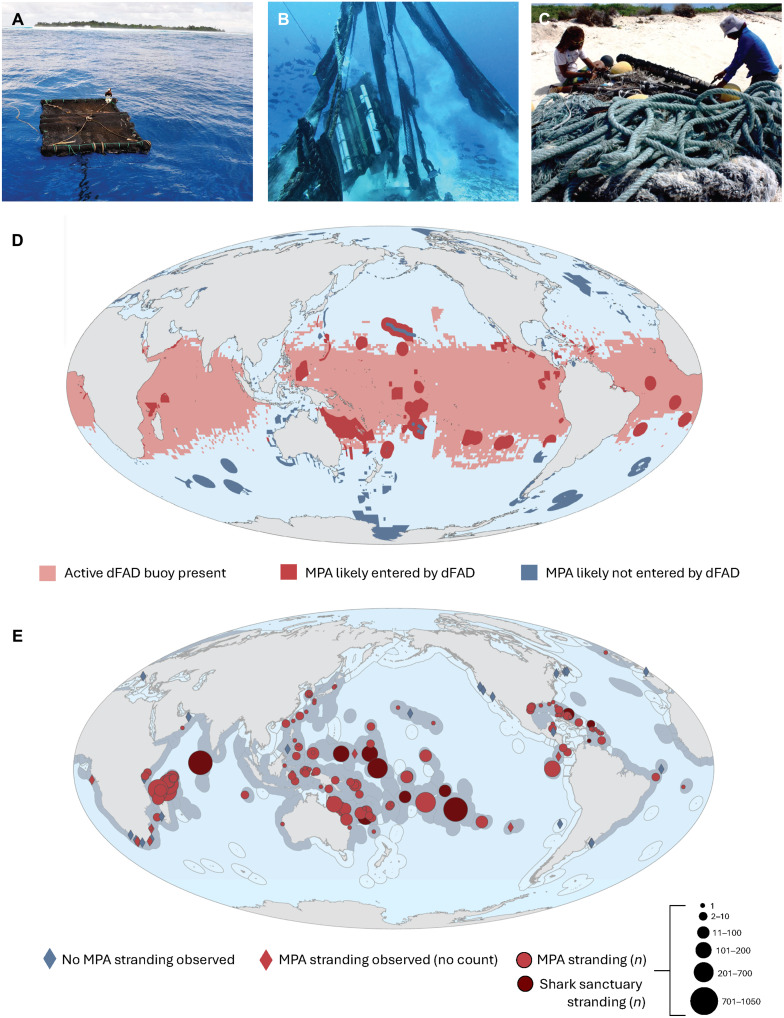
Global impacts of dFADs on MPAs. (**A**) Abandoned dFAD drifting at Palmyra Atoll (no-take MPA); (**B**) sunken dFAD on reef in French Polynesia (shark sanctuary); (**C**) clean-up of stranded dFAD by local environmental organization at Aldabra Atoll in the Seychelles (no-take MPA); (**D**) intersection between 88,359 tracked dFAD buoys (pink) with existing MPAs showing where dFADs have likely entered (red) or not (blue); (**E**) MPAs and shark sanctuaries where dFAD strandings were identified with count (circle), observed but not counted (red diamond), or not observed (blue diamond). Shaded in gray are all maritime jurisdictions that have documented dFAD strandings, independent of their MPA coverage. Photo credits: (A) K. Pollock/Palmyra Program, The Nature Conservancy; (B) L. Clément/Direction des Ressources Marines (French Polynesia); (C) Seychelles Islands Foundation (SIF).

Despite their ubiquity, dFADs are challenging to observe at sea because of their semisubmerged design and low surface profile (Fig. 1A). Fishing companies also retain proprietary control over GPS echosounder buoy data, which makes assessing their global distribution challenging (*19*) as devices are not observable through publicly available satellite data or imagery. Thus, it is currently unknown to what extent dFADs interact with the world’s growing MPA network and how these interactions may affect conservation outcomes (*28*). Despite this shortcoming, several processes whereby a dFAD could interact with a protected area have been identified (see Table 1 for overview and fig. S1 for detailed schematic). Drifting freely and transported by currents, dFADs can enter protected areas undetected (Fig. 1A) and potentially aggregate tuna to be caught by purse seiners once the device exits MPA boundaries (*29*). Alternatively, dFADs may disintegrate, sink, or strand on a shoreline or reef inside the MPA (Fig. 1B), contributing to marine pollution (*30*, *31*) and wildlife entanglement (*32*). Last, stranded dFAD debris can be retrieved by MPA staff, if capacity exists (Fig. 1C) (*33*).

**Table 1.** dFAD impacts to protected areas based on interviewee perspectives. Impacts are organized relative to different processes during the dFAD life cycle, from deployment to retrieval. The regional presence within each ocean basin (indicated with “x”) was based on interviewee observations for MPAs, and the source of each quote is provided in brackets as the interviewee’s identification number. Note: All protected areas discussed here (*n* = 18) prohibit industrial-scale fishing, and nine are “no-take” (i.e., fully protected from all extraction). See table S4 for additional detail on wildlife impacts associated with stranding and table S5 for detail on retrieval challenges associated with stranding.

Process	Impact to MPA	Impact type	Indian	Atlantic	W. Pacific	E. Pacific	Example of interaction
Deployment	Unauthorized/unregulated entry (intentional)	Social, economic, ecological				x*	“It [deploying at MPA boundaries] occurs. It’s something that the captains do, but they manage the drift with programs, trying not to get it [the dFAD] stranded on an island. You don't want to lose it. They will always try for it to pass through [the MPA] and aggregate some fish and come out. Sometimes it will, sometimes it doesn’t.” (INT-1).
Drifting	Unexpected hazard to local vessels	Social	x			x	“It’s not just a question of local fishers concerned that they [dFADs] are kind of stealing the fish, but it’s also a navigation hazard. A lot of [small-scale] fishing in [MPA] is done in speedboats at night. And if you hit one [a dFAD] coming back at speed…it’s really a public safety issue as well as an ecological issue.” (INT-7).
	Invasive species transport	Ecological			x	x	“One other concern in terms of impacts is the potential for FADs to bring invasive species. There’s a strong biosecurity protocol, [and] if your vessel comes into [MPA], the divers check the hull, and if there’s anything on it, you have to leave, and clean it, and then come back. And that’s not the same with FADs of course.” (INT-9).
Aggregating	Ecological trap†	Ecological	x		x		“We have a lot of migratory seabird species that come and nest around [MPA country], and there has been a strong decline of those populations… At this stage, it’s just an assumption that it could be due to dFADs because the dFADs are going around the ocean and pulling bait with them and that bait is then followed by the birds restricting them from nesting where they would normally nest.” (INT-14).
Loss or abandonment	Unauthorized entry (accidental)	Ecological	x	x	x	x	“Typically, the model has been—across the world—that the FADs will drift, and they [fishing vessel operators] will keep tracking them until they drift out of the feasible area where vessels will fish. Then, they turn off the satellite buoys because then you don’t have the cost of transmission. But we don’t know where they end up after that.” (INT-3).
Entanglement	Injury or mortality to wildlife	Ecological	x	x	x	x	“I have personally seen hawksbill turtles, green turtles, loggerhead turtles, olive ridley turtles, and several shark species dead or entangled within dFADs.” (INT-14).
Disintegration	Pollution in protected area	Social	x	x	x	x	“In that clean-up at [MPA], I can't remember how many FADs it was [that were found], but it was a lot of whole FADs. But I think what’s sometimes not talked about is the broken-down FADs as well. And the main issue with those is the wooden frames. Obviously, wood is not an issue like plastic is, but the amount of it on the beaches was insane. The whole beach was just covered in bamboo pieces from the FADs.” (INT-15).
	Ingestion of plastics by wildlife	Ecological	x			x	“Even if you find a FAD and there’s no species entangled, you definitely have concerns about what it could do next. On the coastlines, [there is] very high UV action, so you get really fast breakdown of plastics… [the impact goes] all the way through the food chain.” (INT-9).
Sinking	Deep-sea habitat degradation	Ecological			x		“A lot of deep-sea corals—which are really different than shallow tropical corals—are super long-lived…it’s sparse down there [on the seafloor] in some places so it [a dFAD] could fall and it would be fine. But for any coral you disrupt, you’re talking about at least centuries of growth being disrupted by a single event.” (INT-17).
Stranding	Coastal habitat degradation	Social, economic, ecological	x	x	x	x	“I’ve tried to get them [dFADs] off reefs in a lot of places. It’s like a plow going through a field; they pretty much trash the reef. And it’s not just the footprint of the FAD itself, but obviously the net associated with it… I’ve seen a lot of FADs wash up on reefs, and the damage is pretty irreparable in the short term.” (INT-11).
	Disruption of nesting behaviour	Ecological	x	x			“[The MPA] has a five-meter-wide strip of beach. It’s not very broad, so if there’s something sitting on it, it’s taking away most of the habitat for them [nesting sea turtles]… a 200-pound female might be able to negotiate around it in the dark and find a place, [but] the hatchlings will not be able to climb over that [stranded dFAD]. They emerge at night and try to get to the ocean. If the sun comes up because they’re struggling and they haven’t made it, then they're going to dry out and die, or birds are going to start eating them.” (INT-8).
Retrieval	Financial and logistical burden to MPA staff	Economic, social	x	x	x	x	“There’s a huge cost involved in terms of taking boats out there to [remote MPA], collecting them [dFADs] from the reef, and bringing them all the way back to [main inhabited island]. It’s very time consuming and expensive, and we lack local capacity in terms of vessels and personnel so it’s a huge undertaking.” (INT-13).
	Burden on local waste management system	Economic, social	x	x	x	x	“In an island system like [MPA], where waste management is a huge issue and the landfill is under massive pressure, the [retrieved] FADs add a huge amount of pressure to that because one FAD weighs so much and is so big…that’s just another factor to keep in mind in terms of the burden that it puts on local resources.” (INT-9).

* Only purse seine company representatives could speak to whether their fleets intentionally engage in this practice (see example quote). However, all nine interviewees affiliated with no-take MPAs had observed active dFADs floating in MPA waters, and 54% of MPA interviewees suspected that dFADs were being intentionally deployed at protected area boundaries to aggregate tuna while crossing through. MPA-affiliated interviewees from all regions highlighted that abandoned dFADs drift into protected areas (see “Loss or abandonment”).

† On the basis of local ecological knowledge and observations over time, these interviewees speculated that changes to species behavior and/or condition could be linked to the increased use of dFADs. However, all noted that there was a paucity of data and analyses to empirically confirm the degree to which these changes are due to increasing dFAD use relative to other environmental factors and threats.

Here, we used a mixed-methods approach to derive where and how the global MPA network has been affected by dFADs and determine how to minimize further impacts. We compiled and analyzed all publicly available dFAD drifting, fishing, and stranding data in relation to established MPAs. To fill empirical data gaps, we also deployed a global survey to collect observations from MPA practitioners, and we conducted in-depth interviews with industry and MPA stakeholders to better understand possible dFAD impacts and mechanisms by which they occur, as well as practical solutions to limit their effects.

## RESULTS AND DISCUSSION

### Drift and aggregation

We hypothesized that the widespread distribution and free movement of dFADs makes a large fraction of MPAs vulnerable to undetected entry. By overlaying published data on 88,359 dFAD drift trajectories (*34*–*37*) with the current network of MPAs at a 1°-by-1° scale, we infer that dFADs may have entered 1544 MPAs representing 53% of the global network by area (Fig. 1D). These likely interactions are concentrated between 30°N and 30°S with MPAs in this region accounting for 97% of all MPAs likely affected by dFAD drift. Because of the resolution of the available data, this analysis shows only the likely overlap between dFADs and the current MPA network, and we were unable to determine the impact of dFADs on wildlife in these MPAs while at sea.

**Fig. 2.**
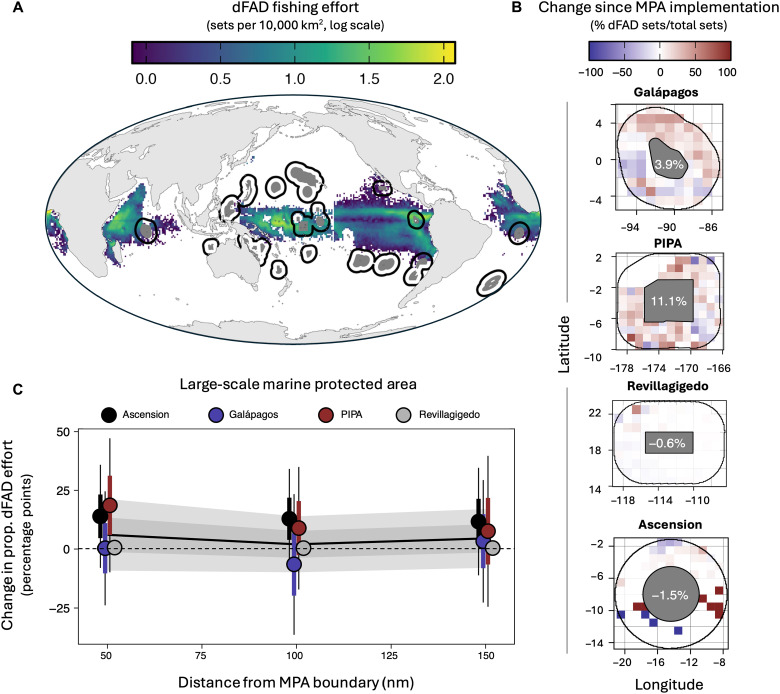
dFAD fishing effort around large-scale MPAs. (**A**) Reported global tuna fishing effort using dFADs (colors; number of sets refer to discrete fishing events where a net is set around a dFAD) and current large-scale MPA network (gray) with a 200-nm buffer shown in black. (**B**) Changes in distribution of reported dFAD fishing effort (proportion of all purse seine sets) in 200-nm surrounding areas since implementation of four highly protected large-scale MPAs. Numbers inside MPAs indicate the temporal change in dFAD effort for all sets around the MPA. (**C**) Mean change in dFAD effort proportional to total effort as a function of distance to MPA boundary since the MPA was implemented (relative to effort 150 to 200 nm away). Black line shows pooled estimates for all MPAs, with dark and light shades representing Newey-West SEs and 95% confidence intervals. Error bars show Newey-West SEs (thick bars) and 95% confidence intervals (thin bars); PIPA, Phoenix Islands protected area.

While most industrial fishing vessels can be tracked and detected if fishing inside an MPA (*38*, *39*), dFADs might allow vessels to extract fish from inside MPAs while remaining outside (fig. S1). We specifically tested this hypothesis by analyzing changes in patterns of reported fishing effort around large (>100,000 km^2^) highly protected MPAs, assuming that targeted dFAD fishing would result in increased dFAD effort near the MPA after it has been established. We identified five MPAs in regions where dFAD purse seine fishing occurs (Fig. 2A), and all but one (the de-gazetted Chagos MPA in the Indian Ocean) had fishing data available from before and after MPA establishment. For two MPAs, the percentage of purse seine fishing events (called sets) using dFADs within 200 nautical miles (nm) of MPA boundaries increased after the MPAs were established: by 4 percentage points around the Galápagos Marine Reserve and by 11 percentage points around the Phoenix Islands protected area (PIPA) (Fig. 2B and fig. S2). In contrast, the percentage of sets fished on dFADs around Revillagigedo and Ascension Island decreased slightly by −0.6 and −1.5 percentage points, respectively. In absolute terms, dFAD fishing effort remained consistently low around Revillagigedo (<10 fished dFAD sets annually) but numbered in the hundreds around the other MPAs, with marked increases in fishing effort around PIPA after MPA establishment but not elsewhere (fig. S3). While areas within 200 nm of MPA boundaries are subject to substantial dFAD fishing, we did not find evidence that dFAD fishing has disproportionately increased in the areas closest to MPA boundaries, relative to changes observed farther away (Fig. 2C, fig. S4, and table S1). This suggests the increases around the Galápagos and Phoenix Islands are in line with region-wide increases in dFAD fishing effort (fig. S5) rather than evidence of targeted dFAD fishing near these MPAs.

When discussing deployment strategies, one industry interviewee explained that crew will occasionally deploy dFADs at MPA boundaries hoping to aggregate tuna from within (Table 1). However, the degree to which different fleets routinely engage in this practice remains unclear since all industry interviewees (*n* = 5) noted that tuna are only temporary residents of a given protected area and dFAD. Since it can take weeks for tuna to aggregate at a dFAD (*40*, *41*), the ability of fishers to opportunistically exploit an MPA depends on location, size, and prevailing regional currents. While it is legal to deploy a dFAD just outside MPA boundaries, this practice may also increase the likelihood of stranding within an MPA since the device cannot legally be retrieved by a fishing vessel once inside.

### Strandings and retrieval

Combining data from the published literature (*36*, *42*–*47*) and results from our global MPA expert survey (*n* = 49), we document at least 6324 dFAD strandings in 174 protected areas (161 MPAs and 13 shark sanctuaries) across 53 maritime jurisdictions (Fig. 1E and table S2). Thirty percent of MPAs with observed and inferred dFAD strandings (hereafter: “identified MPAs”) are found in the waters of small island developing states; hotspots of reported strandings are clearly visible in the western and central Pacific and western Indian oceans—areas heavily fished with dFADs—but also in the Caribbean (Fig. 1E), which receives hundreds of abandoned dFADs from fishing grounds off West Africa (*48*). The jurisdictions with the most identified MPA strandings were French Polynesia (1539), the Seychelles (1404), and the Maldives (746) (table S2).

**Fig. 3.**
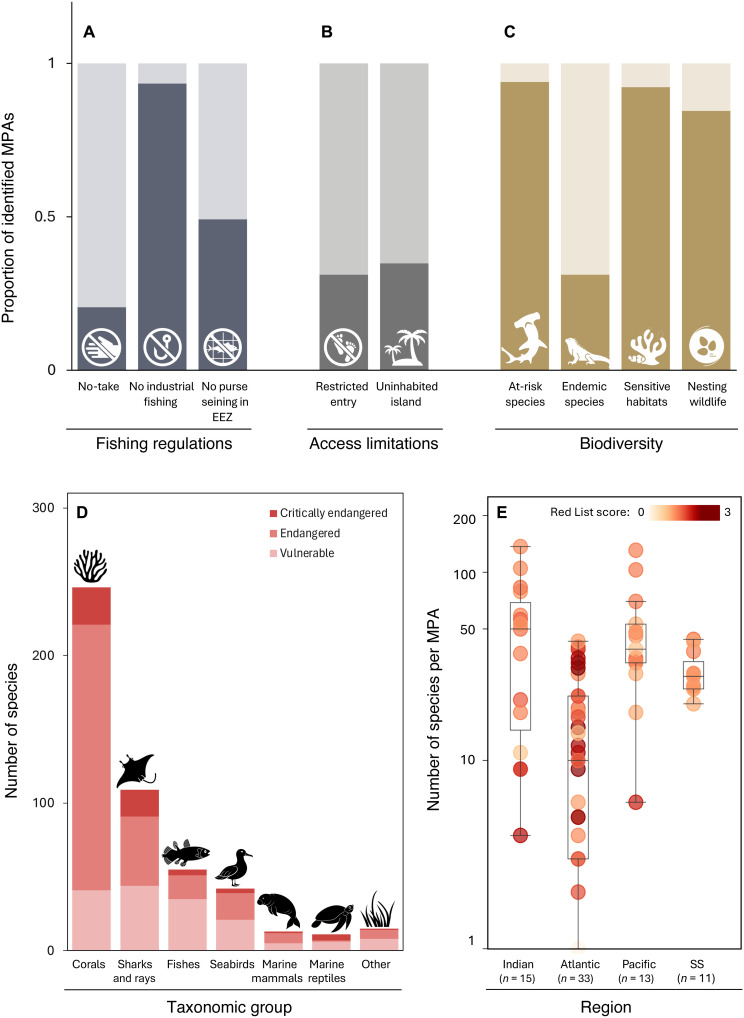
Protective measures and at-risk species in MPAs with dFAD strandings. Dark bars show the proportions of 161 MPAs with identified dFAD strandings where (**A**) all fishing and extraction is prohibited (no-take), industrial fishing is prohibited, and which are found in jurisdictions that do not license purse seining and the use of dFADs. (**B**) Human entry is restricted; no permanent human residency exists; and (**C**) at-risk species, endemic species, sensitive habitats, and nesting wildlife are found. At-risk species in MPAs and shark sanctuaries with observed strandings are shown by taxonomic group and Red List status (**D**). The average number of at-risk species per MPA for each ocean basin is shown in (**E**) with the average Red List score indicated for each MPA by color and the box plot depicting the regional median ± 1.5 interquartile range. Species-level information was available for 74 of 83 MPAs with observed strandings; EEZ, exclusive economic zone; SS, shark sanctuary.

Identified MPAs include some of the world’s oldest, largest, and most remote marine reserves. Reviewing the conservation benefits and access regulations of all identified MPAs with data available, we found that 20% are no-take (i.e., fully protected from all extraction), 93% prohibit industrial fishing, and 49% occur in jurisdictions where dFAD purse seining is not permitted anywhere (Fig. 3A). Further, public entry to 31% of identified MPAs is controlled, and 35% are adjacent to uninhabited islands (Fig. 3B). Regarding biodiversity, we found that 94% of these MPAs protect at least one at-risk species that could be affected by dFAD-related impacts, such as entanglement or plastic ingestion (Fig. 3C). A further 31% include local marine endemics, 92% include sensitive habitats, and 85% harbor nesting turtles or seabirds.

At least 490 at-risk marine species are found in the 83 protected areas with directly observed strandings (Fig. 3D and table S3). While we could not empirically deduce which species have been affected by dFAD interactions in each area, 12 of 13 MPA interviewees documented wildlife caught in dFAD netting within their MPA, especially sea turtles (table S4). At-risk turtles occur in 89% of MPAs with observed strandings—with some providing critical nesting sites (*49*–*51*). These results are consistent with research showing 30 to 60% overlap between dFAD trajectories and endangered sea turtle habitat across the Pacific (*52*). As noted by multiple interviewees, stranded dFAD debris—such as bamboo poles, ropes, and plastic floatation buoys—can present physical barriers to nesting turtles or their offspring (Table 1 and table S4).

Like turtles, sharks and rays have historically been vulnerable to entanglement in dFADs and filter-feeding species, such as manta rays and whale sharks are also susceptible to ingesting microplastics (*53*), a by-product of plastic-based dFAD disintegration. It has also been discussed whether dFADs may draw tropical tuna, and possibly other commonly associated species such as silky sharks, away from productive feeding grounds, through a process referred to as “ecological trap” (*54*). Although research on this topic is substantial, it remains inconclusive (*55*), and we note that one interviewee suggested that a similar process may be occurring for seabirds (Table 1), which have not yet been investigated as part of this hypothesis. Overall, 108 at-risk shark and ray species are found within protected areas with stranding observations (Fig. 3D).

To date, dFAD impacts on corals have been deduced from ocean-scale reef coverage estimates (*45*, *56*, *57*) and do not consider specific species or the consequences of localized losses, which may be borne disproportionately in places with a high occurrence of rare or endemic corals (e.g., countries in the Coral Triangle), frequent strandings (e.g., Seychelles MPAs), or high stranding density (e.g., Palmyra Atoll). We found 246 at-risk coral species in MPAs with observed strandings, 83% of which are listed as endangered or critically endangered (Fig. 3D). Because of their weight and shape, dFAD rafts can get lodged on reefs during low tide or when they sink (Fig. 1B); 12 of 13 MPA practitioners had witnessed coral breakage, entanglement, or smothering, and multiple interviewees noted substantial damage incurred by a single stranded dFAD (Table 1 and table S4).

Additional interactions with sensitive species and ecosystems remain undocumented since not all lost or abandoned dFADs wash ashore. Notably, the number of sunken dFADs remains difficult to estimate, and the fate of >80% of dFADs in the Western Pacific is unknown (*58*). Such interactions contribute to the growing amount of fishing debris on MPA seafloors [e.g., (*59*)] and could potentially cause lasting damage to mesophotic reefs and deep-sea wildlife (Table 1). For example, the critically endangered coelacanth (*Latimera chalumnae*), a living fossil, occurs sporadically along the east African coast but aggregates in the iSimangaliso MPA (*60*). In this MPA, dFADs have been observed on a weekly basis (table S8) and are retrieved by local conservation groups when possible [e.g., (*61*)]. Unfortunately, given the general paucity of data associated with sunken dFADs, the full scope of impacts remains poorly understood for deep-sea environments. Across all protected areas with observed strandings, we found an average of 30.2 ± 29.1 (median = 24) at-risk species per area, with the highest concentration in Indian Ocean MPAs (Fig. 3E).

In addition to these ecological effects, abandoned and stranded dFADs can incur socioeconomic costs (Table 1). Drifting FADs are not the only form of fishing debris that washes ashore in MPAs [e.g., (*30*, *62*, *63*)], but interviewees noted that they are among the most difficult to remove safely because of their size and weight (Fig. 1C). Such removals place a substantial logistical and financial burden on park staff and communities (table S5), and all MPA interviewees noted that this responsibility currently falls on locals rather than purse seine fishery stakeholders. Further, more than half of MPA interviewees observed that removals were often not feasible because of insufficient human capacity, limited resources, or remote stranding locations.

### Solutions

**Fig. 4.**
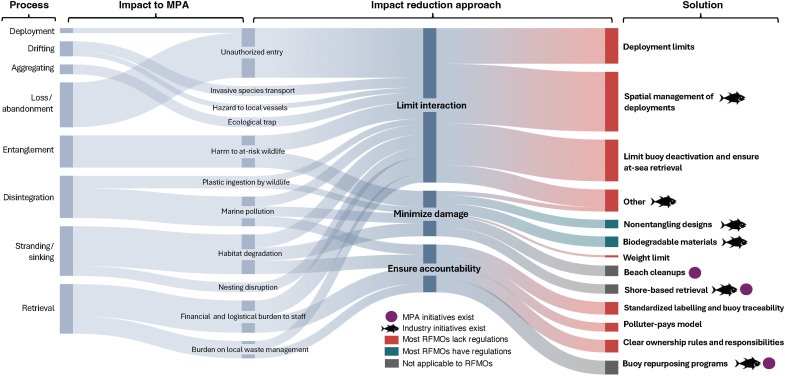
Solutions to limit impacts of dFADs on MPAs. Shown (left to right) are key dFAD life cycle processes, the MPA impacts associated with each, different approaches to reduce such impacts, and related solutions identified by interviewees. Progress with implementation of each solution by four RFMOs, MPA staff, and fishing companies is indicated (see legend). Note that bar width shows relative proportion of interviewee responses for both impacts and solutions. See fig. S1 for schematic of dFAD processes and table S6 for substantial detail on solutions discussed and existing examples.

On the basis of interviewee (*n* = 18) responses, we classified existing and proposed solutions (table S6) for reducing dFAD harm to MPAs into three approaches: (i) minimizing the risk of damage when interaction occurs, (ii) limiting the overall risk of interaction, and (iii) ensuring accountability when interaction and damage occur (Fig. 4). To date, solutions have largely focused on (i), with fishing companies transitioning to nonentangling and biodegradable designs [e.g., (*64*, *65*)]. Interviewees corroborated that this change was initially driven through industry collaboration (*66*–*68*), voluntary best practices (*69*), and market pressure (*70*) (table S7).

#### Minimizing the risk of damage when interaction occurs

Since tuna are highly migratory fishes, formal management of tuna fisheries occurs through regional fisheries management organizations (RFMOs) and fishing regulations—including those for dFADs—are adopted by RFMO member governments at annual meetings. Notably, the number of RFMO management measures related to dFADs has increased 17-fold since 1998 (*23*), and industry-led improvements have recently been accelerated by the adoption of RFMO regulations mandating low-impact designs. For example, while entanglement was highlighted as a concern by 72% of interviewees (table S9), the use of nonentangling dFADs became compulsory in all oceans as of 2025 (table S6). While such RFMO measures do not preclude fishing companies from using materials such as rope, cloth, or canvas, they should measurably reduce the risk of entanglement for sharks and turtles (*71*). The gradual introduction of nonentangling designs in the Indian Ocean appears to have been beneficial for silky sharks, contributing to substantial reductions in dFAD-related mortality for this species over the past decade (*72*). Also at the RFMO-level, a transition to 100% biodegradable designs has been adopted for the Indian and Atlantic by 2028 and 2030, respectively (*73*, *74*). These measures come after over a decade of at-sea fishery trials (*75*), yet some industry members recently expressed concern that the adopted timeline is overly ambitious (*76*). While these regulatory improvements indicate progress in efforts to minimize the risk of dFAD damage, the effectiveness of all RFMO management measures is ultimately contingent upon industry compliance; for dFAD design and labeling requirements, such compliance has historically been low: 18% in the Indian (*42*) and 52% in the Atlantic (*48*). Last, while these recent RFMO regulations stipulate more environmentally conscious designs for dFAD rafts and tails, they do not require modifications to echosounder buoys—which are composed of plastic and electronic components—and which will continue to degrade in the marine environment when they sink or strand.

To further reduce impacts on corals and to address the physical difficulty of retrieving dFADs, some interviewees suggested adopting a standardized weight limit. The recently developed nonentangling and biodegradable “jelly-FAD” is one alternative that could decrease stranding impacts on sensitive habitats, limit marine pollution, and facilitate easier retrieval (*77*), although two industry members expressed concern over its durability (table S6). At present, MPA staff undertake most dFAD beach cleanups (e.g., Fig. 1C), but one-third of interviewees noted that multi-stakeholder partnerships to intercept dFADs have been established in some MPAs. For example, initiatives such as FAD Watch provide MPA staff with an alert when a dFAD is approaching the shore so it can be intercepted before colliding with the reef (*78*). Still, FAD Watch and other shore-based retrieval programs currently remain contingent on the capacity of MPA staff or local conservation organizations as well as participation from willing fishing companies who must choose not to deactivate devices and share their buoy data. As one MPA representative said, “it’s not always practical in a remote place, and it’s a drop in the bucket when you think about the scale of the whole problem. So, I think the answer has to come more from the deployment side” (INT-15).

#### Limiting the overall risk of interaction

Limiting total dFAD deployments, spatially restricting where deployments occur, and ensuring that dFADs are retrieved at sea (*79*) would limit the overall risk of interaction with MPAs (Fig. 4). Industry interviewees expressed support for these approaches (table S9), which is consistent with previous work showing that 96% of fishery stakeholders consider dFAD limits and closures key aspects of sustainable tuna fisheries management (*71*). Currently, all RFMOs limit the number of actively transmitting dFADs per fishing vessel, but no RFMO has explicit regulations in place to limit total or per-vessel deployments. Instead, captains use experiential knowledge and software to help predict dFAD trajectories and avoid strandings, and many buoys now contain floatation sensors that alert crew to compromised devices before they sink (table S6). In the Indian and Atlantic oceans, interactions could be reduced by 20 to 40%—with little impact on tuna catches—by prohibiting deployments in areas that lead to disproportionately high strandings downstream (*80*). Some interviewees suggested that no-deployment buffer zones around MPAs could offer similar protection.

Critically, dFAD abandonment remains a pervasive challenge and 39% of interviewees suggested that better incentives for at-sea retrieval are needed. While the negligent loss of dFADs at sea is a violation of international antidumping legislation (MARPOL, annex V), there remains a legal gray zone when it comes to how “intentional abandonment” of devices is interpreted (*81*). Currently, it is uneconomic for individual vessels or companies to retrieve their dFADs once they drift outside fishing grounds, and most companies do not want to pay for GPS costs associated with lost buoys, which leads to deactivation (table S5). One interviewee also noted that, “when [RFMO] regulations changed, and they limited the number of active FADs [per vessel], that started incentivizing people to deactivate the buoys as soon as they went outside of the main fishing zone [because] even though they know that sometimes their FADs are going to come back, they can’t keep them active and wait” (INT-4). This concern has been previously identified (*71*), yet addressing problems related to deactivation and abandonment remain key shortcomings in existing RFMO regulations, especially since improving at-sea retrieval could notably reduce stranding events [e.g., (*82*)]. It is especially important to collect dFADs before they enter restricted areas since—as highlighted by multiple industry interviewees—dFAD retrieval at sea by a fishing vessel is considered an act of fishing (even if no fish are caught). Hence, it is illegal to retrieve devices in no-take MPAs or maritime jurisdictions where purse seining is not authorized. In general, we note that improved retrieval efforts are also necessary for anchored FADs, which can become dislodged and incur ecological impacts similar to dFADs (*83*).

#### Ensuring accountability when interaction and damage occur

While fishing and buoy companies have recently established programs to collect and repurpose lost and abandoned dFAD buoys in some places (table S6), no large-scale operational model exists for financing dFAD removal, or compensating any ecological damage incurred. Some fleets pay an environmental fee through their fishing license, but this amount is negligible relative to potential dFAD removal costs (*33*) and does not extend to jurisdictions outside of fishing grounds. Four of five industry interviewees felt that companies should be accountable for their dFADs. As one industry member said, “I think once you get to a polluter-pays type model, then there’ll be a lot more attention on it because then there’s a disincentive [to abandonment]” (INT-3). Moving toward such a model is logistically challenging however, since dFAD debris is often unmarked (*45*, *84*). Moreover, some vessels steal or share dFADs, blurring ownership and traceability. Until a comprehensive financing approach is implemented, a standardized retrieval deposit could be included with each buoy purchase, which is reimbursed only if the whole dFAD is retrieved at sea. Appreciably, for this accountability model to be effective, the deposit amount should be in keeping with the substantial cost associated with the removal of a stranded dFAD.

### Study limitations

Our study provides a baseline assessment of dFAD interactions with the global MPA network that can be improved upon with greater industry transparency. Specifically, we were limited by the availability of primary dFAD drift and stranding data and thus we likely present an underestimate of realized impacts. Discussions at RFMO meetings routinely highlight the need for dFAD research to understand impacts (*23*), yet this remains challenging without access to comprehensive GPS buoy data. For example, there are 537 additional MPAs within 15 Indian Ocean jurisdictions where dFAD strandings have occurred (*44*), but we could not determine how many were affected since our request for finer-resolution data was denied because of privacy concerns. Similarly, data on the location of dFAD deployments were not publicly available (except for the Indian Ocean), so we used publicly available RFMO fisheries data to test for preferential dFAD fishing near large-scale MPAs. The coarse spatial resolution of these data (1° grid; ~111 km at the equator) precludes us from identifying the precise location of dFAD fishing events, which may occur hundreds or thousands of kilometers away from where the dFAD is initially deployed. We explored the use of high-resolution Automatic Identification System (AIS) data as an alternative option to identify dFAD fishing events but were unable to reliably differentiate purse-seiners fishing on dFADs from those fishing on free schools. In addition, we saw frequent disabling of AIS by purse seine vessels in key fishing grounds (*85*). These challenges suggest gaps in current fishing effort visualization tools that could be investigated further.

Last, we caution that environmental impacts associated with sunken and stranded dFADs remains vastly underobserved, and many MPAs with observed strandings are typically closely monitored, likely conferring an observation bias. Equally, since we relied on published literature to identify at-risk species for each MPA, not all species groups are consistently covered. While we emphasize that the overlap of at-risk species with observed dFAD strandings shown in this study does not prove a direct impact, we suggest that the critical absence of information on the fate of most dFADs necessitates a precautionary approach. To improve upon our findings, we stress the importance of species-specific data collection as part of all dFAD retrievals and cleanup efforts. In conjunction, detailed records of stranded dFAD materials will be important for assessing regulatory compliance given the recent adoption of RFMO measures requiring nonentangling and biodegradable designs.

### Recommendations

Most MPAs are designed to limit fishery impacts and support wildlife recovery. Our work highlights a fundamental blind spot in this regard: MPAs are not insulated from industrial fisheries when the devices they use drift autonomously across designated ocean boundaries. We find that dFADs released by the global purse seine fleet have likely interacted with at least half of the protected ocean area worldwide, especially in tropical waters. While we do not find strong evidence to suggest that these dFADs are used to systematically extract fish from large MPAs, the deactivation of devices that drift away from fishing grounds substantially limits the potential for vessels to retrieve them before they reach shore, where they may compromise at-risk species. Such abandonment also transfers the burden of retrieval to local MPA staff and communities. More than half of all jurisdictions with identified dFAD strandings are low-income island nations or countries that do not profit from purse seine fisheries but may depend on MPAs for ecological or socioeconomic benefits [e.g., (*86*–*88*)]. Equally, since many island states do rely on funds derived from purse seine fishing, we suggest that continued multisectoral collaborations [e.g., (*79*)] and spatial planning initiatives [e.g., (*89*)] including regional fishery and MPA stakeholders are essential for codevelopment and implementation of practical solutions. Such multilateral work will become even more important with the proposed establishment of MPAs on the high seas under the recently ratified Biodiversity Beyond National Jurisdiction Agreement under the United Nations Convention on the Law of the Sea.

Our work also has implication for the eco-certification of sustainable fisheries. Since almost all dFAD fishing companies now hold MSC certification, they must demonstrate that they do not harm endangered species and ecosystem function. Currently, however, MSC assessments do not consider endangered corals as animals (bycatch) and instead evaluate the potential impacts of dFAD fisheries on coral reefs (habitat). This approach assumes that both the vulnerability and composition of coral species is homogenous for all reefs in a given ocean which, as we observe here, is not the case. Moreover, while we note the substantial progress made by many companies to minimize entanglement risks and plastic pollution (table S6), meeting MSC certification conditions remains challenging for most (*23*). Hence, we suggest an improved RFMO regulatory focus on precautionary deployment limits and other measures that curtail interactions with protected areas outright. This aspect of dFAD management is currently missing but would create immediate benefits to marine ecosystems, threatened species, and coastal communities alike.

## MATERIALS AND METHODS

This study used a mixed-methods approach by incorporating both qualitative and quantitative data in the analysis. Mixed-methods approaches were chosen for their inherent inclusive, pluralistic, and complementary nature, which is especially valuable for data-limited topics (*90*). All aspects of this work involving human participants were approved by Dalhousie University Research Ethics Board (REB# 2023-6982). All survey participants and interviewees participated voluntarily and gave their informed consent in writing before starting the survey or interview. Below, we summarize each of our analyses. For complete information on data and analyses, please refer to the “Detailed methodology” in the Supplementary Materials.

### Inferred interactions with protected areas

To identify global overlap of at-sea dFAD trajectories and the existing MPA network, we superimposed active (i.e., transmitting a GPS signal) dFAD buoy tracks obtained from the literature (*34*–*37*) representing 88,359 devices and binned on a 1°-by-1° grid as reported in (*23*). Buoy tracks from these publications and used for our analysis were from dFADs deployed between 2007 and 2023. We overlayed these tracks with the boundaries of 18,798 MPAs and other effective conservation measures in the ocean obtained from the World Database of Protected Areas (WDPA; https://protectedplanet.net/) in December 2024 to determine what proportion of the global protected area network intersects with dFADs drifting at sea. Given that more than 1.4 million devices were likely deployed globally during this time (*23*), these tracks represent only about 6% of devices at sea, likely rendering our estimates conservative. We note that because of a lack of publicly available dFAD buoy track data (including historic trajectories), there may be a temporal mismatch between when a dFAD buoy drifted through a specific area and when an MPA was established in that same area. However, since dFAD drift trajectories are primarily related to environmental conditions (*34*, *58*), we assumed that trajectories would be consistent even once an MPA was established.

Next, we investigated the incidence of dFAD strandings in WDPA MPAs and shark sanctuaries (*91*) around the world. Data on dFAD stranding locations were obtained from a global online survey of MPA practitioners (in situ observations) and existing peer-reviewed and gray literature (in situ observations and buoy tracks). For the Pacific Ocean, stranding sites not obtained from the survey were inferred from preexisting published documents showing in situ (*45*) and buoy (*36*) stranding densities at a 1°-by-1° spatial scale. Data used for most other areas were available at a finer-scale resolution; see table S2 for the sources associated with each maritime jurisdiction. When amalgamating information from the survey and literature sources, we removed all duplicates to ensure that each MPA was included only once in our analysis. When discussing our results, we explicitly use the term “observed” only in cases where a person has seen a dFAD in a protected area (i.e., documented in the survey or from in situ literature sources where strandings were not estimated). In total, 43% of protected areas with strandings identified had observational data.

To test whether purse seine fisheries could be using dFADs to exploit fish within MPAs, we used publicly available dFAD fishing effort data (number of dFAD sets fished per 1°-by-1° grid cell) from the four tropical tuna RFMOs to (i) map dFAD fishing effort relative to the global MPA network and (ii) identify changes in the proportion of purse seine sets performed on dFADs within 200 nm of large-scale MPA boundaries before and after their establishment. We divided the area around each MPA into four contiguous rings of 50 nm in width and calculated the ratio of dFAD sets to the total number sets occurring within each ring for 5 years before and 5 years following MPA implementation. We then used multiple linear regressions to estimate the changes in %dFAD effort within each ring through time. This procedure was used to estimate an aggregate measure pooling data for all MPAs and then once for each MPA individually.

### Potential impacts of dFAD strandings

For all MPAs with observed or inferred stranding events (collectively: identified MPAs), we used gray and peer-reviewed literature to collect attributes related to three aspects of each protected area: (i) biodiversity (attributes: at-risk species present, endemic species present, nesting wildlife present, and sensitive habitats present), (ii) fishing prohibitions (attributes: no-take MPA, industrial-scale fishing permitted in MPA, and purse seine fishing licensed in adjacent national waters), and (iii) public access (attributes: MPA is adjacent to an uninhabited island, and public entry is monitored or controlled). All attributes were scored using a binary approach (1 = “yes”/”present,” 0 = “no”/”absent”). If information could not be found for a given attribute and MPA, the attribute was classified as “unknown,” and it was not included in the analysis. Overall, we found complete information (i.e., all nine attributes) for 83% of identified MPAs. In addition to scoring the above MPA attributes, we used available literature to identify all at-risk wildlife that could be affected by dFAD interactions for MPAs and shark sanctuaries with observed stranding events. We used the most recent International Union for Conservation of Nature Red List (*92*) as our reference for the conservation status of each species and included those listed as vulnerable, endangered, and critically endangered in our analysis.

### Interviews: Impacts, concerns, and solutions

To complement our quantitative analyses, we conducted semistructured interviews with 13 MPA affiliates (collective experience at 17 MPAs and 1 shark sanctuary), as well as 5 industry affiliates (from 3 tuna purse seining companies and 2 GPS buoy companies). Most MPA interviewees originally self-identified through the survey, and we used a snowball sampling approach to recruit additional interviewees, provided that they were not affiliated with the same organization. Industry interviewees were recruited through preexisting contacts as well as by contacting individuals associated with MSC-certified dFAD fisheries (client group contacts listed on www.fisheries.msc.org) or individuals from the purse seine industry who attended annual RFMO meetings (based on meeting attendance documents). While our sample sizes were modest, they are consistent with similar studies including global fishery stakeholders [e.g., (*70*, *93*)] and well within the range of participant-based studies following the widely accepted approach of achieving response saturation (*94*).

As per well-established approaches to semistructured interviews (*95*, *96*), a set of clearly defined, informed, high-level questions served as the starting point for discussion (see the Supplementary Materials). During a 30- to 45-min interview, interviewees were asked to discuss observed and perceived dFAD impacts to protected areas, challenges in addressing these impacts, and existing or potential solutions (see interview questions in the Supplementary Materials). We deidentified all interviews and systematically coded interview responses for content (i.e., themes) related to perceived impacts and solutions using a binary approach (1 = present, 0 = absent) while retaining quotes for context.

## Supplementary Materials

### This PDF file includes:

Detailed Methodology Figs. S1 to S5 Tables S1 to S10 References
