# Supplementary material for: Global impacts of drifting fish aggregating devices on marine protected areas
Source: Sci Adv. 2026 Jun 17;12(25):eaee6998. doi: 10.1126/sciadv.aee6998 (PMC13274597; doi:10.1126/sciadv.aee6998)
Supplement: Supplementary file 1 — Detailed Methodology Figs. S1 to S5 Tables S1 to S10 References [file sciadv.aee6998_sm.pdf]

Science Advances
AAAS

Supplementary Materials for

Global impacts of drifting fish aggregating devices on marine protected areas

Laurenne Schiller *et al.*

Corresponding author: Laurenne Schiller, laurenne.schiller@dal.ca

Sci. Adv. **12**, eaee6998 (2026)
DOI: 10.1126/sciadv.aee6998

This PDF file includes:

Detailed Methodology
Figs. S1 to S5
Tables S1 to S10
References

Detailed Methodology

Defining protected areas

Our starting definition of 'protected areas' included all coastal and offshore 'Marine Protected Areas' (MPAs) and 'Other Effective Conservation Measures' (OECMs) from the World Database of Protected Areas (WDPA; https://www.protectedplanet.net). This included 18,798 designated marine protected areas and OECMs (excluding Greenland) current through December 2024. We also chose to include the Phoenix Island Protected Area (PIPA) and Chagos large-scale MPAs, which were partly and fully de-gazetted in recent years. Since dFADs have historically incurred high incidental catch of sharks (*26, 97*) we also included shark sanctuaries (i.e. whole exclusive economic zones where shark fishing is prohibited) in our stranding analysis. Our list of shark sanctuaries was current as of 2018 (*91*). Although dFADs are deployed at sea, these devices can affect beaches and associated coastal habitats where they wash ashore. For this reason, we included any 'Terrestrial and Inland Waters Protected Areas' (TIWPAs) in the stranding analysis if these areas extended to the coastline. Lastly, we also included five areas with strandings identified through the survey or literature that are listed as national reserves or refuges but are not included in the WDPA: Southern Line Islands (Kiribati), Oroluk Atoll (Federated States of Micronesia), Tromelin Island/Île Tromelin, Bassas da India, and Juan de Nova Island/Île Juan de Nova. The Southern Line Islands are composed of five atolls, which are national wildlife sanctuaries closed to public entry (*98*), and Oroluk Atoll is included in the Federated States of Micronesia's *Marine Sanctuary and Wildlife Refuge Act of 1999*. Île Tromelin, Bassas da India, and Île Juan de Nova are part of the unpopulated Scattered Islands (Îles Éparses), a group of marine sanctuaries and disputed territory between France and Madagascar, which also includes two areas listed by the WDPA: Archipel des Glorieuses (WDPA ID: 555738609) and Île d'Europa (WDPA ID: 555558368).

Inferred interactions between dFADs and protected areas

1. Drift

1.1. Buoy data sources

Since we did not have access to proprietary dFAD buoy track data, we relied on published records documenting the at-sea trajectories of 88,359 dFAD buoys released between 2007-2023. These publications reported tracks from 9,289 dFAD buoys deployed by 29 French and French-associated (i.e. owned by French fishing companies but flagged to other nations) purse seiners in the Atlantic and Indian Oceans between 2007–2011 (*34*), 56,263 dFAD buoys used by the same fleets between 2012-2018 (*35*), 10,266 dFAD buoys deployed by 123 vessels in the Western and Central Pacific in 2023 (*36*) and12,541 dFAD buoys from 165 vessels in the Eastern Pacific in 2022 (*37*). Given the resolution of data available, we assumed that the deployment behaviour of these fleets and associated buoy drift trajectories were both indicative of dFAD purse seining more generally.

1.2. Visualizing MPAs with potential drift interactions

Figures from each publication documenting the trajectories of dFAD buoys were digitized using Global Information System (GIS) software and combined to obtain a global database of 1° x 1° cells with dFAD track presence or absence. We overlaid this distribution of buoy tracks with the global MPA network shapefiles from the WDPA to determine the overall proportion of the global MPA network likely intersecting with dFAD drift as well as the number of specific MPAs likely affected by dFAD entry. For our area calculations, we removed unique WDPA IDs covering the same area.

2. Stranding

2.1 Data sources

We identified protected areas where dFADs had stranded using published literature and existing databases (table S2), and by collecting observations of dFADs from MPA practitioners in our online survey (see "Online survey questions to MPA affiliates"). Existing stranding records from published peer-reviewed and grey literature included two types of information: (i) in situ observations of dFAD debris, and (ii) strandings inferred from the Global Positioning System (GPS) location of dFAD echosounder buoys (i.e. dFAD track data). Notably, it is impossible to determine from GPS buoy information if the dFAD raft and/or tail were also still present when the device stranded, and thus we made no distinction between intact or disintegrated dFADs in this analysis.

Strandings data for the western Atlantic Ocean (Caribbean and eastern United States) were all based on in situ observations—updated through June 2024—from the Caribbean dFAD Tracking Network database (*43*), while those for the Indian Ocean were obtained from a combination of track data (*44*), and in situ observations *(31, 42, 46, 47, 99).* We were unable to obtain finer-scale resolution buoy track data from (*44*) so we could not determine how many of the total reported strandings (numbers in brackets) from the following jurisdictions occurred within their MPAs: Andaman and Nicobar (700), the Comoros (1), Indonesia (49), Kenya (214), Madagascar (151), Maldives (725), Mauritius (93), Mozambique (132), Oman (36), South Africa (6), Tanzania (156), Thailand (3), and Yemen (109). As such, buoy data from (*44*) for these countries was not used, except for the Maldives since the entire EEZ is a shark sanctuary.

In the Pacific Ocean, data were inferred from published RMFO documents showing dFAD in situ (*45*) and buoy (*36*) stranding densities aggregated at a 1° x 1° spatial resolution. For each cell, standing density was given as a range so we used the mean. We identified MPAs potentially impacted by strandings by overlaying these aggregated data with the Pacific MPA network (shapefiles from WDPA), which resulted in 871 MPAs (i.e. unique WDPA ID) potentially affected. We removed all OECMs for this part of the analysis as well as MPAs listed as 'Proposed'. We further refined our list by including only MPAs larger than 100 km^2 and by removing all cases where a given MPA did not have coastline in a cell with standings. We then calculated the coverage of each MPA in each cell and multiplied this value by its stranding density to estimate the number of dFAD strandings for each MPA per cell. Lastly, we removed all cases where a given MPA had fewer than 0.51 strandings per cell. Since a single MPA can have multiple WDPA IDs/PIDs or a single WDPA ID can have multiple MPAs, we then reviewed all cells and removed duplicate MPAs to avoid double counting (table S10).

To estimate the total number of dFAD strandings for each of the MPAs identified in the Pacific, we assumed in situ strandings and buoy track strandings were discrete events (i.e., no overlap between (*45*) and (*36*). We then calculated the total number of in situ and buoy strandings for each MPA and rounded this value to the nearest whole number. We made five adjustments to our in situ stranding estimates based on area-specific information in (*45*); observed dFAD strandings in brackets: Palmyra Atoll (86), Marae Moana/Cook Islands EEZ (310), Wake Atoll (8), Palau (8) and Pitcairn Islands (21). Based on the aggregated data, our estimated in situ strandings for these areas were 2, 79, 1, 2 and 16, which suggests our approach using the aggregated data is generally conservative. We also note that in using a minimum MPA size (area) criterion, we inherently limited the number of MPAs that could be included in our analysis. By extension, in cases where a large MPA was identified, strandings could actually have occurred in a smaller MPA within, or adjacent to it. For example, the Common fishery right area (Okinawa) in Japan was identified in our approach due to its size (4232 km^2), but five MPAs < 100km^2 found entirely within this MPA were not. As such, our results regarding the number of identified MPAs should also be treated as conservative, especially in maritime jurisdictions where many small MPAs are found.

In addition to estimates and observations from the published literature, we designed a global online survey to obtain information from MPA practitioners. The survey contained eight questions related to participants' dFAD observations within MPAs (see 'Survey Template' at end of methods) and was specifically designed to capture both dFAD presence and absence data as well trends in the occurrence and frequency of dFAD interaction, and MPA affiliates' levels of concern. It was therefore applicable to any region, and we imposed no geographic constraints on which MPAs could be included in the study. Given the specificity of observation-related questions, the survey was also specifically targeted at individuals with a working (on-the-ground) knowledge of the MPA with which they are associated.

The survey was available online through Qualtrics XM from February-May 2024. Initial distribution was through three global mailing lists targeted at MPA practitioners and academics and we subsequently relied on a snowball sampling approach. Overall, the survey was answered at least in part 103 times. From this, we filtered for those surveys that contained sufficient information for our analysis. First, we removed all surveys that were initiated by a participant but lacked any further information (n= 34). Of the remaining 69 surveys, we then removed those where a MPA name was provided but no questions were answered (n = 13). Of the remaining 56 surveys, six were not MPA-specific but regional observations. We did not include these in the analysis but retained their information for context. Lastly, for the 50 surveys that were MPA-specific and contained sufficient information for analysis, we verified that these were all real MPAs by first cross-checking the name in the World Database on Protected Areas (WDPA) and then doing a broader online search. During this process, we found three MPAs not listed on WDPA. One of these ("bilu") was removed since there was no record of it elsewhere (e.g. government website) but we chose to retain surveys for Oroluk Marine Protected Area and the Southern Line Islands (see 'Defining protected areas' above).

The 49 surveys used in our analysis contained information on 44 different MPAs from 23 jurisdictions (table S8). Area covered by MPAs identified through the survey ranged from 0.23 km^2 (Réserve Sous-Marine du Larvotto) to 1.5 million km^2 (Papahānaumokuākea Marine National Monument), with a median area of 2005 km^2. In cases where survey respondents provided optional

comments, we verified that their responses to these questions were in keeping with the responses provided in the multiple-choice questions and that their observations related to drifting FADs from commercial fisheries. As such, comments (where available) were deemed the most accurate form of information and could therefore override question responses where information was contradictory. We made four adjustments to Questions 5 and/or 6 responses based on contextual information provided in the comments (e.g. selected 'Yes' to multiple choice questions on dFAD observations but comments discussed anchored FADs, selected 'No' to multiple choice questions but comments discussed dFAD impacts to MPA reef). These adjustments did not change the overall number of affected versus unaffected MPAs.

We used the WDPA to obtain identification numbers for all MPAs with observed strandings from the survey as well as all others from our literature sources that did not have a WDPA specified. In cases where a stranding location from the literature could apply to multiple different protected areas, the most localized area was chosen. For example, a stranding on "Tintamarre Island, St. Martin" in (*43*) was assigned to Ilet Tintamare (WDPA #555595767) rather than the larger Saint Martin Nature Reserve (WDPA #193403), which also encompasses Tintamarre Island. We also did not include three affected MPAs that were listed as 'Proposed'. There were ten MPAs identified both by surveys and the literature sources and we ensured that each MPA was included only once in our stranding analysis.

2.2. Visualizing MPAs affected by strandings

We mapped all reported dFAD strandings in MPAs using GIS software. Since many maritime jurisdictions currently have low MPA coverage but will be working to increase their protected area coverage in light of Target 3 under the new Global Biodiversity Framework, we also mapped the presence of EEZ-level strandings from (*23*) to indicate other jurisdictions that may face dFAD-MPA conflicts in the future.

3. Fishing

3.1 Mapping dFAD fishing effort

We analyzed the spatial distribution of fishing effort related to drifting Fish Aggregating Devices (dFADs) using dFAD fishing effort data from four Regional Fisheries Management Organizations (RFMOs): Western and Central Pacific Fisheries Commission (WCPFC), Inter-American Tropical Tuna Commission (IATTC), International Commission for the Conservation of Atlantic Tunas (ICCAT), and the Indian Ocean Tuna Commission (IOTC):

- Western and Central Pacific Fisheries Commission (WCPFC): "Aggregated data, grouped by 1° x 1° latitude/longitude grids, year and month," downloaded from https://www.wcpfc.int/sustainability/scientific-data/wcpfc-public-domain-aggregated-catcheffort-data-download-page

- Inter-American Tropical Tuna Commission (IATTC): "Tuna EPO purse seine catch and effort aggregated by year, month, flag or set type, 1° x 1°", version published on April 2023 at https://www.iattc.org/en-US/Data/Public-domain

- International Commission for the Conservation of Atlantic Tunas (ICCAT): "Task 2 catch/effort" published on Jan 1, 2023 at https://www.iccat.int/en/accesingdb.html

- Indian Ocean Tuna Commission (IOTC): IOTC-2023-WGFAD05-DATA01, released on September 29, 2023 at https://iotc.org/documents/fad-activity-data-2013-2022

We filtered the dataset to retain data between 2013 to 2021, and only grid cells with at least one reported dFAD fishing set in this period. The definition of dFAD sets depended on each source of data. In the WCPFC data dFAD sets are explicitly recorded as such. From IATTC data we considered dFAD sets all records labeled "OBJ", implying floating objects. From ICCAT we considered dFAD sets all sets where "set type" was labeled as "FAD". Finally, from the IOTC data, we defined dFAD sets as all records labeled "DRT" (drifting objects with a tracking system), "FAD" and "FDT" (drifting rafts or FAD without a net, and without and with a tracking system), "LGT" (drifting log or debris with a tracking system), and "NFD" and "NFT" (drifting raft or FAD with a net, and without and with a tracking system) from the IOTC data.

The resulting dataset, which includes geographic coordinates (latitude and longitude), was aggregated to calculate the mean annual effort for each 1° × 1° grid cell. Because grid cell area differs by latitude, we calculated the fishing intensity (# of sets / 10,000 km^2) by dividing the effort raster by the cell area. We then reprojected fishing intensity into a Pacific-centered Mollweide projection (EPSG:54009), resampling it to a resolution of 100,000 meters. The data were then log-10 transformed to aid visualization across two orders of magnitude of reported fishing effort (Fig. 2A).

3.2 Visualizing changes in dFAD effort around MPA boundaries

We examined the spatial and temporal changes in the proportion of dFAD fishing effort before and after the implementation of four large-scale MPAs that were located within the area of global dFAD fishing effort: Ascension Island (United Kingdom Overseas Territory), Galápagos (Ecuador), Phoenix Islands Protected Area (PIPA; Kiribati), and Revillagigedo (Mexico). First, we spatially filtered the RFMO effort data to retain all activity outside the MPA but within 200 nautical miles of each polygon boundary. We then filtered the data temporally, keeping all observations within a five-year pre- and post-enforcement period. We then calculated the number of dFAD fishing and total fishing sets that occurred in each 1° × 1° grid cell during the pre- and post- enforcement periods and then computed the percentage of dFAD sets relative to the total purse seine fishing sets within each. We thus have a measure of the relative amount of fishing effort attributable to dFAD fishing for each pixel before and after the MPA was enforced (fig. S2), which allowed us to map pixel-wise changes in proportional dFAD effort since the MPA was implemented (Fig. 2B).

3.3 Estimating changes in dFAD effort around MPA boundaries

To test whether fishers disproportionately engage in dFAD fishing near MPA boundaries following MPA implementation, we first created four concentric 50 nautical mile (nm) rings around MPA boundaries: 0-50 nm, 50-100 nm, 100-150 nm, and 150-200 nm. We retained all RFMO effort data occurring within five years of MPA establishment and inside any of these concentric rings, but not inside the MPAs themselves, ads all of them prohibited industrial fishing.

We then calculated the total number of dFAD fishing sets and total number of purse seine fishing sets per year per ring, and proceeded to calculate relative dFAD fishing effort (i.e. dFAD sets / all sets) for each year and ring. Figure S3 shows a summary of these data.

We were interested in testing the hypothesis that dFAD fishing may have become more frequent near MPA boundaries after the MPA was implemented. However, previous work has shown that the overall use of dFADs has increased over time as well (*19*, *23, 99*) so we had to account for this prevailing temporal trend. We did so by estimating the changes within each ring through time, relative to the changes that we observed in the 150-200 nm ring. That is, we assumed that the 150-200 nm ring captured the regional tendency in dFAD use patterns (fig. S5), but that incentives to fish using dFADs were not affected by the presence of an MPA 200 nautical miles away. We estimated these changes using a multiple linear regression specification, where the dependent variable was the proportion of dFAD effort in ring *i* of MPA *m* at time *t*:

$$y_{imt} = \alpha + \beta_1 Post_t + \beta_{2i} Ring_{im} + \beta_{3i} Post_t * Ring_{im} + \mu_m + \epsilon_{imt}$$

$$y_{it} = \alpha + \beta_1 Post_t + \beta_2 Ring_{im} + \beta_{3i} Post_t * Ring_{im} + \epsilon_{it}$$

Model 1 is used for a dataset pooling data from all MPAs and include the term μ_m, which captures fixed effects by MPA. Model 2 was estimated for each of our four case study MPAs (Ascension Island, Galápagos, PIPA, and Revillagigedo). Across both models, $Post_t$ indicates if the data come from a period before or after the MPA was enforced, and $Ring_{im}$ denotes the ring *i* (0-50 nm, 50-100 nm, 100-150 nm, or 150-200 nm) around the MPA m. Parameter α captures the baseline level of %dFAD fishing in the 150-200 nm ring before the MPA was implemented. β_1 then captures the change in %dFAD fishing the 150-200 nm ring since the MPA was implemented. That is, β_1 provides a baseline level of change in dFAD fishing in the area. β_{2i} then captures the differences between each ring i and the 150-200 nautical mile ring before the MPA was implemented (i.e. the pre-existing trend). Finally, β_{3i} is the coefficient of interest and it captures the temporal change in %dFAD fishing within each ring *i*, relative to the changes observed in the 150-200 nm ring. ϵ_{imt} is the error term in our equation. We estimated this model once for each of the four MPAs. All models were estimated via ordinary least squares using the fixest package (*100*). We used panel-robust standard errors estimated by mpa-ring and year (model 1) and ring-by-year (model 2), following (*101*). All coefficient estimates are shown in table S1.

β_1 captures the mean temporal change in our outcome variable for the ring between 150 and 200 nautical miles. β_2 captures the mean proportion of dFAD sets relative to total sets across the five years leading to MPA implementation. Finally, β_{3i} captures the temporal change in the mean proportion of dFAD sets relative to total sets for rings i = 1:3 relative to the changes already estimated for the furthest away ring. Therefore, β_{3i} are our parameters of interest, because they tell us whether dFAD fishing effort as a proportion of total fishing effort has increased close to MPA boundaries more than it has away from MPA boundaries since the MPA was implemented.

Potential impacts of dFAD strandings

1. Identifying attributes of affected MPAs

For each MPA or TIWPA (hereafter: MPA, unless otherwise specified) identified as having stranding(s), we used grey and peer-reviewed literature to amalgamate attributes related to three aspects of the MPA's location and function: (i) biodiversity, (ii) fishing regulations, and (iii) access limitations. All attributes were scored using a binary approach (1= 'yes'/'present', 0 = 'no'/'absent').

For 'biodiversity', we determined whether: (i) at-risk marine-associated wildlife (permanent or transient) are found within the protected area, (ii) endemic marine wildlife are found within the protected area, (iii) the protected area shoreline is used for breeding by sea turtles or seabirds and (iv) the protected area included coral reefs, mangroves or other sensitive marine habitats (e.g. hydrothermal vents). For the TIWPAs in our analysis, we only included wildlife that would use the coast/beach since these areas do not extend past the shoreline, and we scored all TIPWAs 'Not Applicable' for sensitive marine habitats. We considered species 'at-risk' as those classified as Critically Endangered (CR), Endangered (EN), or Vulnerable (VU) by the IUCN (*92)* and we considered any species found within MPA waters or along the MPA shoreline as 'marine-associated'. Protected area information was obtained directly from government or NGO webpages for the MPA, as well as peer-reviewed and grey literature. If none of these attributes were explicitly mentioned in the source(s) used, we assumed they were absent from the MPA. If no source for a specific attribute could be found for a given MPA, the attribute was coded as 'Unknown' (UNK), and it was not included in the analysis. We found complete information for all four biodiversity attributes for 143 of 161 MPAs, including all TIWPAs marked 'Not Applicable' for attribute (iv).

Regarding 'fishing regulations, we determined whether (i) dFAD fishing was permitted in the wider maritime jurisdiction (i.e. EEZ waters outside the MPA), either by a domestic purse seine fleet or through a foreign access agreement, (ii) if industrial-scale fishing was permitted within the MPA and (iii) whether the protected area was no-take (i.e. fully protected with no extraction permitted). To determine MPA fishing permissions, we used the IUCN Criterion (*2*) such that areas listed as Ia or Ib were classified as 'no take'. When IUCN criteria were not provided for a given MPA, we used information from government websites or regulations and classifications from Protected Seas (https://protectedseas.net/) such that MPAs listed as "Most restrictive" were considered 'no take'. (Information provided by governments superseded that of Protected Seas.) (Notably, while IUCN and Protected Seas provide a standardize metric of comparing MPA intentions and objectives, we acknowledge that these designations do not necessarily correlate with MPA outcomes. As such, further efforts to classify MPAs based their effectiveness in a robust and comparable way remains warranted given recent international commitments to increasing their coverage.) For MPAs with various zones (e.g. the Great Barrier Reef), we used the categorization covering the largest area. For MPAs directly adjacent to the coast (i.e., within 12 nautical miles) without additional information, we assumed that industrial-scale fishing was not permitted. If the protection status could not be determined it was coded as 'Unknown' and not included in the analysis. We scored all TIWPAs as 'Not applicable' for these attributes since they are not designed to protect marine spaces. Using this approach, we found complete information for all three regulatory attributes for 143 of 161 MPAs, including all TIWPAs marked 'Not Applicable'.

Lastly, for 'access limitations' we again relied on MPA websites and other relevant sources to determine whether (i) the MPA encircled or was adjacent to an uninhabited island(s) and (ii) if public access was strictly controlled. We deemed islands 'uninhabited' if there were no local

residents, or if there were only seasonal/transient MPA staff or researchers on site. We considered multi-island MPAs (i.e. large archipelagos) 'uninhabited' if the majority of islands met these criteria. For example, we categorized the Cocos (Keeling) Islands as uninhabited since only two of the 27 islands in the archipelago have permanent residents. Many of the MPAs affected by dFAD strandings are in some of Earth's most remote locations thus making them inherently inaccessible to most people. However, our definition of 'controlled access' related to visitation permits/tickets for entry to the MPA (including outright visitation prohibitions) or if visitor quotas existed. For example, many tourist and research activities in the Great Barrier Reef require permits, but since personal use of the area (e.g. recreational swimming and boating) is allowed without a permit we deemed this was not a case of controlled access. If MPAs were located directly adjacent to populated areas or communities, it was assumed there were no restrictions on public entry (unless explicitly stated otherwise). We found complete information for both attributes for all 161 MPAs.

2. Documenting endangered wildlife found within affected MPAs

We used available literature to identify all at-risk marine wildlife in each affected MPA, TIWPA, and shark sanctuary with observed strandings. Marine species groups that met this criterion included: fishes (pelagic, reef-associating, and deep-water), corals, marine mammals, sharks and rays, seabirds, shorebirds, and marine reptiles. To be precautionary, we also included other species that could be impacted based on their specific habitat preferences (e.g. coastal terrestrial reptiles, non-coral marine invertebrates). We only included seabirds and turtles for the TIWPAs since these areas do not extend into the marine environment. For all shark sanctuaries, we only included sharks and rays since they are the only wildlife explicitly protected by the sanctuary.

We used the most recent IUCN Red List (*92*) as our reference for the conservation status of each species. We included those listed as Vulnerable (VU), Endangered (ER), and Critically Endangered (CR) and matched species using their Latin name. In cases where Latin names differed between the document reviewed and the IUCN Red List due to taxonomic classification changes, we deferred to the IUCN. When only a Family or Genus name was given in a source document, the species was not included and, where documents explicitly stated that a species was 'rare' or 'occasional' in the MPA, it was also not included.

All efforts were made to ensure we used MPA-specific rather than country-specific information and when sources delineated species (often corals) at multiple sites within an EEZ, we only included species at sites within or closest to affected MPA. For ten shark sanctuaries we relied on country-level elasmobranch species lists obtained from the IUCN since no other sources providing information at this scale could be found. We could not find information for all taxonomic groups in many MPAs, which likely renders our species list conservative. Both Palau and the Cook Islands have EEZ-wide protected areas and shark sanctuaries, but species found in these jurisdictions were included only once in our analysis to avoid double-counting.

3. Visualizing impacts

We plotted the relative proportion of all MPAs with available information based on the presence or absence of each biodiversity, fishing regulation, and access limitation attribute. For impacted at-risk species, we plotted the total number of unique species in each taxonomic group by conservation status. To each IUCN status we assigned the following scores: 1= Vulnerable, 2= Endangered, 3= Critically Endangered and plotted the average score based on all at-risk species documented for each MPA in each ocean basin.

MPA and industry affiliate interviews: Impacts, concerns, and solutions

As part of the survey, respondents were provided the option to participate in a follow-up interview. A total of 28 people left contact information but in keeping with the focus of the study, we chose to include only individuals affiliated with MPAs where dFADs have been observed. Of those contacted, nine responded and consented to being interviewed. In conjunction with the interviews, a snowball sampling approach was used to recruit additional interviewees. This approach resulted in a total of 13 MPA-affiliated interviewees with experience in 17 MPAs and one shark sanctuary included for in-depth discussion. This equates to 25% of protected areas with observed strandings as identified in our literature review. The protected areas discussed by interviewees are located in all ocean basins (7 in the West Pacific, 2 in the East Pacific, 3 in the Atlantic, and 5 in the Indian) and range in size from <10 km^2 to $>100,000$ km^2 (median =1997 km^2). Regarding fishing regulations, all protected areas discussed by interviewees prohibit industrial-scale fishing and nine are ‘no-take’ (i.e. fully protected from all extraction).

In addition to interviewing MPA affiliates, we sought a balanced perspective by seeking feedback from members of the purse seine industry (i.e. companies that use dFADs). We contacted individuals associated with companies in MSC-certified client groups using information available on the MSC assessment or website and those from other companies based on information on publicly available RFMO meeting attendance documents. We again relied on a snowball approach to recruit additional interviewees. Across 38 companies contacted, five industry affiliates agreed to an interview, three of whom were affiliated with different purse seine companies (using both free school and dFAD fishing methods) and two from GPS buoy provider companies. All industry affiliates are well acquainted with the day-to-day operations of purse seine vessels at-sea.

For all MPA and industry affiliates, interviews were semi-structured, and questions focused on the challenges dFADs pose to MPA conservation and management, observed impacts, existing and future solutions (see "Interview questions to MPA and industry stakeholders"). LS conducted all interviews, each of which lasted 30-45 minutes. All 18 interviewees except one (INT-10) consented to having the interview audio-recorded and the use of direct quotes. Recordings were transcribed by LS and interviewees were de-identified and assigned an arbitrary numerical identifier. To protect the anonymity of interviewees, references to specific companies, MPAs, countries, people, or other information that could enable identification were removed from all interview transcripts and we intentionally refrain from including any of this information in our paper.

Interview content was manually coded in binary fashion to reflect broad themes discussed by interviewees. For our interview analysis, each interviewee’s comments were scored in a binary fashion (1 = mentioned, 0 = not mentioned) for (i) observed and perceived dFAD impacts to MPAs and (ii) existing or potential solutions to address impacts (within and beyond MPAs). The relative weight of each impact shown in Fig. 4 is proportional to the number of interviewees mentioning

that impact. We assigned all impacts into one or more of the following impact reduction approaches: (i) limit interaction with MPA, (ii) minimize damage to MPA/wildlife when interaction occurs, and (iii) ensure accountability when damage occurs. (When impacts were assigned to two or three approaches, it was equally weighted between them). Each solution identified by the interviewees was assigned to one of these approaches. The relative weight of each solution in Fig. 4 is proportional to the relative weight of the impact reduction approach (i.e. how many impacts would be addressed by [i], [ii] or [iii]) and, within that, the number of respondents mentioning each solution. Unweighted interviewee responses are summarized in table S9.

Lastly, as part of our solutions analysis, we amalgamated existing RFMO solutions by reviewing each RFMO's most recent regulations containing dFAD provisions, i.e. IOTC: Res. 24/02 ; IATTC: C-24-06, C-24-01, C-23-05, C-23-04; ICCAT: Rec. 24-01; and WCPFC: CMM 2009-02 and CMM 2023-01.

Online survey questions to MPA affiliates

Note: study rationale and request for participant consent provided on survey landing page prior to questions.

___

1. (Open ended) Please write the **name of the MPA** you are affiliated with and kindly answer all survey questions in relation to this MPA only. (If you would like to give information on multiple MPAs, please do so by completing a separate survey for each.)

2. (Open ended) Please provide the **country of this MPA**.

3. (Multiple choice) Please select your primary affiliation with regard to this MPA:
    - Government
    - Non-governmental organization
    - Indigenous community
    - Industry
    - Academia
    - Volunteer
    - Other (please specify):

4. (Multiple Choice) How long have you been involved with this MPA?
    - 1 year
    - 2 years
    - 3 years
    - 4 years
    - 5 years
    - More than 5 years

5. (Multiple Choice) Have drifting FADs been **observed floating** within this MPA in the time you have worked there?
    - Yes
    - No
    - Unsure

6. (Multiple Choice) Have drifting FADs **washed ashore** within this MPA in the time you have worked there?
    - Yes
    - No
    - Unsure
    - Not applicable (this MPA lacks islands/atolls/coastline)

7. (Multiple Choice) How frequently do drifting FADs **wash ashore** in this MPA?
    - Weekly

- Monthly
- Periodically (2-10 times per year)
- N/A (I have never seen one wash ashore in this MPA)

8. (Multiple Choice) Do you observe a trend in the frequency of drifting FADs **washed ashore** in this MPA since you started working there?
    - Increasing
    - Decreasing
    - No change (stable)
    - Unsure
    - N/A (I have never seen one wash ashore in this MPA)

9. (Multiple Choice) Do you observe a trend in the frequency of drifting FADs **floating** in this MPA since you started working there?
    - Increasing
    - Decreasing
    - No change (stable)
    - Unsure
    - N/A (I have never seen one floating in this MPA)

10. (Multiple Choice) Overall, how concerned are you about the effects of drifting FADs on your MPA?
    - 1 (No concern)
    - 2 (Low concern)
    - 3 (Moderate concern)
    - 4 (High concern)
    - 5 (Very high concern)

11. (Optional – open ended) Please specify any concerns or observations you have (e.g. condition of drifting FADs when they wash ashore, whether satellite buoys and transponders are still attached, whether the drifting FAD could be traced to a specific company, etc.) or provide additional feedback regarding drifting FADs and marine protected areas based on your experience or perception.

12. (Optional – open ended) If you are interested in participating in a follow-up interview, please provide your email address here:___________________________

Interview questions to MPA and industry stakeholders

Note: since not all questions apply to the two stakeholder groups in the same way, some adjustments were made depending on the affiliation of the interviewee. Questions posed to industry stakeholders are show in bold. Since all interviews were semi-structured, these questions served as a starting point for the conversation but, depending on responses, there were additional follow-up questions to gain further context, clarification, or examples. In keeping with their consent to participate, no interviewee was obligated to answer any question they were uncomfortable with, and all interviewees were explicitly asked to highlight instances when they did not know or had a high level of uncertainty in their response.

——

WELCOME (~5 minutes) : Introduction with discussion of participant consent, study scope and objective, overview of how interview will proceed, opportunity for interviewee to ask any questions.

QUESTION PERIOD (~30-40 minutes)

<u>BACKGROUND</u>

- How long have you been involved with this MPA? **How long have you worked for this company**?

- What is your basic role with the MPA and how involved are you with the day-to-day operations of management, assessment and enforcement? **What is your basic role with the company and how involved are you with the day-to-day operations of vessels at sea?**

<u>IMPACTS</u>

- Have you observed dFADs drifting through this MPA? If yes, when, and how close to shore? (MPA stakeholders only)

- **Do vessel captains ever deploy dFADs near MPA perimeters? If yes, why do they do this? (Industry stakeholders only)**

- Have there been any dFAD stranding events in this MPA? Who was responsible for removing the gear? Were any identifying features on the dFAD specific to a country, company or vessel? (MPA stakeholders only) **Have you received any reports of one of**

your company's dFADs stranded within a MPA? If yes, how are these reports handled by your company?

- What are the biggest impacts from dFADs on this MPA that you have seen or are aware of? **What are the biggest impacts from dFADs on MPAs that you have seen or are aware of?**

- Do you suspect dFADs are aggregating and then removing fish from this MPA? If yes, why? If no, why not? **Do you suspect dFADs are aggregating and then removing fish from MPAs? If yes, why? If no, why not?**

- How concerned are you about the impacts of dFADs relative to other challenges facing the MPA? (MPA stakeholders only)

<u>SOLUTIONS</u>

- Has there been any attempt by a fishing company or your government to address dFAD impacts on your MPA? If yes, what solutions have been proposed or attempted? If no, what solutions would you like to see? **What efforts has your company taken to mitigate dFAD stranding events and other impacts? How is your company working to ensure dFADs have a low environmental impact?**

- Who should be financially responsible for removing stranded dFADs? (**Same question to both groups**)

- **Does your company work directly with any governments or conservation agencies to develop novel solutions to dFAD impacts? If yes, what are they and how promising are these efforts? (Industry stakeholders only)**

- What are the best regulatory measures, either at the RFMO level or domestically, to limit dFADs in MPAs? (**Same question to both groups**)

ANY FINAL COMMENTS?

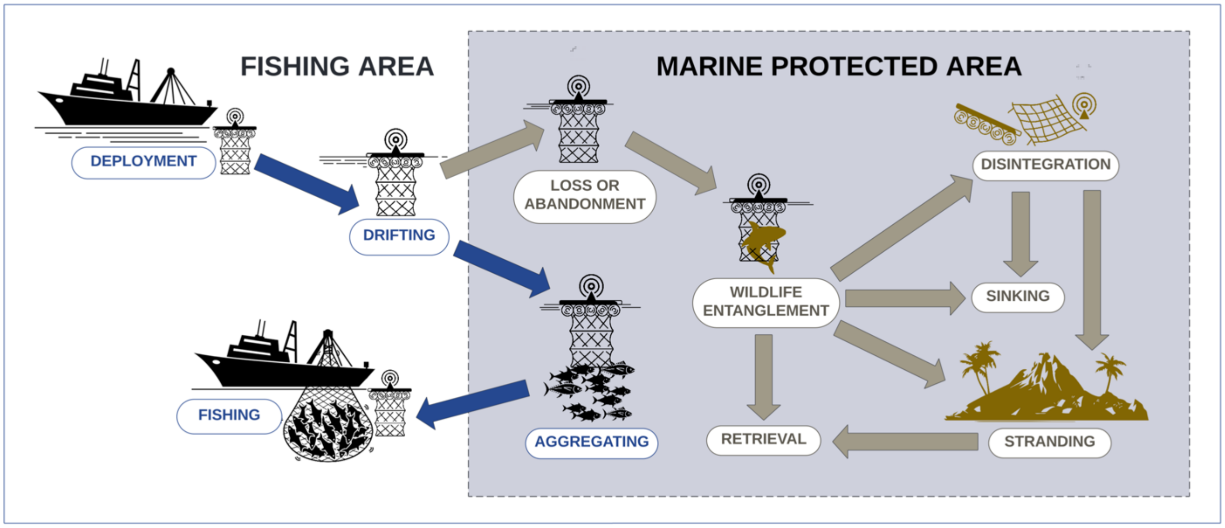

Fig. S1. Potential pathways of impact to marine protected area (MPA) incurred by fishing with drifting Fish Aggregating Device (dFAD; traditional design).

Once deployed from a purse seine vessel, an active dFAD may enter a marine protected area where it aggregates target tuna and non-target species before drifting back outside the protected area where it gets fished (blue pathway). Alternatively, a deployed dFAD may also be lost or abandoned (remotely de-activated) by vessel crew once it enters the protected area, where it will continue to drift, aggregating and entangling non-target species. Ultimately, the lost dFAD will disintegrate, sink, become stranded, or be retrieved by local government officials, conservation organizations, or MPA staff (grey pathway). Parts of disintegrated dFADs may sink or wash ashore separately. All intact dFADs and dFAD parts that sink to depths beyond the immediate shoreline are likely never retrieved while those that strand on beaches or reefs can be retrieved if resources allow.

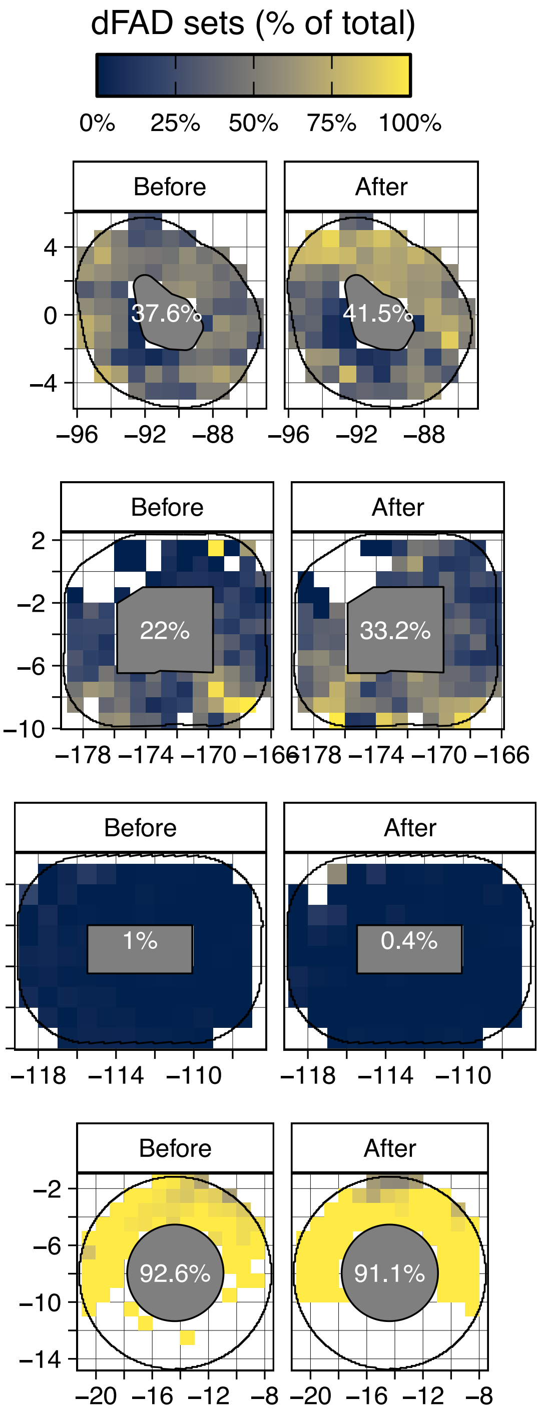

Figure S2. Drifting FAD fishing effort within 200 nautical miles of MPA boundaries as a proportion of total effort before and after MPA implementation. Numbers in the center show the average proportion of dFAD fishing effort across all pixels.

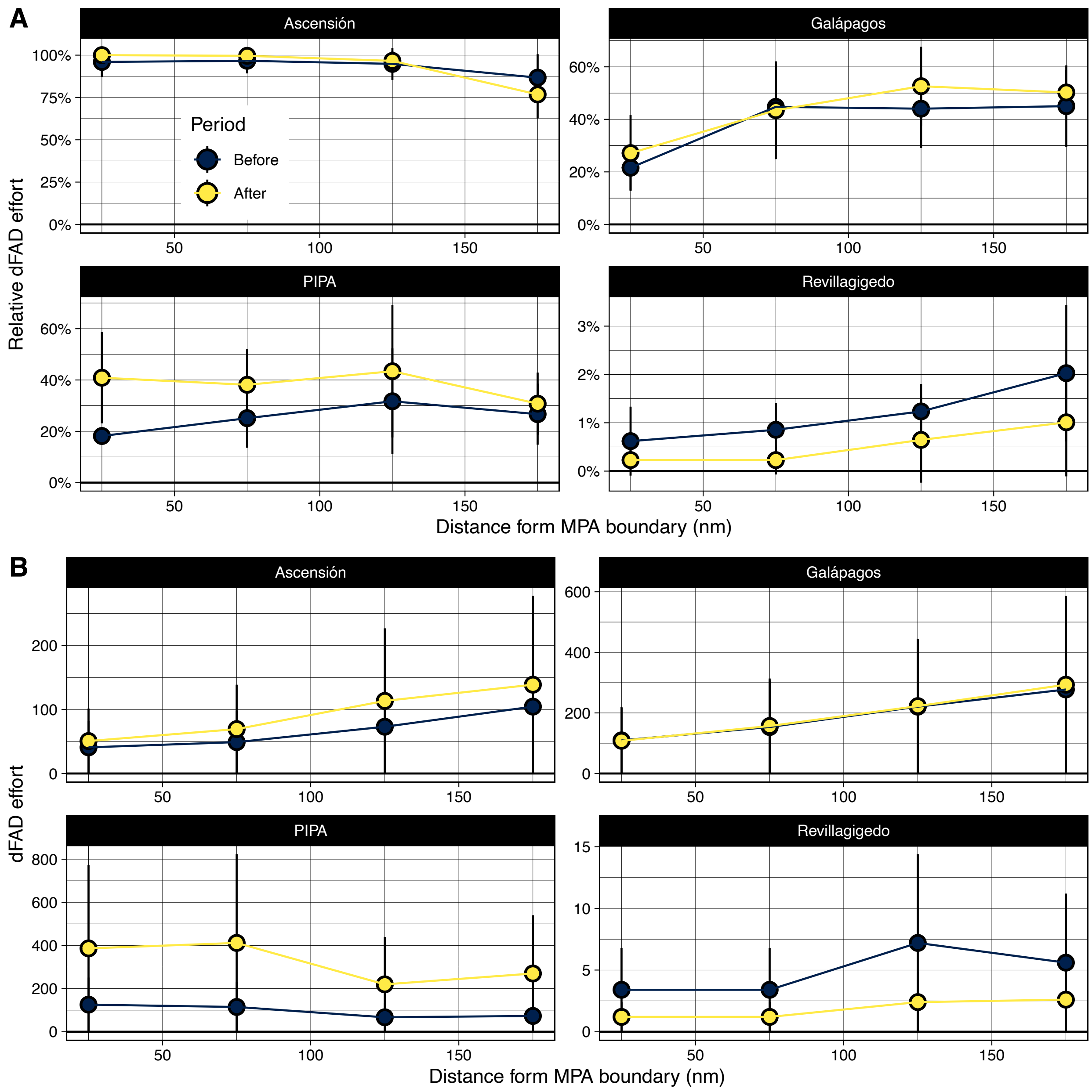

Figure S3. Change in Fish Aggregating Device (%dFAD) fishing through time (colors) and across space (x-axis) for four large-scale marine protected areas. Panel A shows data in relative terms, and Panel B shows data in absolute numbers. Points show the mean values for each distance bin and period, and error bars show standard deviations.

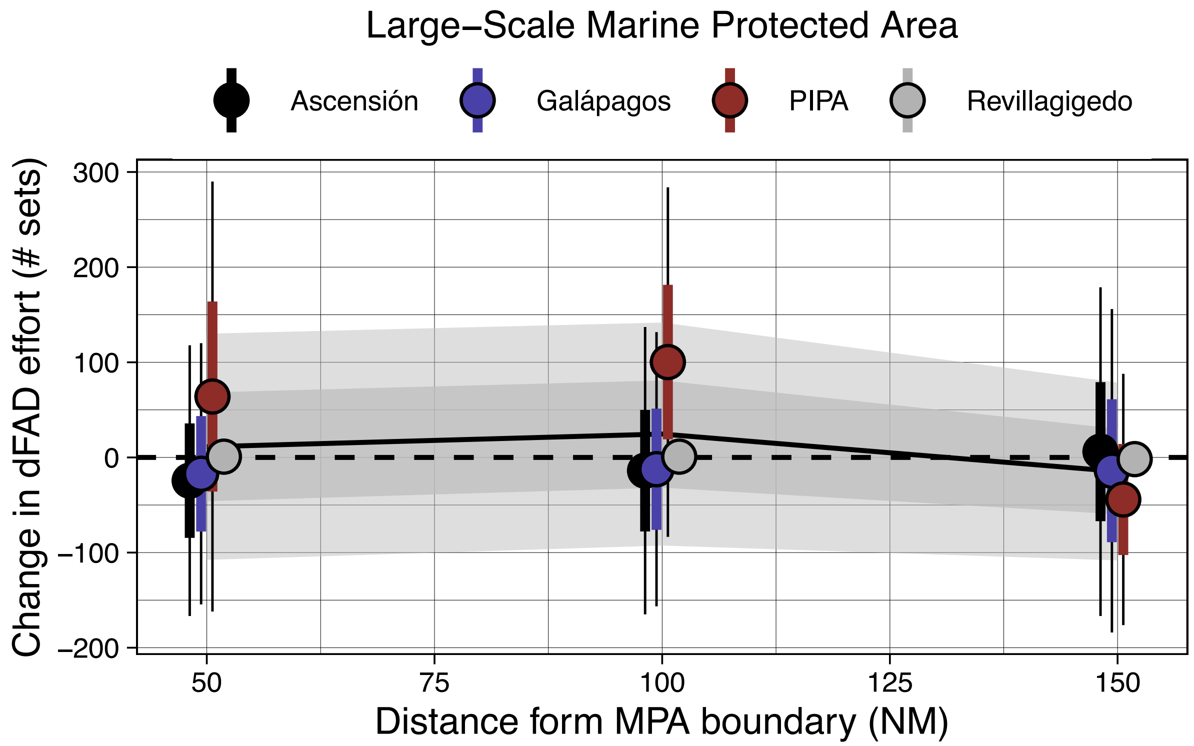

Figure S4. Change in the number of dFAD sets by distance bins. Points represent the estimate, thick portion of the bars are standard errors, and thin portion of the bars are 95% CI.

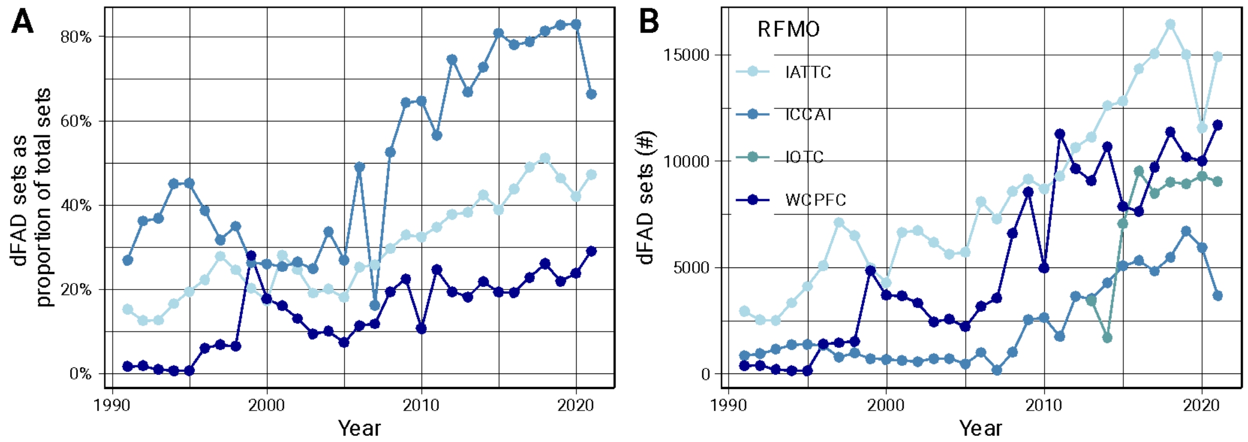

Figure S5. Time series of dFAD fishing effort. (A) As a proportion of total reported sets and (B) in absolute numbers.

Table S1. Table S8. Coefficient estimates for linear model testing for changes in percent drifting Fish Aggregating Device (%dFAD) effort near MPA boundaries. Numbers in parentheses are panel-robust standard errors. For MPA-level regressions (columns 1-4), standard errors are calculated at the ring-by-year level. For pooled regression (column 5) standard errors are calculated at the MPA-by-ring-year level. Note: PIPA = Phoenix Island Protected Area.

	Ascension	Galápagos	PIPA	Revillagigedo	Pooled
Intercept	0.867***	0.450***	0.267***	0.020***	
	(0.048)	(0.059)	(0.042)	(0.006)	
after	-0.099	0.052	0.041	-0.010	0.013
	(0.083)	(0.073)	(0.074)	(0.008)	(0.044)
150 nm ring	0.081	-0.010	0.050	-0.008	0.028
	(0.068)	(0.083)	(0.092)	(0.006)	(0.031)
100 nm ring	0.099	-0.003	-0.016	-0.012*	0.017
	(0.060)	(0.091)	(0.068)	(0.006)	(0.033)
50 nm ring	0.093	-0.234***	-0.085*	-0.014*	-0.060
	(0.064)	(0.069)	(0.046)	(0.006)	(0.044)
after:150 nm ring	0.117	0.034	0.076	0.004	0.045
	(0.097)	(0.115)	(0.142)	(0.010)	(0.060)
after:100 nm ring	0.129	-0.065	0.089	0.004	0.020
	(0.090)	(0.132)	(0.115)	(0.009)	(0.057)
after:50 nm ring	0.139	0.004	0.186	0.006	0.061
	(0.093)	(0.107)	(0.126)	(0.009)	(0.073)
Num.Obs.	32	40	40	40	152
R2	0.401	0.387	0.241	0.364	0.874
R2 Adj.	0.226	0.253	0.074	0.225	0.865
Std.Errors	Newey-West (L=1)	Newey-West (L=1)	Newey-West (L=1)	Newey-West (L=1)	Newey-West (L=2)
FE: mpa					X

· $p < 0.1$, ** $p < 0.05$, *** $p < 0.01$

Table S2. Maritime jurisdictions (exclusive economic zones, EEZs) with identified dFAD strandings in marine protected areas (MPAs). Jurisdictions with only inferred strandings (i.e. derived from 1° x 1° spatial data) are italicized and affected MPAs that are also EEZ-wide shark sanctuaries marked with an asterisk (*); shark sanctuaries without identified MPAs are noted with 'SS'. 'Stranded dFADs' includes both inferred and observed values from the sources listed (for surveys and in cases from the literature where observation(s) exist but no concrete estimate is available, the greater than or equal to sign is used). Jurisdictions classified by the United Nations as 'Small Island Developing States' (SIDS) are also indicated.

**Maritime jurisdiction**	**MPAs identified with strandings (*n*)**	**dFADs stranded in MPAs (*n*)**	**SIDS**	**Source(s)**
Ascension Island (UK OST)	1	≥2	No	(*31, 44*)
Antigua and Barbuda	1	1	Yes	(*43*)
Australia	*15*	*260*	*No*	(*36, 45*)
Bahamas (*)	2	24	Yes	(*43*)
Bassas da India (France OST – disputed)	*1*	*486*	*No*	(*44*)
Bonaire (Netherlands OST)(*)	0 (SS)	1	No	(*43*)
Brazil	1	2	No	(*43*)
British Virgin Islands (UK OST)(*)	0 (SS)	3	No	(*43*)
Chile	1	≥1	No	Survey
Cook Islands (*)	1	364	Yes	(*36, 45*)
Costa Rica	2	≥3	No	Survey, (*36*)
Ecuador	1	≥153	No	Survey, (*102*)
Europa Island (France OST)	*1*	*2*	*No*	(*44*)
Federated States of Micronesia (*)	2†	≥189	Yes	Survey, (*45*)
Fiji	*8*	*9*	*Yes*	(*36*)
French Polynesia (France OST)(*)	0 (SS)	1539	No	(*45*)
Gabon	1	≥1	No	Survey
Glorioso Islands (France OST)	1	≥22	No	Survey, (*44*)
Guadeloupe	*1*	*1*	*No*	(*43*)
Indonesia	*15*	*26*	*No*	(*36*)
Japan	*6*	*7*	*No*	(*36*)
Juan de Nova Island (France OST – disputed)	*1*	*19*	*No*	(*44*)
Kenya	3	10	No	(*42, 47*)
Kiribati (*)	2	≥376	Yes	Survey, (*102*)
Korea	*5*	*6*	*No*	(*36*)
Maldives (*)	0 (SS)	746	Yes	(*42, 44*)
Marshall Islands (*)	*3*	*107*	*Yes*	(*36, 45*)
Mauritius (formerly UK OST)	1‡	123	Yes	(*42, 44, 46*)
Mayotte (France OST)	1	≥13	No	Survey, (*44*)
Mexico	2	3	No	(*43*)
New Caledonia (France OST)(*)	6	141	No	(*36, 45*)
Oman	1	1	No	(*44*)
Palau (*)	3	≥19	Yes	Survey, (*36, 45*)
Panama	*2*	*4*	*No*	(*36*)
Papua New Guinea	*3*	*11*	*Yes*	(*36, 45*)
Philippines	*4*	*6*	*No*	(*36*)
Pitcairn Islands (UK OST)	2	23	No	Survey, (*44*)

Puerto Rico (USA OST)	1	1	No	(*43*)
Saint Lucia	1	1	Yes	(*43*)
Saint Martin (France OST)	1	1	No	(*43*)
Saint Vincent and the Grenadines	1	3	Yes	(*43*)
Saint-Barthélemy (France OST)	1	1	No	(*43*)
Samoa (*)	0 (SS)	28	Yes	(*45*)
Seychelles	22	≥1404	Yes	Survey, (*42, 44*)
Sint Maarten (Netherlands OST)(*)	0 (SS)	1	No	(*43*)
Solomon Islands	*3*	*25*	*Yes*	(*36, 45*)
South Africa	5	≥5	No	Survey, (*47*)
Taiwan	*2*	*4*	*No*	(*36*)
Tromelin Island (France OST - disputed)	*1*	*1*	*No*	(*44*)
Turks and Caicos Islands (UK OST)	1	2	No	(*43*)
UK	1	1	No	(*43*)
US Virgin Islands (USA OST)	2	2	No	(*43*)
USA	19	≥140	No	Survey, (*36, 43, 45*)
**TOTALS: 53 jurisdictions**	**172 (161 MPAs + 13 SS)**	**≥ 6,324**	**16 SIDS**	

†Two respondents completed the survey for two separate MPAs in the Federated States of Micronesia: Oroluk Atoll and Ahnd (also known as ‘And’ or ‘Ant’) Atoll, only the second of which is included in Protected Planet (WDPA ID #555547540). However, since Oroluk is protected through regional regulations (Pohnpei State Code, Title 26, Chapter 5), we retained both for our analysis.

‡This refers to the Chagos Archipelago (also known as ‘British Indian Ocean Territory’). In 2015, it was determined that the UK government failed to properly consult Mauritius when it established the MPA in 2010. In 2019, the International Court of Justice advised that the UK “is under an obligation to bring to an end its administration of the Chagos Archipelago as rapidly as possible”. In 2021, the International Tribunal for the Law of the Sea (ITLOS) ruled that Mauritius holds the sovereign rights to the Chagos Archipelago and the MPA has since been de-gazetted.

Table S3. At-risk wildlife (*n*= 490 species) that could be impacted by dFADs found in marine protected areas (MPAs) and shark sanctuaries with observed strandings (*n*= 76). 'Status' refers to species' conservation status from the International Union for Conservation of Nature (IUCN) Red List of Threatened Species (v. 2025-1) where VU = Vulnerable, EN = Endangered, CR = Critically endangered; 'Occurrence' refers to the total number of MPAs with observed strandings where the species occurs but is likely under-estimated due to incomplete information for all taxonomic groups in many MPAs. Note: since we took a precautionary approach to the inclusion of all-marine associated wildlife, we mark with an asterisk (*) the 14 species for which no impacts to their *species group* have thus far been recorded in the literature nor were mentioned by any interviewees. See Table 1 and table S4 for examples of observed impacts to certain species and groups as reported by interview participants.

Species group	Common name	Latin name	Status	Occurrence (*n*)
Birds	African penguin	*Spheniscus demersus*	EN	2
Birds	Aldabra Fody	*Foudia aldabrana*	EN	1
Birds	Ascension frigate birds	*Fregata aquila*	VU	1
Birds	Atlantic yellow-nosed albatross	*Thalassarche chlororhynchos*	EN	2
Birds	Bank cormorant	*Phalacrocorax neglectus*	EN	2
Birds	Black petrel	*Procellaria parkinsoni*	VU	1
Birds	Broad-billed sandpiper	*Calidris falcinellus*	VU	1
Birds	Buller's shearwater	*Ardenna bulleri*	VU	1
Birds	Cape cormorant	*Phalacrocorax capensis*	EN	2
Birds	Cape gannet	*Morus capensis*	EN	2
Birds	Collared petrel	*Pterodroma brevipes*	VU	1
Birds	Common Redshank (European pop.)	*Tringa totanus*	VU	1
Birds	Cook's petrel	*Pterodroma cookii*	VU	1
Birds	Curlew sandpiper	*Calidris ferruginea*	VU	2
Birds	Far Eastern curlew	*Numenius madagascariensis*	EN	1
Birds	Flightless cormorant	*Nannopterum harrisi*	VU	1
Birds	Galapagos penguin	*Spheniscus mendiculus*	EN	1
Birds	Galapagos petrel	*Pterodroma phaeopygia*	CR	2
Birds	Great knot	*Calidris tenuirostris*	EN	1
Birds	Grey plover	*Pluvialis squatarola*	VU	5
Birds	Henderson petrel	*Pterodroma atrata*	EN	2
Birds	Indian yellow-nosed albatross	*Thalassarche carteri*	EN	2
Birds	Juan Fernandez petrel	*Pterodroma externa*	EN	1
Birds	Lava gull	*Larus fuliginosus*	VU	1
Birds	Leach's storm-petrel	*Oceanodroma leucorhoa*	VU	2
Birds	Lesser yellowlegs	*Tringa flavipes*	VU	4
Birds	Madagascar Pond-heron	*Ardeola idea*	EN	3

Birds	Noronha elaenia	*Elaenia ridleyana*	VU	1
Birds	Phoenix petrel	*Pterodroma alba*	VU	2
Birds	Pink-footed shearwater	*Puffinus creatopus*	VU	1
Birds	Polynesian storm petrel	*Nesofregetta fuliginosa*	EN	2
Birds	Pycroft's petrel	*Pterodroma pycrofti*	VU	1
Birds	Short-billed dowitcher	*Limnodromus griseus*	VU	1
Birds	Siberian Sandplover	*Charadrius mongolus*	EN	1
Birds	Tristan albatross	*Diomedea dabbenena*	CR	1
Birds	Wandering albatross	*Diomedea exulans*	VU	1
Birds	Waved albatross	*Phoebastria irrorata*	CR	2
Birds	Westland petrel	*Procellaria westlandica*	EN	1
Birds	White-chinned petrel	*Procellaria aequinoctialis*	EN	2
Birds	White-necked petrel	*Pterodroma cervicalis*	EN	1
Birds	White-rumped sandpiper	*Calidris alpina*	VU	1
Birds	White-winged petrel	*Pterodroma leucoptera*	VU	1
Corals	*Acanthastrea brevis*	*Acanthastrea brevis*	VU	4
Corals	*Acanthastrea hemprichii*	*Acanthastrea hemprichii*	VU	3
Corals	*Acropora abrolhosensis*	*Acropora abrolhosensis*	EN	1
Corals	*Acropora abrotanoides*	*Acropora abrotanoides*	EN	6
Corals	*Acropora aculeus*	*Acropora aculeus*	EN	9
Corals	*Acropora acuminata*	*Acropora acuminata*	VU	5
Corals	*Acropora anthocercis*	*Acropora anthocercis*	EN	5
Corals	*Acropora arabensis*	*Acropora arabensis*	EN	2
Corals	*Acropora aspera*	*Acropora aspera*	EN	3
Corals	*Acropora austera*	*Acropora austera*	EN	7
Corals	*Acropora awi*	*Acropora awi*	EN	1
Corals	*Acropora brueggemanni*	*Acropora brueggemanni*	EN	1
Corals	*Acropora carduus*	*Acropora carduus*	EN	1
Corals	*Acropora caroliniana*	*Acropora caroliniana*	VU	4
Corals	*Acropora cerealis*	*Acropora cerealis*	EN	7
Corals	*Acropora chesterfieldensis*	*Acropora chesterfieldensis*	EN	3
Corals	*Acropora clathrata*	*Acropora clathrata*	EN	8
Corals	*Acropora cytherea*	*Acropora cytherea*	EN	10
Corals	*Acropora desalwii*	*Acropora desalwii*	VU	2
Corals	*Acropora digitifera*	*Acropora digitifera*	EN	11
Corals	*Acropora divaricata*	*Acropora divaricata*	EN	9
Corals	*Acropora donei*	*Acropora donei*	EN	2
Corals	*Acropora echinata*	*Acropora echinata*	EN	2
Corals	*Acropora elseyi*	*Acropora elseyi*	EN	4
Corals	*Acropora eurystoma*	*Acropora eurystoma*	EN	1

Corals	*Acropora gemmifera*	*Acropora gemmifera*	EN	8
Corals	*Acropora glauca*	*Acropora glauca*	EN	4
Corals	*Acropora globiceps*	*Acropora globiceps*	EN	8
Corals	*Acropora grandis*	*Acropora grandis*	EN	5
Corals	*Acropora granulosa*	*Acropora granulosa*	VU	4
Corals	*Acropora halmaherae*	*Acropora halmaherae*	EN	1
Corals	*Acropora hemprichii*	*Acropora hemprichii*	EN	4
Corals	*Acropora horrida*	*Acropora horrida*	EN	9
Corals	*Acropora insignis*	*Acropora insignis*	EN	3
Corals	*Acropora intermedia*	*Acropora intermedia*	EN	4
Corals	*Acropora irregularis*	*Acropora irregularis*	EN	1
Corals	*Acropora kimbeensis*	*Acropora kimbeensis*	EN	1
Corals	*Acropora kirstyae*	*Acropora kirstyae*	EN	1
Corals	*Acropora listeri*	*Acropora listeri*	EN	6
Corals	*Acropora lokani*	*Acropora lokani*	EN	1
Corals	*Acropora longicyathus*	*Acropora longicyathus*	EN	5
Corals	*Acropora loripes*	*Acropora loripes*	EN	8
Corals	*Acropora lovelli*	*Acropora lovelli*	EN	3
Corals	*Acropora lutkeni*	*Acropora lutkeni*	EN	6
Corals	*Acropora massawensis*	*Acropora massawensis*	EN	1
Corals	*Acropora microclados*	*Acropora microclados*	EN	6
Corals	*Acropora microphthalma*	*Acropora microphthalma*	EN	6
Corals	*Acropora millepora*	*Acropora millepora*	EN	4
Corals	*Acropora monticulosa*	*Acropora monticulosa*	EN	5
Corals	*Acropora mossambica*	*Acropora mossambica*	EN	1
Corals	*Acropora muricata*	*Acropora muricata*	EN	5
Corals	*Acropora nana*	*Acropora nana*	EN	5
Corals	*Acropora nasuta*	*Acropora nasuta*	EN	11
Corals	*Acropora nobilis*	*Acropora nobilis*	EN	3
Corals	*Acropora palmerae*	*Acropora palmerae*	EN	5
Corals	*Acropora paniculata*	*Acropora paniculata*	EN	4
Corals	*Acropora pinguis*	*Acropora pinguis*	EN	1
Corals	*Acropora polystoma*	*Acropora polystoma*	EN	8
Corals	*Acropora proximalis*	*Acropora proximalis*	EN	1
Corals	*Acropora pulchra*	*Acropora pulchra*	EN	5
Corals	*Acropora retusa*	*Acropora retusa*	EN	9
Corals	*Acropora robusta*	*Acropora robusta*	EN	9
Corals	*Acropora rongelapensis*	*Acropora rongelapensis*	EN	1
Corals	*Acropora rosaria*	*Acropora rosaria*	EN	5
Corals	*Acropora roseni*	*Acropora roseni*	VU	1

Corals	*Acropora samoensis*	*Acropora samoensis*	EN	5
Corals	*Acropora sarmentosa*	*Acropora sarmentosa*	EN	3
Corals	*Acropora secale*	*Acropora secale*	EN	8
Corals	*Acropora selago*	*Acropora selago*	EN	5
Corals	*Acropora solitaryensis*	*Acropora solitaryensis*	EN	4
Corals	*Acropora speciosa*	*Acropora speciosa*	VU	4
Corals	*Acropora spicifera*	*Acropora spicifera*	EN	4
Corals	*Acropora stoddarti*	*Acropora stoddarti*	EN	1
Corals	*Acropora striata*	*Acropora striata*	VU	4
Corals	*Acropora subglabra*	*Acropora subglabra*	EN	2
Corals	*Acropora subulata*	*Acropora subulata*	EN	5
Corals	*Acropora tenuis*	*Acropora tenuis*	EN	9
Corals	*Acropora turaki*	*Acropora turaki*	EN	1
Corals	*Acropora valenciennesi*	*Acropora valenciennesi*	EN	4
Corals	*Acropora valida*	*Acropora valida*	EN	11
Corals	*Acropora variabilis*	*Acropora variabilis*	EN	1
Corals	*Acropora variolosa*	*Acropora variolosa*	EN	1
Corals	*Acropora vaughani*	*Acropora vaughani*	EN	7
Corals	*Acropora verweyi*	*Acropora verweyi*	EN	4
Corals	*Acropora yongei*	*Acropora yongei*	EN	1
Corals	*Alveopora allingi*	*Alveopora allingi*	EN	4
Corals	*Alveopora daedalea*	*Alveopora daedalea*	EN	1
Corals	*Alveopora fenestrata*	*Alveopora fenestrata*	EN	2
Corals	*Alveopora marionensis*	*Alveopora marionensis*	EN	1
Corals	*Alveopora spongiosa*	*Alveopora spongiosa*	VU	2
Corals	*Alveopora verrilliana*	*Alveopora verrilliana*	EN	2
Corals	*Anacropora forbesi*	*Anacropora forbesi*	VU	4
Corals	*Anacropora matthai*	*Anacropora matthai*	EN	1
Corals	*Anacropora puertogalerae*	*Anacropora puertogalerae*	EN	1
Corals	*Anacropora spinosa*	*Anacropora spinosa*	EN	1
Corals	Artichoke coral	*Scolymia cubensis*	CR	6
Corals	*Astreopora cucullata*	*Astreopora cucullata*	EN	1
Corals	*Astreopora expansa*	*Astreopora expansa*	VU	2
Corals	*Astreopora listeri*	*Astreopora listeri*	EN	5
Corals	*Astreopora randalli*	*Astreopora randalli*	EN	2
Corals	*Astreopora suggesta*	*Astreopora suggesta*	EN	2
Corals	Atlantic mushroom coral	*Scolymia lacera*	CR	5
Corals	Birdsnest coral	*Seriatopora caliendrum*	VU	5
Corals	Bladed fire coral	*Millepora complanata*	CR	5
Corals	Boulder brain coral	*Colpophyllia natans*	VU	7

Corals	Boulder star coral	*Montastraea annularis*	EN	1
Corals	Boulder star coral	*Orbicella annularis*	EN	9
Corals	Brain coral	*Mussismilia harttii*	EN	1
Corals	Brain root coral	*Lobophyllia corymbosa*	VU	8
Corals	Branch coral	*Acropora florida*	EN	9
Corals	Branching fire coral	*Millepora alcicornis*	EN	9
Corals	Brush coral	*Acropora hyacinthus*	EN	10
Corals	Catch bowl coral	*Isopora palifera*	EN	11
Corals	Cauliflower coral	*Pocillopora damicornis*	EN	16
Corals	Cockscomb cup coral	*Desmophyllum dianthus*	EN	1
Corals	*Colpophyllia breviserialis*	*Colpophyllia breviserialis*	CR	3
Corals	Coral jaune	*Dendrophyllia cornigera*	EN	1
Corals	Crustal fire coral	*Millepora squarrosa*	CR	4
Corals	*Ctenactis albitentaculata*	*Ctenactis albitentaculata*	EN	2
Corals	*Ctenella chagius*	*Ctenella chagius*	CR	1
Corals	*Cycloseris curvata*	*Cycloseris curvata*	VU	1
Corals	*Cyphastrea agassizi*	*Cyphastrea agassizi*	VU	1
Corals	*Danafungia scruposa*	*Danafungia scruposa*	VU	3
Corals	*Dipsastraea laxa*	*Dipsastraea laxa*	EN	3
Corals	*Dipsastraea lizardensis*	*Dipsastraea lizardensis*	EN	2
Corals	*Echinopora lamellosa*	*Echinopora lamellosa*	VU	7
Corals	Elkhorn coral	*Acropora palmata*	CR	11
Corals	Elongate mushroom coral	*Pleuractis paumotensis*	EN	3
Corals	*Favites valenciennesi*	*Favites valenciennesi*	EN	1
Corals	Finger coral	*Acropora humilis*	EN	12
Corals	Fire coral	*Millepora dichotoma*	EN	3
Corals	Fire coral	*Millepora exaesa*	EN	4
Corals	Flower coral	*Eusmilia fastigiata*	CR	5
Corals	Grooved brain coral	*Diploria labyrinthiformis*	CR	7
Corals	Hump coral	*Porites attenuata*	EN	2
Corals	Intermediate valley coral	*Oulophyllia crispa*	EN	6
Corals	*Isopora brueggemanni*	*Isopora brueggemanni*	EN	4
Corals	*Isopora cuneata*	*Isopora cuneata*	EN	3
Corals	Lamarck's sheet coral	*Agaricia lamarcki*	CR	5
Corals	Largebrain root coral	*Lobophyllia hemprichii*	EN	11
Corals	Lettuce coral	*Agaricia agaricites*	VU	4
Corals	Lettuce coral	*Pectinia lactuca*	EN	4
Corals	*Lithophyllon concinna*	*Lithophyllon concinna*	EN	3
Corals	*Lithophyllon repanda*	*Lithophyllon repanda*	EN	3
Corals	Lobed cactus coral	*Lobophyllia diminuta*	EN	1

Corals	*Lobophyllia dentata*	*Lobophyllia dentata*	EN	1
Corals	*Lobophyllia robusta*	*Lobophyllia robusta*	VU	2
Corals	Low-ridge cactus coral	*Mycetophyllia danaana*	CR	2
Corals	Lowrelief lettuce coral	*Agaricia humilis*	CR	1
Corals	Massive starlet coral	*Siderastrea siderea*	CR	9
Corals	Maze coral	*Meandrina meandrites*	CR	7
Corals	*Millepora intricata*	*Millepora intricata*	EN	2
Corals	*Millepora nitida*	*Millepora nitida*	CR	1
Corals	*Millepora platyphylla*	*Millepora platyphylla*	EN	7
Corals	*Millepora tenera*	*Millepora tenera*	EN	2
Corals	*Montipora aequituberculata*	*Montipora aequituberculata*	EN	7
Corals	*Montipora altasepta*	*Montipora altasepta*	EN	1
Corals	*Montipora angulata*	*Montipora angulata*	EN	1
Corals	*Montipora australiensis*	*Montipora australiensis*	EN	2
Corals	*Montipora calcarea*	*Montipora calcarea*	EN	4
Corals	*Montipora caliculata*	*Montipora caliculata*	EN	9
Corals	*Montipora capitata*	*Montipora capitata*	EN	3
Corals	*Montipora capricornis*	*Montipora capricornis*	EN	1
Corals	*Montipora corbettensis*	*Montipora corbettensis*	EN	1
Corals	*Montipora crassituberculata*	*Montipora crassituberculata*	VU	1
Corals	*Montipora cryptus*	*Montipora cryptus*	EN	1
Corals	*Montipora danae*	*Montipora danae*	EN	5
Corals	*Montipora delicatula*	*Montipora delicatula*	EN	1
Corals	*Montipora digitata*	*Montipora digitata*	EN	6
Corals	*Montipora dilatata*	*Montipora dilatata*	VU	1
Corals	*Montipora efflorescens*	*Montipora efflorescens*	EN	9
Corals	*Montipora effusa*	*Montipora effusa*	EN	1
Corals	*Montipora floweri*	*Montipora floweri*	EN	3
Corals	*Montipora foliosa*	*Montipora foliosa*	EN	6
Corals	*Montipora foveolata*	*Montipora foveolata*	EN	8
Corals	*Montipora grisea*	*Montipora grisea*	EN	7
Corals	*Montipora hispida*	*Montipora hispida*	EN	7
Corals	*Montipora hodgsoni*	*Montipora hodgsoni*	EN	1
Corals	*Montipora hoffmeisteri*	*Montipora hoffmeisteri*	VU	7
Corals	*Montipora incrassata*	*Montipora incrassata*	EN	2
Corals	*Montipora informis*	*Montipora informis*	EN	7
Corals	*Montipora lobulata*	*Montipora lobulata*	VU	2
Corals	*Montipora maeandrina*	*Montipora maeandrina*	EN	1
Corals	*Montipora malampaya*	*Montipora malampaya*	EN	1

Corals	*Montipora millepora*	*Montipora millepora*	VU	2
Corals	*Montipora mollis*	*Montipora mollis*	EN	1
Corals	*Montipora monasteriata*	*Montipora monasteriata*	VU	8
Corals	*Montipora nodosa*	*Montipora nodosa*	EN	4
Corals	*Montipora orientalis*	*Montipora orientalis*	EN	1
Corals	*Montipora palawanensis*	*Montipora palawanensis*	EN	1
Corals	*Montipora peltiformis*	*Montipora peltiformis*	EN	1
Corals	*Montipora porites*	*Montipora porites*	EN	1
Corals	*Montipora samarensis*	*Montipora samarensis*	EN	1
Corals	*Montipora spongodes*	*Montipora spongodes*	EN	4
Corals	*Montipora stellata*	*Montipora stellata*	EN	3
Corals	*Montipora stilosa*	*Montipora stilosa*	EN	2
Corals	*Montipora tuberculosa*	*Montipora tuberculosa*	EN	10
Corals	*Montipora turgescens*	*Montipora turgescens*	EN	3
Corals	*Montipora turtlensis*	*Montipora turtlensis*	EN	2
Corals	*Montipora undata*	*Montipora undata*	EN	7
Corals	*Montipora venosa*	*Montipora venosa*	EN	8
Corals	*Montipora verrilli*	*Montipora verrilli*	VU	1
Corals	*Montipora verrucosa*	*Montipora verrucosa*	VU	9
Corals	*Montipora vietnamensis*	*Montipora vietnamensis*	EN	1
Corals	Mountainous star coral	*Orbicella faveolata*	EN	6
Corals	*Mussismilia hispida*	*Mussismilia hispida*	VU	1
Corals	*Pachyseris gemmae*	*Pachyseris gemmae*	VU	1
Corals	*Pavona cactus*	*Pavona cactus*	VU	6
Corals	*Pavona chiriquiensis*	*Pavona chiriquiensis*	VU	2
Corals	*Pavona duerdeni*	*Pavona duerdeni*	EN	5
Corals	*Pavona minuta*	*Pavona minuta*	VU	8
Corals	*Pectinia alcicornis*	*Pectinia alcicornis*	EN	3
Corals	Pillar coral	*Dendrogyra cylindrus*	CR	7
Corals	Pineapple coral	*Dichocoenia stokesii*	VU	4
Corals	*Pocillopora ankeli*	*Pocillopora ankeli*	EN	1
Corals	*Pocillopora capitata*	*Pocillopora capitata*	EN	2
Corals	*Pocillopora elegans*	*Pocillopora elegans*	EN	5
Corals	*Pocillopora fungiformis*	*Pocillopora fungiformis*	EN	2
Corals	*Pocillopora indiania*	*Pocillopora indiania*	EN	4
Corals	*Pocillopora inflata*	*Pocillopora inflata*	VU	1
Corals	*Pocillopora ligulata*	*Pocillopora ligulata*	EN	2
Corals	*Pocillopora meandrina*	*Pocillopora meandrina*	EN	11
Corals	*Pocillopora molokensis*	*Pocillopora molokensis*	VU	1
Corals	*Pocillopora verrucosa*	*Pocillopora verrucosa*	EN	17

Corals	*Pocillopora woodjonesi*	*Pocillopora woodjonesi*	EN	6
Corals	*Polycyathus isabela*	*Polycyathus isabela*	VU	1
Corals	*Porites evermanni*	*Porites evermanni*	EN	4
Corals	*Porites horizontalata*	*Porites horizontalata*	EN	2
Corals	*Porites lichen*	*Porites lichen*	VU	6
Corals	*Porites stephensoni*	*Porites stephensoni*	EN	2
Corals	*Porites superfusa*	*Porites superfusa*	EN	1
Corals	Rough cactus coral	*Mycetophyllia ferox*	CR	4
Corals	*Seriatopora aculeata*	*Seriatopora aculeata*	EN	3
Corals	*Seriatopora stellata*	*Seriatopora stellata*	EN	1
Corals	Staghorn coral	*Acropora cervicornis*	CR	11
Corals	Staghorn coral	*Acropora latistella*	EN	7
Corals	*Stylophora subseriata*	*Stylophora subseriata*	EN	1
Corals	Sunray lettuce coral	*Helioseris cucullata*	CR	2
Corals	Sunray lettuce coral	*Leptoseris cucullata*	CR	2
Corals	Symmetrical brain coral	*Diploria strigosa*	CR	1
Corals	Symmetrical brain coral	*Pseudodiploria strigosa*	CR	6
Corals	Ten-rayed star coral	*Madracis decactis*	CR	8
Corals	Thin birdsnest coral	*Seriatopora hystrix*	VU	7
Corals	Thin leaf lettuce coral	*Agaricia tenuifolia*	CR	3
Corals	*Tubastraea floreana*	*Tubastraea floreana*	CR	1
Corals	*Turbinaria reniformis*	*Turbinaria reniformis*	VU	4
Fish	American eel	*Anguilla rostrata*	EN	2
Fish	Ascension hawkfish	*Amblycirrhitus earnshawi*	EN	1
Fish	Ascension scorpionfish	*Scorpaena ascensionis*	EN	1
Fish	Atlantic Goliath grouper	*Epinephelus itajara*	VU	3
Fish	Atlantic horse mackerel	*Trachurus trachurus*	VU	1
Fish	Atlantic sturgeon	*Acipenser oxyrinchus*	VU	1
Fish	*Axoclinus cocoensis*	*Axoclinus cocoensis*	VU	1
Fish	Bigeye tuna	*Thunnus obesus*	VU	11
Fish	Black grouper (Gulf of Mexico pop.)	*Mycteroperca bonaci*	VU	2
Fish	Black musselcracker	*Cymatoceps nasutus*	VU	1
Fish	Black teatfish	*Holothuria nobilis*	EN	1
Fish	Blue marlin	*Makaira nigricans*	VU	3
Fish	Bluefish	*Pomatomus saltatrix*	VU	1
Fish	Brown-marbled grouper	*Epinephelus fuscoguttatus*	VU	9
Fish	Camouflage grouper	*Epinephelus polyphekadion*	VU	9
Fish	Cocos stargazer	*Gillellus chathamensis*	VU	1
Fish	Coelacanth	*Latimeria chalumnae*	CR	1
Fish	Cubera snapper	*Lutjanus cyanopterus*	VU	2

Fish	Dageraad	*Chrysoblephus cristiceps*	CR	2
Fish	Dusky grouper	*Mycteroperca marginatus*	VU	2
Fish	Dusty meagre	*Argyrosomus japonicus*	EN	1
Fish	False Bay klipfish	*Clinus latipennis*	EN	2
Fish	Gag	*Mycteroperca microlepis*	VU	3
Fish	Galápagos barnacle blenny	*Acanthemblemaria castroi*	VU	1
Fish	Giant seahorse	*Hippocampus ingens*	EN	1
Fish	Green Humphead parrotfish	*Bolbometopon muricatum*	VU	8
Fish	*Halichoeres adustus*	*Halichoeres adustus*	VU	1
Fish	*Halichoeres discolor*	*Halichoeres discolor*	VU	1
Fish	*Halichoeres salmofasciatus*	*Halichoeres salmofasciatus*	VU	1
Fish	Humphead wrasse	*Cheilinus undulatus*	EN	14
Fish	Lined seahorse	*Hippocampus erectus*	VU	2
Fish	Nassau grouper	*Epinephelus striatus*	CR	11
Fish	Ocean sunfish	*Mola mola*	VU	3
Fish	Priolepis ascensionis	*Priolepis ascensionis*	EN	1
Fish	Red grouper	*Epinephelus morio*	VU	2
Fish	Red steenbras	*Petrus rupestris*	EN	2
Fish	Red stumpnose seabream	*Chrysoblephus gibbiceps*	EN	2
Fish	Sailfin grouper	*Mycteroperca olfax*	EN	2
Fish	Sailfish	*Istiophorus platypterus*	VU	6
Fish	Scotsman seabream	*Polysteganus praeorbitalis*	VU	1
Fish	Seventy-four seabream	*Polysteganus undulosus*	CR	1
Fish	Shortjaw bonefish	*Albula glossodonta*	VU	3
Fish	Silver kob	*Argyrosomus inodorus*	VU	1
Fish	Sky emperer	*Lethrinus mahsena*	EN	3
Fish	Snowy grouper	*Hyporthodus niveatus*	VU	2
Fish	Squaretail coral grouper	*Plectropomus areolatus*	VU	6
Fish	St Helena sharpnose pufferfish	*Canthigaster sanctaehelenae*	EN	1
Fish	Tarpon	*Megalops atlanticus*	VU	4
Fish	Thorny seahorse	*Hippocampus hystrix*	VU	1
Fish	Whire salema	*Xenichthys agassizii*	VU	1
Fish	White steenbras	*Lithognathus lithognathus*	EN	2
Fish	White stumpnose	*Rhabdosargus globiceps*	VU	2
Fish	White-starred goby	*Chriolepis dialepta*	VU	1
Fish	Yellowfin grouper (Gulf of Mexico pop.)	*Mycteroperca venenosa*	EN	1
Fish	Yellowmouth grouper (Gulf of Mexico pop.)	*Mycteroperca interstitialis*	VU	2
Marine invertebrates	Edible sea cucumber (*)	*Holothuria scabra*	EN	1
Marine invertebrates	Giant clam (*)	*Tridacna gigas*	CR	1

Marine invertebrates	Hedgehog sea cucumber (*)	*Actinopyga echinites*	VU	2
Marine invertebrates	*Holothuria fuscogilva* (*)	*Holothuria fuscogilva*	VU	1
Marine invertebrates	Horse's Hoof Clam (*)	*Hippopus hippopus*	VU	2
Marine invertebrates	*Isostichopus fuscus* (*)	*Isostichopus fuscus*	EN	1
Marine invertebrates	Military sea cucumber (*)	*Actinopyga miliaris*	VU	1
Marine invertebrates	Perlemoen (*)	*Haliotis midae*	EN	2
Marine invertebrates	Pineapple seacucumber (*)	*Thelenota ananas*	EN	1
Marine invertebrates	Smooth giant clam (*)	*Tridacna derasa*	EN	1
Marine invertebrates	*Stichopus herrmanni* (*)	*Stichopus herrmanni*	EN	1
Marine invertebrates	White-belly sea cucumber (*)	*Actinopyga mauritiana*	VU	2
Marine mammals	Blue whale	*Balaenoptera musculus*	EN	6
Marine mammals	Dugong	*Dugong dugon*	VU	3
Marine mammals	Fin whale	*Balaenoptera physalus*	VU	3
Marine mammals	Florida manatee	*Trichechus manatus latirostris*	EN	5
Marine mammals	Galapagos fur seal	*Arctocephalus galapagoensis*	EN	1
Marine mammals	Galapagos sea lion	*Zalophus wollebaeki*	EN	2
Marine mammals	Humpback whale (Arabian Sea subpop.)	*Megaptera novaeangliae* (Arabian Sea subpop.)	EN	1
Marine mammals	Humpback whale (Oceania subpop.)	*Megaptera novaeangliae* (Oceania subpop.)	EN	1
Marine mammals	Indo-Pacific humpback dolphin	*Sousa chinensis*	VU	1
Marine mammals	North Atlantic right whale	*Eubalaena glacialis*	CR	1
Marine mammals	Sei whale	*Balaenoptera borealis*	EN	3
Marine mammals	Sperm whale	*Physeter macrocephalus*	VU	10
Marine mammals	West Indian manatee	*Trichechus manatus*	VU	2
Marine plants	South African eelgrass	*Zostera capensis*	VU	1
Marine reptiles	American crocodile	*Crocodylus acutus*	VU	1
Marine reptiles	Green sea turtle	*Chelonia mydas*	EN	49
Marine reptiles	Green sea turtle (E. Pacific subpop.)	*Chelonia mydas agassizii*	VU	3
Marine reptiles	Hawksbill sea turtle	*Eretmochelys imbricata*	CR	46
Marine reptiles	Kemp's ridley sea turtle	*Lepidochelys kempii*	CR	7
Marine reptiles	Leatherback sea turtle	*Dermochelys coriacea*	VU	25
Marine reptiles	Leatherback sea turtle (East Pacific Ocean subpop.)	*Dermochelys coriacea* (East Pacific Ocean subpop.)	CR	2
Marine reptiles	Leatherback sea turtle (Southwest Indian Ocean subpop.)	*Dermochelys coriacea* (Southwest Indian Ocean subpop.)	CR	3
Marine reptiles	Loggerhead sea turtle	*Caretta caretta*	VU	31
Marine reptiles	Marine iguana	*Amblyrhynchus cristatus*	VU	1
Marine reptiles	Olive ridley sea turtle	*Lepidochelys olivacea*	VU	10

Sharks and rays	Alfred's manta ray	*Manta alfredi*	VU	3
Sharks and rays	American cownose ray	*Rhinoptera bonasus*	VU	2
Sharks and rays	Atlantic chupare	*Styracura schmardae*	EN	3
Sharks and rays	Atlantic nurse shark	*Ginglymostoma cirratum*	VU	14
Sharks and rays	Atlantic pigmy devil ray	*Mobula hypostoma*	EN	4
Sharks and rays	Banded eagle ray	*Aetomylaeus nichofii*	VU	1
Sharks and rays	Basking shark	*Cetorhinus maximus*	EN	11
Sharks and rays	Bentfin devil ray	*Mobula thurstoni*	EN	14
Sharks and rays	Bigeye thresher	*Alopias superciliosus*	VU	17
Sharks and rays	Black-tip reef shark	*Carcharhinus melanopterus*	VU	27
Sharks and rays	Blackfin gulper shark	*Centrophorus isodon*	EN	1
Sharks and rays	Blacknose shark	*Carcharhinus acronotus*	EN	6
Sharks and rays	Blacktip shark	*Carcharhinus limbatus*	VU	16
Sharks and rays	Bleeker's whipray	*Pateobatis bleekeri*	EN	1
Sharks and rays	Blotched fantail ray	*Taeniurops meyeni*	VU	15
Sharks and rays	Bonnethead shark	*Sphyrna tiburo*	EN	5
Sharks and rays	Bottlenose wedgefish	*Rhynchobatus australiae*	CR	1
Sharks and rays	Bowmouth guitarfish	*Rhina ancylostomus*	CR	3
Sharks and rays	Bramble shark	*Echinorhinus brucus*	EN	2
Sharks and rays	Brazillian sharpnose shark	*Rhizoprionodon lalandii*	VU	1
Sharks and rays	Broad cowtail ray	*Pastinachus ater*	VU	3
Sharks and rays	Broadnose sevengill shark	*Notorynchus cepedianus*	VU	2
Sharks and rays	Brown stingray	*Dasyatis thetidis*	VU	1
Sharks and rays	Bull shark	*Carcharhinus leucas*	VU	18
Sharks and rays	Caribbean reef shark	*Carcharhinus perezi*	EN	8
Sharks and rays	Caribbean sharpnose shark	*Rhizoprionodon porosus*	VU	5
Sharks and rays	Coach whipray	*Himantura uarnak*	EN	6
Sharks and rays	Common eagle ray	*Myliobatis aquila*	CR	2
Sharks and rays	Common smoothhound	*Mustelus mustelus*	EN	1
Sharks and rays	Common thresher	*Alopias vulpinus*	VU	19
Sharks and rays	Copper shark	*Carcharhinus brachyurus*	VU	1
Sharks and rays	Duckbill eagle ray	*Aetomylaeus bovinus*	CR	1
Sharks and rays	Dusky shark	*Carcharhinus obscurus*	EN	4
Sharks and rays	Endeavour dogfish	*Centrophorus moluccensis*	VU	2
Sharks and rays	Equatorial skate	*Raja equatorialis*	VU	1
Sharks and rays	Great hammerhead	*Sphyrna mokarran*	CR	20
Sharks and rays	Grey reef shark	*Carcharhinus amblyrhynchos*	EN	21
Sharks and rays	Gulper shark	*Centrophorus granulosus*	EN	3
Sharks and rays	Happy Eddie	*Haploblepharus edwardsii*	EN	1

Sharks and rays	Harrisson's dogfish	*Centrophorus harrissoni*	VU	1
Sharks and rays	Indo-Pacific leopard shark	*Stegostoma tigrinum*	EN	7
Sharks and rays	Japanese shortnose spurdog	*Squalus megalops*	EN	1
Sharks and rays	Jenkins whipray	*Pateobatis jenkinsii*	EN	3
Sharks and rays	Kitefin shark	*Dalatias licha*	VU	1
Sharks and rays	Largetooth sawfish	*Pristis pristis*	CR	1
Sharks and rays	Leafscale Gulper Shark	*Centrophorus squamosus*	EN	3
Sharks and rays	Lemon shark	*Negaprion brevirostris*	VU	13
Sharks and rays	Leopard whipray	*Himantura leoparda*	EN	1
Sharks and rays	Lesser guitarfish	*Acroteriobatus annulatus*	VU	2
Sharks and rays	Little gulper shark	*Centrophorus uyato*	EN	2
Sharks and rays	Longfin mako	*Isurus paucus*	EN	13
Sharks and rays	Longhorned pygmy devil ray	*Mobula eregoodoo*	EN	1
Sharks and rays	Longsnout dogfish	*Deania quadrispinosa*	VU	1
Sharks and rays	Mangrove whipray	*Urogymnus granulatus*	EN	5
Sharks and rays	Marbled torpedo ray	*Torpedo marmorata*	VU	1
Sharks and rays	Monk's pigmy devil ray	*Mobula munkiana*	VU	1
Sharks and rays	Mosaic gulper shark	*Centrophorus tessellatus*	EN	1
Sharks and rays	Night shark	*Carcharhinus signatus*	EN	4
Sharks and rays	Oceanic mantra ray	*Mobula birostris*	EN	27
Sharks and rays	Oceanic whitetip shark	*Carcharhinus longimanus*	CR	22
Sharks and rays	Oman cownose ray	*Rhinoptera jayakari*	EN	1
Sharks and rays	Ornate eagle ray	*Aetomylaeus vespertilio*	CR	3
Sharks and rays	Pacific guitarfish	*Rhinobatos planiceps*	VU	1
Sharks and rays	Pacific sharpnose shark	*Rhizoprionodon longurio*	VU	1
Sharks and rays	Pelagic thresher	*Alopias pelagicus*	EN	11
Sharks and rays	Peruvian eagle ray	*Myliobatis peruvianus*	VU	1
Sharks and rays	Pink whipray	*Pateobatis fai*	VU	9
Sharks and rays	Porcupine ray	*Urogymnus asperrimus*	EN	8
Sharks and rays	Reef manta ray	*Mobula alfredi*	VU	16
Sharks and rays	Roughskin dogfish	*Centroscymnus owstonii*	VU	2
Sharks and rays	Roughtail stingray	*Bathytoshia centroura*	VU	1
Sharks and rays	Sandbar shark	*Carcharhinus plumbeus*	EN	11
Sharks and rays	Sandtiger shark	*Carcharias taurus*	CR	3
Sharks and rays	Scalloped hammerhead	*Sphyrna lewini*	CR	23
Sharks and rays	Scoophead shark	*Sphyrna media*	CR	1
Sharks and rays	Sharp-nose stingray	*Himantura gerrardi*	EN	1
Sharks and rays	Sharptooth lemon shark	*Negaprion acutidens*	EN	15
Sharks and rays	Shortfin mako shark	*Isurus oxyrinchus*	EN	17
Sharks and rays	Shortfin mako shark Indo-Pacific subpop.	*Isurus oxyrinchus* (Indo-Pacific subpop.)	VU	2

Sharks and rays	Shorthorned pygmy devil ray	*Mobula kuhlii*	EN	1
Sharks and rays	Shorttail nurse shark	*Pseudoginglymostoma brevicaudatum*	CR	1
Sharks and rays	Sicklefin devil ray	*Mobula tarapacana*	EN	17
Sharks and rays	Silky shark	*Carcharhinus falciformis*	VU	15
Sharks and rays	Silver chimaera	*Chimaera phantasma*	VU	1
Sharks and rays	Silvertip shark	*Carcharhinus albimarginatus*	VU	14
Sharks and rays	Smalleye hammerhead	*Sphyrna tudes*	CR	1
Sharks and rays	Smalltooth sandtiger	*Odontaspis ferox*	EN	7
Sharks and rays	Smalltooth sawfish	*Pristis pectinata*	CR	4
Sharks and rays	Smooth hammerhead	*Sphyrna zygaena*	VU	8
Sharks and rays	Snaggletooth shark	*Hemipristis elongata*	EN	1
Sharks and rays	Southern frillfin	*Bathygobius lineatus*	VU	1
Sharks and rays	Speckled smoothhound	*Mustelus mento*	CR	1
Sharks and rays	Spinetail devil ray	*Mobula japanica*	EN	2
Sharks and rays	Spinetail devil ray	*Mobula mobular*	EN	15
Sharks and rays	Spinner shark	*Carcharhinus brevipinna*	VU	4
Sharks and rays	Spiny butterfly ray	*Gymnura altavela*	EN	1
Sharks and rays	Spiny dogfish	*Squalus acanthias*	VU	1
Sharks and rays	Spotted eagle ray	*Aetobatus ocellatus*	EN	12
Sharks and rays	Spotted houndshark	*Triakis maculata*	CR	1
Sharks and rays	Tawny nurse shark	*Nebrius ferrugineus*	VU	16
Sharks and rays	Tiger catshark	*Halaelurus natalensis*	EN	1
Sharks and rays	Tope	*Galeorhinus galeus*	CR	1
Sharks and rays	Whale shark	*Rhincodon typus*	EN	29
Sharks and rays	White shark	*Carcharodon carcharias*	VU	21
Sharks and rays	White skate	*Rostroraja alba*	EN	1
Sharks and rays	White-tip reef shark	*Triaenodon obesus*	VU	27
Sharks and rays	Whitespotted eagle ray	*Aetobatus narinari*	EN	20
Sharks and rays	Whitespotted wedgefish	*Rhynchobatus djiddensis*	CR	4
Terrestrial invertebrates	Coconut crab (*)	*Birgus latro*	VU	6
Terrestrial reptiles	Giant tortoise (*)	*Geochelone gigantea*	VU	1

Table S4. Examples of observations of drifting fish aggregating device (dFAD) impacts on protected wildlife within marine protected areas (MPAs) based on survey and interviews. Two individuals (S-1 and S-2) left comments in the survey but were unavailable for an interview, all other content obtained from interviews (*n*=13 MPA affiliates from 17 MPAs and one shark sanctuary); participant identification code provided in brackets (INT-*n*). Affected species indicated in bold. See Table 1 for additional observations.

Impact	Species affected	Quote(s) explaining observed or inferred interaction
Entanglement (injury or mortality to protected species)	Sea turtles, marine mammals, fish, sharks, seabirds	"FADs pose a serious threat to the **leatherback and loggerhead turtles** that breed on the MPA beach. A couple of cases have been recorded of turtles caught up in ropes and buoys…[There was also] one case of a **shark** entangled in FAD rope and net." (S-1)<br><br>"In [MPA], there have been incidents of species entanglement in drifting FADs including **sea turtles**, **sharks** and other **fish**, as well as entanglement on rocky **reefs**." (INT-9)<br><br>"It's not uncommon to see **billfish** caught in FADs, **turtles** caught in FADs." (INT-11)<br><br>"On one occasion we found **a sea turtle** in the netting… it was famished, it was almost dead. And we brought it back and the park rangers gave it vitamins. They revitalized it." (INT-12)<br><br>"We quite often encounter FADs that have **turtles** entangled in them, and they have to be released. Quite often they are species of turtles that we don't see nesting on the beaches here as well. We have the hawksbill and green turtles that nest in [country], but there's five species of turtles that use [these] waters and the only time we really see these other species is when they are entangled in FADs. So, there's that issue of entanglement for turtles, there's bycatch of **sharks**, and other species as well. And then once they're attached to the reef, there's a lot of other species that get caught up in them too." (INT-13)<br><br>"We've seen that within [MPA], there will be a FAD caught [on the reef], and there's **fish** that have somehow got on top of the FAD and they're flipping around in this really shallow water." (INT-15)<br><br>"We haven't observed directly a whole FAD and **seabird** interaction, but we have definitely seen ropes and nets impacting them in the sense that they will try and get bits of them out for their nests. And sometimes what happens is they will get entangled somehow. We've got quite a few pictures of **boobies [*Sula* spp.]** kind of hanging by the ankles from trees and dying that way. There's no way of saying it's FAD-related but obviously most of that kind of material coming out of the ocean is fishing industry-related." (INT-15)

Entanglement (and physical destruction from collision)	Corals	"The large FADs rope gets entangled in the **reef** and rocks on the beach. They are also a hazard to small craft recreational fishing vessels in the area." (S-1)<br><br>"**Coral reef** habitat [has] very significant degradation due to entanglement of nets associated to the FADs." (S-2)<br><br>"We've had them [dFADs] wrapped around the **reef** sometimes because they have the tails, the netting. So, there's been times where we've cut them off the reef but most of the time they're not far from shore." (INT-7)<br><br>"We have one [dFAD] with netting that got stuck just outside the MPA, on the reef flat, outside the surf zone. It was about 50 meters down. That netting was stuck, and then it washed up and it pulled up all the **branching corals**. When the waves died down, and the float brought it up, it pulled up all the coral that could break off." (INT-16)<br><br>"This **reef**, it's close to a lot of people, and we'd find some net [from dFADs] every 300m on the reef." (INT-18)<br><br>"Once we found one that was offshore. I don't even remember how it was assembled with the floating stuff at the top, but it had this like 100-foot-long line of streamers attached to it. And that was entangled on everything, and it was hard stuck on the sea floor because of that…There are **seven species of coral that are considered threatened** that occur with varying degrees of regularity in [MPA]. Some are common, others are almost extinct. A major concern for us, with these things [dFADs] that entangle, is how they can very quickly wrap around something and shear it off. You lose the coral, and you lose the habitat, and it's kind of a trickle-down effect for everything that relies on the reef." (INT-8)<br><br>INT-10 described a large section of reef in the MPA composed of **coral** (specifically ***Acropora* spp**.) destroyed by a single dFAD collision<br><br>"They [dFADs] really do a number on the **reef** because these are big heavy nets, and they pretty much entangle the whole thing. You can see that the rafts pretty much bulldoze a swath through the reef." (INT-11)<br><br>"They [**deep sea corals**] are completely vulnerable to anything, even if it's just a physical destruction, something falls on them, they get entangled in something. Even if they were to break in and slowly regrow, you're talking about a couple hundred years to sort of see that damage mitigated." (INT-17)

		"What happens, usually, is the netting will get caught in the reef and the FAD structure will just stay at the surface. That netting will kill whatever [is underneath]. Because it's shadowing, there's no photosynthesis on that **coral.** But also, the smothering of it, the scratching, means that the polyps can't come out and feed as well." (INT-15)
Marine pollution and ingestion of plastics	Ecosystem	"Pollution due to electronic devices (buoys) [and] plastic pollution when they degrade." (S-2)<br><br>"They [dFAD buoys] are not always connected to something [when they strand], but many times they're connected to a plastic tabletop looking thing, sometimes it's a bamboo raft. There are some that are made out of PVC [polyvinyl chloride] and just green netting with nets underneath it. Sometimes it's a metal frame that's all rusted out and it'll have gray tubs that are empty for floating and black netting around that. There's all different kinds of ways that they put these things together." (INT-6)<br><br>"FADs also wash up on and pollute sensitive coastal sites home to **endangered species**, including **mangrove** habitats. Plastic netting and ropes linked with FADs quickly become brittle in these [environmental] conditions and can break down to microplastics, which then pose ingestion risk to wildlife." (INT-9)<br><br>INT-10 described observing changes in the materials and designs used over time. They perceived current models are often composed of metal frames, now sit lower in the water and have less netting material than in previous years. However, they noted new designs may also have more material overall.<br><br>"In addition to the different species that get caught up in FADs, there's issues of the materials that are used. Of course they're not biodegradable. There are ones now that are now supposedly biodegradable, but I would like to see a proper definition for 'biodegradable' because I personally wouldn't classify them as biodegradable. And all these materials are just being left out there in the ocean." (INT-13)<br><br>"The presence of that plastic, even if it's, you know, a big FAD on the shoreline, it's going to break down into smaller pieces that will then re-enter the coastline. And all of that is impacting the resilience of that ecosystem…Maybe it's not something that's so well thought about in terms of marine ecosystem resilience to climate change because it's more seen as like a coastline problem. But, of course, with islands, the two are just the same. There's not a barrier between the coastline and the ocean." (INT-15)

		"A lot of them [stranded dFADs] are sort of small pallets, rafts, plastic things. They were up on the coral beach, right up on the shore… it's obvious that if enough of those were washing up on the beach that some of them were going to get re-washed into the water and you'd have lithium-ion batteries breaking on reefs and into the deep-sea." (INT-17)
Disruption of nesting behaviour	Sea turtles	"We've got footage of where the beach crest is so covered in waste and most of it is big structures like the FADs and buoys and things. A lot of the **turtles** will come out [of the ocean] and then just turn around and go back down. So that's what we call an 'emergence stopped by obstacle' on beaches that have lots of waste…There's also [the situation that] if the turtle does get up, and finds somewhere to dig, the waste is so jampacked at every layer that they will just stop digging or they'll come across obstacles, or they'll find it difficult. So, they'll abandon digs. And, obviously, that's hugely exhausting energy-wise. And then, if they're successful, the turtle hatchlings have a real issue coming up through all of that. And then once they actually get up to the surface, they have to get to the ocean. The plastic pollution can prevent that from happening, but it can also mean they're exposed to predators for much longer because, you know, they're running the gauntlet for maybe hours rather than minutes, which would be the natural amount time." (INT-15)<br><br>"**Kemp's ridley**, the most endangered species of **sea turtle** in the world, these beaches [where dFADs strand] are the beaches that they're at. And if you go to any of the jetties or if you're in the surf anywhere at the beach, you're going to see **green sea turtles** all over the place, just tons of them." (INT-6)
Ecological trap†	Tuna, sharks, seabirds	INT-10 described that historically (>15 years ago), there were lots of tuna in MPA waters and tuna aggregating at dFADs were mostly adult yellowfin and skipjack. Today, the interviewee noted there are hardly any adult tuna around dFADs (mostly juveniles and other species, such **rainbow runner, wahoo, sharks, marlin, and triggerfish**). The interviewee perceived that changes could be linked to environmental factors but also noted that the introduction of dFADs likely changed **tuna** migration patterns, contributing to observed changes in species composition and size.<br><br>"I've had a lot of conversations with local elderly fishermen that have been fishing in [country] for the past 40, 50 years who have noticed significant changes with regards to specific **tuna**, but also **pelagic fish species** in general—where they catch them, where they don't catch them, and their size. That has drastically changed over the past few decades… basically what we are seeing now in [country] is caused by the impact that happened probably 10 or 20 years ago. The constant use of FADs and the overfishing aspect of it." (INT-14)

		"I think theory would dictate that they [dFADs] are going from productive areas to unproductive areas and disrupting migration patterns [of tuna] and things like that. But you would have to have a pretty high resolution of data to be able to make that statement observationally." (INT-11)

†Based on local ecological knowledge and observations over time, these interviewees speculated that changes to species distribution, behaviour, and/or body condition could be linked to the increased use of dFADs. However, all noted that there was a paucity of data and analyses to empirically confirm the degree to which these changes are due to increasing dFAD use relative to other environmental factors and threats.

Table S5. Observed challenges affecting drifting fish aggregating device (dFAD) retrieval. At-sea challenges reported by industry interviewees (*n*=5) and shore-based challenges reported by MPA-affiliated interviewees (*n*=15). Note: some retrieval by MPA staff also occurs at-sea but not in the open ocean (i.e. close to shore).

Interviewee	Challenge(s)	Quote(s)
INT-1	No specific challenges associated with retrieval mentioned	
INT-2	De-activation (cost)	"Our fishing company has to pay a daily fee for the beeping [GPS buoy transmitting]. But if you lose it [the dFAD], you don't want to pay any more."
INT-3	De-activation (cost), logistical (retrieval distance), seize/weight), waste management	"Typically, the model has been—across the world—that the FADs will drift, and they [fishing vessel operators] will keep tracking them until they drift out of the feasible area where vessels will fish. Then they turn off the satellite buoys because then you don't have the cost of transmission. But we don't know where they end up after that."<br><br>"Because their drifting trajectories can be so far, it's uneconomic to retrieve them. It's difficult to find them. Even though they've got a tracking device on them, they're not necessarily accurate [and] you can be searching around, looking for something to retrieve but it's literally thousands of kilometers away from where the boats are fishing."<br><br>"The other thing too, with retrieval, is that it's very difficult. If they [dFADs] still have their tail attached and the raft, they're extremely heavy and to lift them out of the water, you need a boat that's large enough, sturdy enough, with a little crane to pull them out. And I actually don't know how they're doing in [MPA]. They must be dragging them back to near shore and then breaking them down in the sea or something…You also need waste management systems, and to break them down and figure out what you're going to do with all the different components that you've pulled in."
INT-4	De-activation (regulations, cost), logistical (retrieval distance)	"When [RFMO] regulations changed, and they limited the number of active FADs [per vessel], that started incentivizing people to deactivate the buoys as soon as they went outside of the main fishing zone [because] even though they know that sometimes their FADs are going to come back, they can't keep them active and wait."<br><br>"For [fishing] companies that are not near them [active dFAD limits], cost is a much bigger factor.

		For them, once the FAD is completely out of reach, they will de-activate.”
INT-5	De-activation (cost), logistical (seize/weight),	**“**Right now, if they [fishing company] can’t use them, there's a cost for the [buoy] communications. And nobody wants to pay. So, the tendency is they are de-activated.”<br><br>“The problem [retrieving at-sea] is sometimes they [the dFADs] are really heavy.”
INT-6	Logistical (remote location, limited human capacity)	“They [MPA staff] always want me to bring them back [but] I don't have the capacity to do that, you know? But I do take as many photos as possible.”<br><br>“Nobody removes the FADs from [MPA]. You know, there's no access over there. There are no roads to get there.”
INT-7	No specific challenges associated with retrieval mentioned	
INT-8	Logistical (limited human capacity, dFAD size/weight), financial	**“**So, it [removal of stranded dFADs] falls on the park, much like all the other marine debris that washes ashore. I have a team right now—myself and nine other individuals—who supervise, and we conduct beach cleanups and reef cleanups. So, as we encounter these things, we pick them up. During the winter, we utilize a volunteer program. Every time we go out and do a beach cleanup, we take anywhere between four and 12 volunteers with us. And so sometimes they're the ones helping us pick up these big bulky rafts to get them off [the beach]. And for disposal, it’s with all the other trash. The park spends about $2,500 a month on a dumpster that we use to collect all the marine debris that we pick up. That’s not our office trash and park business—it’s the stuff that we’re picking up that has washed ashore.”
INT-9	Logistical (limited human capacity, remote location, dFAD size/weight), financial, waste management, safety	“At the moment, recovery of FADs [by MPA staff] is just when there's the opportunity, if the funding can be raised for the boat time quick enough, and if they are reported… A big focus for us right now is to try and make that reporting better but in terms of the [retrieval] responsibility, it’s a shame that it has to be on a very resource-limited authority like [MPA staff] because they’re stretched already and it's a big burden.”

		“In an island system like [MPA], where waste management is a huge issue and the landfill is under massive pressure, the [retrieved] FADs add a huge amount of pressure to that because one FAD weighs so much and is so big…that's just another factor to keep in mind in terms of the burden that it puts on local resources.”<br><br>**“**I know that recently, FADs had to be recovered because their tails were stuck [on reefs], which in itself a really big hazard… I'm sure it is in other places as well, but the currents [in MPA] are strong, it’s very deep, it costs a lot of money to get to the remote sites and obviously, they are incredibly heavy things.”
INT-10	Logistical (limited human capacity)	INT-10 discussed how dFADs are observed so frequently in MPA waters that MPA staff must focus efforts only on those most likely to strand. INT-10 also discussed that MPA staff are currently responsible for disposing of all dFAD debris retrieved.
INT-11	Logistical (limited human capacity)	“It's usually local communities, local natural resource management agencies [that retrieve stranded dFADs]. In [MPA #1], it’s the [local NGO] staff. It’s not a huge atoll in [MPA #2]. What usually happens [there] is if one is readily observable, the community will call [local NGO] and they'll try to get it off the reef.”
INT-12	Logistical (remote location), waste management	“They [dFADs] get immediately removed by [MPA staff] as soon as they get notice that there's one around. And since there's always dive operators around there, whenever a dive operator sees one, they report it… For years they would just pile it all up, all the decommissioned [fishing] gear. Now they have this big warehouse with hooks and lines and gillnets and FADs. I know that a couple of years ago, they made some kind of deal with an NGO, and they brought one of the dive boats [to MPA] exclusively to remove all the trash.”
INT-13	Logistical (remote location, limited human capacity), financial, waste management	“There's a huge cost involved in terms of taking boats out there to [remote MPA], collecting them [dFADs] from the reef, and bringing them all the way back to [main inhabited island]. It’s very time consuming and expensive, and we lack local capacity in terms of vessels and personnel so it's a huge undertaking.”

		“We've got a huge problem in [country capital] that our landfill is full. And it's been full for a number of years, and we don't have a solution… the majority of the waste that goes in there is fishing gear. So even if we remove the FADs from the reef or the beach, and we bring them back to [capital city], there's nobody recycling [them] locally. So, they get dumped in the landfill and that's a massive problem as well.”
INT-14	De-activation (abandonment), logistical (remote location, limited human capacity)	“There is no specific arrangement of a recovery process in [country]…I’ve met with fishing captains [and they’ve said] there is close to zero recovery happening on their side. So, what that's saying is that all of the dFADs deployed within [country EEZ] are basically left to be stranded.”<br><br>“The majority of the dFADs that do wash up or are stranded [in country] happen out of sight of humans…There are cleanups [but] they’re very isolated, limited, specifically done by [local NGO]. At [MPA] they are done by [NGO]. And these are more of an opportunistic kind of cleanup or recovery. There is no specific arrangement or formalization of a recovery process in [country].”
INT-15	Logistical (human capacity, dFAD size/weight), financial	“It's a huge issue for [country], especially for [remote MPAs] because it costs so much money to do waste removal just to get it back to the mainland where there's landfill and it's definitely not cost effective. It's not sustainable to do it that way. And obviously, FADs make up just huge volume and weight. Just one FAD is a lot.”
INT-16	Logistical (human capacity), financial	“It [a dFAD] got stuck down at 50 meters off the drop and then washed up to the shelf, to the surf area. And it did take a lot of effort to take that thing off the reef. Manpower, scuba gear, air tanks, time, you know? To lift and then cut, making sure you do not break it off the coral, but cut it off.”<br><br>“At the moment, there's no [retrieval] program in [MPA or country]… there's no offshore program that is doing a daily or weekly cleanup because of the lack of funding.”
INT-17	Logistical (human capacity, remote location)	“In collaboration with the [NGO], we cleared a section of beach on one of the uninhabited islands [in MPA] and came back a year later to see how many had washed up… It was something on the order of like 30 FADs washed up within a tiny little

		section of beach…[We don't] know if the spot we cleared, for whatever reason, just happens to be an accumulation site, but it was astoundingly high."<br><br>"[MPA] is an uninhabited archipelago with the exception of one island. So, there aren't people on the ground looking. Every time we go, we find some, but we don't often clear them…"
INT-18	Logistical (human capacity, remote location, size/weight), safety	"It's quite hard to put them [dFADs] onto the boat. They're heavy, they're full of animals [from biofouling], they're dirty."<br><br>"There are many sharks, and when you want to retrieve FADs, they are around and they are not used to seeing divers, or tourists in general, because we were really [in places] that are very exposed to the wind and the waves. So, they see you and they will charge you. They're not that aggressive, but they're extremely curious… They [the dFADs] come with the current, then they will get stuck on the reefs were pretty much no one ever goes. So, you'll find this quite a lot."

Table S6. Existing and proposed solutions to address drifting fish aggregating device (dFAD) impacts on marine protected areas (MPAs) and the marine ecosystem. Note: Regional Fisheries Management Organization (RFMO) regulations current as of April 2025; content summarized from interview data with additional reference to examples from the literature, see table footnotes for example quotes ('Q*n*'). Acronyms: EEZ = Exclusive Economic Zone (i.e. a country's sovereign marine territory extending 200 nautical miles offshore), MSC = Marine Stewardship Council, ISSF = International Seafood Sustainability Foundation, IATTC= Inter-American Tropical Tuna Commission (E. Pacific Ocean RFMO), ICCAT = International Commission for the Conservation of Atlantic Tunas (Atlantic Ocean RFMO), IOTC = Indian Ocean Tuna Commission (Indian Ocean RFMO), WCPFC = Western and Central Pacific Fisheries Commission (W. Pacific Ocean RFMO), PNA = Parties to the Nauru Agreement (see footnote), GPS = Global Positioning System.

Impact reduction approach	Solution	Existing RFMO regulations	Existing industry efforts and perspectives	Existing MPA efforts and perspectives
Limit interaction	Deployment limits	No RFMO has regulations that specifically cap the number of deployments a vessel may make each year, nor the cumulative number of deployments allowed by all vessels in an ocean. However, IOTC limits how many new dFADs a vessel may acquire annually (i.e. 400), and all RFMOs have regulations limiting the number of active (i.e. GPS transmitting) dFADs a vessel may have at one time (~300, depending on the RFMO). This means that when a dFAD drifts out of range, it may be de-activated remotely by the fishing company (without retrieving it) and a new active dFAD can be deployed to replace it.	Most industry interviewees noted fishing companies already use fewer active dFADs than RFMO regulations permit but active limits can also incentivize de-activation for some companies operating at the limit (see 'Challenges' in table S5). Some interviewees expressed concern over the number of dFADs currently used (e.g. Q1) or suggested more regulation to limit deployment was needed (e.g. Q2, Q3). Equally, others noted that purse seine companies have become dependent on dFADs and that limiting their use could affect business outcomes (e.g. Q4, Q5).	Not applicable

Limit interaction (continued)	Spatial management of deployments*	No RFMO has regulations that restrict dFAD deployments in areas that lead to high rates of drift through MPAs, nor in areas that lead to high stranding incidence (in MPAs or elsewhere).<br><br>ICCAT, IATTC, and WCPFC all have annual spatial-temporal closures for purse seine fleets using dFADs, but these measures were designed to limit total fishing effort and closure areas were determined based on benefits to target tuna stocks rather than to limit dFAD impacts or losses. Companies are not allowed to deploy dFADs in the weeks leading up to the closure. One interviewee suggested longer spatial-temporal closures would also reduce dFAD interactions (coded as part of 'Other' in Figure 4).	All industry interviewees discussed how vessel captains use experiential knowledge and software programs to help predict dFAD trajectories (e.g. Q6, Q7). Interviewees further noted that captains must be at least 30 nautical miles from land when they deploy, and most will not deploy dFADs in areas where they are likely to get lost or stranded due to local environmental conditions (e.g. Q8).	Some MPA staff have conducted research to better understand the spatial relationship between dFADs and MPAs based on local environmental conditions but measures to limit interaction spatially must be developed domestically or through RFMOs. Marine spatial planning initiatives and associated regulations have been established by some countries where dFAD fishing is permitted to limit interaction with local MPAs (e.g. *89*) but the degree to which domestic zoning for fishing vessels also minimizes strandings may be limited (e.g. Q9).
Limit interaction (continued)	Limit buoy de-activation & ensure at sea retrieval	As of 2024, IOTC has prohibited the deliberate discarding (abandonment) of dFADs except in cases of *force majeure* and stipulates that dFAD retrieval must include both buoy and raft (Res. 24/02). In all other RFMOs, vessels are required to retrieve active dFADs in the weeks prior to annual spatial-temporal closures and most have regulations discouraging de-activation and otherwise encouraging (but not requiring) the retrieval of dFADs at-sea. There are no limits at any RFMO stipulating the total number of dFADs that may be de-activated each year by a single vessel or by the entire purse seine fleet.	Many interviewees highlighted that buoy de-activation and abandonment are key challenges that have not been sufficiently addressed (see 'Challenges' in table S5). To improve retrieval rates, some interviewees suggested modifying existing RFMO regulations to allow support vessels or other fishing vessels to collect dFADs (e.g. Q10). To reduce de-activation, one other interviewee suggested offering discounted GPS transmission costs for buoys drifting out of fishing grounds. Some industry interviewees noted that the retrieval of a dFAD at-sea (even when not catching tuna) is legally an act of "fishing" and therefore, it can only occur in places where dFAD fishing is permitted (i.e. not during a spatial-temporal closure, in MPAs, or in EEZs without access agreements). One interviewee also mentioned that there have been discussions about an	Not applicable

			industry-funded dFAD recovery program (e.g. Q11).	
	Shared dFAD buoys	None	Currently, dFAD buoy data are proprietary and this information is mostly shared among vessels of the same company. In the Indian Ocean, the sharing of dFAD buoy data across fleets is one approach used to minimize losses (*103*). One interviewee suggested that sharing dFADs across the entire purse seine fleet in each ocean could reduce the number of devices at sea. At the same time, this would also eliminate any competitive advantage companies think they have, which may make it an undesirable approach.	Not applicable
Minimize damage	Non-entangling designs	As of 2025, all RFMOs now require fleets to use non-entangling dFAD designs and prohibit the use of mesh netting on devices. The prohibition of netting is important since non-entangling designs did not automatically exclude mesh but rather attempted to reduce its potential for harm (e.g. by rolling it like a sausage). Previously, rolls could unravel and become entangled on reefs (see Fig. 1B). Currently, adopted regulations related to non-entangling designs do not preclude the use of rope, canvas or cloth.	Interviewees noted a large shift in the industry toward non-entangling designs in the last decade and this is now seen as common practice for most fleets, especially given RFMO requirements and voluntary best practice guidelines (e.g. Q12). At the same time, a lack of RFMO standards for onboard observers to audit these new designs makes it challenging for companies to address MSC conditions and demonstrate improvements have been made (e.g. Q13).	Not applicable
	Biodegradable materials†	All RFMOs encourage the use of biodegradable materials when designing dFADs. The adoption of fully biodegradable designs is set for 2028 at ICCAT (Rec. 24-01) and 2029 at IOTC (Res. 24/02), but no other RFMO has set an explicit date for complete elimination of non-biodegradable designs. In 2026, WPCFC members will decide on the implementation of biodegradable dFAD requirements (CMM 2023-01) and, by 2030, IATTC members must decide whether they will fully	Many companies now exceed RFMO biodegradable minimum requirements. All industry interviewees noted that MSC-certification and engagement with ISSF were influential in pushing best practices in design (see table S7). One outstanding challenge with some biodegradable designs is ensuring their durability at-sea (e.g. Q14) and a reliable supply of materials (e.g. Q15).	Not applicable

Minimize damage (continued)		phase-out all non-biodegradable materials (C-23-04).		
	Weight limit	None. Beyond regulations related to non-entangling and biodegradable materials (above), there are no other RFMO regulations specifying dFAD construction requirements.	No industry interviewees mentioned existing efforts to reduce the weight of their dFAD rafts, but discussion related to this topic has begun (e.g. Q16) and several interviewees remarked on the difficulties associated with dFAD retrieval due to weight (see 'Challenges' in table S5). Models such as the 'Jelly FAD' have been developed to minimize weight and incorporate biodegradable materials (*77)*, but some industry members expressed concerns over this model's robustness and likeability (e.g. Q14, Q17), which suggests additional improvements to its design may be needed.	Not applicable
	Sinking sensor		Certain buoy models now contain sensors to alert crew when a dFAD is at risk of sinking (e.g. Satlink SLX+ 'ECOBuoy'). These sensors may be especially useful for biodegradable dFADs, which have historically been less reliable in rough oceanic conditions than non-biodegradable designs. Ultimately, their value depends on whether a vessel retrieves the compromised device in time.	Not applicable
	Re-usable dFADs		One interviewee suggested transitioning toward dFAD rafts that are designed to be re-usable would be one approach to limit debris and waste (assuming retrieval rates increase in parallel). Currently, some buoy companies are working to make re-usable designs cost-effective and available at-scale (e.g. the Zunibal Zunfloat).	Not applicable

	Beach cleanups	Not applicable	None	All MPA interviewees discussed efforts to remove dFAD debris from MPA coasts and reefs (see table S5). Some cleanups have been supported by local NGOs or government funding but most MPAs lacked a structured approach and were only able to conduct cleanups when resources and time allowed. In addition to capacity constraints, beach cleanups are not always possible in remote locations and even identifying sites in need of dFAD removal is challenging in MPAs with uninhabited islands (see 'Challenges' in table S5).
	Shore-based at-sea retrieval	Not applicable. Since the retrieval of a dFAD (even if there are no fish associated with it) is considered "fishing", a vessel can only legally retrieve a dFAD within a EEZ if it is licensed to fish in that EEZ (domestically or through an access agreement). Further, since industrial fishing cannot occur near-shore (i.e. usually within 30 nautical miles), RFMO regulations cannot mandate near-shore retrieval. However, the IOTC does require vessel operators to notify local authorities when a dFAD has been de-activated in their EEZ.	Recently developed. Several interviewees mentioned multi-stakeholder retrieval programs that seek to collect dFADs within a few kilometers of shore before they strand (*78, 104*) and discussed the potential for scaling up such efforts (e.g. Q18). Such 'FAD Watch' programs have been implemented in the Seychelles and Palmyra Atoll and there has been discussion on expanding to the Galápagos Marine Reserve (*79*). Since these multi-stakeholder initiatives depend on both industry cooperation as well as local capacity (e.g. Q19) they are likely most beneficial in small areas with high stranding densities and are likely not a viable solution for large EEZs, remote islands, or places with limited local capacity to assist in retrieval and dispose of debris, and multiple interviewees noted that the scale of shore-based recovery efforts is currently insufficient.	
Ensure accountability	Standardized labelling & buoy traceability	In progress. All RFMOs require that each company's dFAD buoys are labelled with a unique identifier, but approaches are not yet standardized and do not also include labelling requirements for dFAD rafts (or other parts). The only RFMO to establish a buoy registry is the IOTC, which will be implemented in 2026 (Res. 24/02). This registry will not be public-facing and currently there is no way to identify and trace	Multiple industry members acknowledged the need for improved accountability, and the importance of traceability (e.g. Q20). Many MPA interviewees noted stranded dFAD buoys were labelled with at least some identifying information (e.g. Q21) and multiple interviewees highlighted that buoys from US-flagged vessels were among the easiest to identify. However, labelling on buoys was of limited use for	Although not specific to MPAs, countries that license dFAD purse seine vessels in their EEZs can implement dFAD registry requirements. For example, all companies fishing in the EEZs of eight Pacific Island states (i.e. the PNA‡) must register their dFAD buoys as part of their access agreement (*105*).

		all parts of an abandoned or stranded dFAD back to its owner.	knowing how to contact buoy owners (e.g. Q22).	
	Polluter-pays model	None	There is no comprehensive program to account for all damages associated with dFAD strandings or losses. Some interviewees discussed regional efforts to reimburse fishers for returning stranded dFAD buoys (e.g. Q23) but these are location specific. One interviewee discussed the potential of an upfront environmental fee associated with the purchase of each dFAD buoy that could be returned only if the buoy is retrieved.	Some countries require purse seine companies to pay an environmental fee as part of their fishing access agreement, but some MPA interviewees expressed concerns over existing amounts (e.g. Q24) and the need for stronger financial deterrents to dFAD abandonment (e.g. Q25). Currently, this approach only applies to countries where dFAD fishing occurs and does not account for losses or damages occurring elsewhere. One industry interviewee acknowledged that the PNA will likely implement a polluter-pays strategy in the future as part of their fishing access agreements (Q26).
	Clear ownership rules & responsibilities	In conjunction with the buoy registry, IOTC has provided definitions of dFAD ownership and associated responsibilities for dFAD reporting and transfer of ownership (Res 24/02). No other RFMOs have operational definitions or explicit requirements for dFAD owners.	None	Domestic approaches are limited to places where dFAD fishing is licensed. The PNA have definitions and requirements related to dFAD ownership as part of their access agreement and dFAD registry (*105)* and any country that authorizes dFAD fishing in its waters could apply a similar strategy.
	Buoy re-purposing programs	Not applicable	Multiple interviewees mentioned Project Recon, a multi-stakeholder initiative led by the buoy provider Satlink to re-purpose stranded buoys for local use by small-scale fishers or for conservation purposes (*106*). As with near-shore retrievals, the scope and effectiveness of these initiatives depends on establishing connections with local stakeholders. Further, since buoys are only one part	Multiple MPA Interviewees discussed how communities adjacent to MPAs will sometimes take or modify dFAD buoys or raft debris for personal use (e.g. Q27, Q28).

			of dFADs, additional effort is still needed to collect and re-purpose other dFAD parts.	

Q1: "In my opinion it [the current active dFAD limit] is too much…it [the ocean] is going to be a carpet. I don't know based on what they [RFMO member states] took that decision. However, at least there is something." (INT-2)

Q2: I think we need to transition that way [toward ownership rules] really fast because if we don't, if we don't pressure the authorities to regulate this as fast as possible, we're going to get FAD bans…It [dFAD traceability] is something that gives more control than just deploying and deploying and deploying. Even though they're biodegradable FADs, there are companies deploying many, many FADs." (INT-1)

Q3: "I would say it's kind of where we need to go, to actually reducing the amount in the water in general…if you had a quota system on how many deployments you can make based on historic data or something like that, well, maybe that would be that would be an approach." (INT-4)

Q4: "Purse seiners, the way they are now compared to the 1980s is significantly different. Right now, they cannot operate without dFADs. Their whole operation would go down because their boats [now] are bigger, slower, and they have the whole deck—the whole system—decked out specifically for dFAD fishing." (INT-9)

Q5: "The primary concern for any purse seine vessel owner, is any regulatory stuff, anything around FADs. Because you can't sustain a purse seine fishing business by fishing on free schools alone. You need to be able to deploy FADs." (INT-3)

Q6: **"**With the buoy, you get access to software that you can use to see where it [the dFAD] is… in that same software is also integrated oceanographic information—temperature maps, chlorophyl maps, but also ocean current maps. Which are super useful for not just figuring out where they [fishers] can set their nets safely, but also to be able to understand where their FADs are going to go and what current they are going to move with. Within the platform, they also have tools to predict the trajectory, the future trajectory of the of the group based on both the past trajectory of the boat and current data." (INT-4)

Q7: **"**They [captains] use a program called CatSat. Well, they have it. It predicts currents and all this stuff. But I don't think they're using that information as analytically as we would like to think to go, 'okay, if I deployed here at this time on this date, it's likely going to end up there'. For them a lot of it is gut feel, it's still intuition, it's based on previous knowledge." (INT-3)

Q8:"The top reason why they [captains] don't want to deploy FADs there [area with high current] is because as soon as you put it in, it doesn't have time for harvesting, it goes straight to the beach." (INT-2)

Q9: "It [country EEZ] has basically been split into different zones, one of which is no-take where no fishing allowed at all. So, purse seiners aren't allowed in there…Of course, if they use drifting FADs, it doesn't mean the FADs aren't going into the no-take zone…the number of FADs you find on the beach and on the reef [in no-take MPA] is just incredible. It's been a problem for a long time now. *"* (INT-13)

Q10: "How can we do that [shore-based recovery of dFADs] wider? It's a logistics problem, but there are a lot of people in local industries or local situations that want to do that. If there was a cost-effective way that the FAD can recovered before they goes too far away from the fishing area, that could be interesting. Let's say each fishing company is able to use their supply vessels—if they have any—to recover the FADs, bring them back and redeploy them within the fishing area. Therefore, they're always used and always being monitored and never get to the coast." (INT-5)

Q11: **"**All the companies are interested, they want to help fix the problem, but they don't have an at-hand solution. So, for example, they're talking about a foundation. Nobody's made a foundation yet. Who's going to pay the money? How does it work? Who's going to open the bank account? Who's going to sign the checks? …And once you get a bank account and everybody throws money in the bank account, where do you hire the vessels? And do you buy one? Do you lease them? …Where do you start?" (INT-5)

Q12: "With the transition to non-entangling FADs, and those requirements becoming binding now throughout RFMOs, the issue about bycatch entanglement has gone. The issue of sharks being entangled, or turtles, that's less of a priority now because there's a solution in place. So now it's moving on to FAD recovery and bio[degradable] FADs. From an environmental perspective, that's the plan for sure." (INT-3)

Q13: "The vessels have a responsibility for all their future deployments to be compliant [once a RFMO measure comes into force], but then it'll take a couple of years for the [RFMO] Compliance and Monitoring process to catch up. And we're seeing that with the non-entangling FADs. And it's a problem in the MSC certifications where, for example, we're using non-entangling designs, but we can't demonstrate that. We have no external verification of because [RFMO] observers aren't compliance

monitoring it yet. But our [MSC] condition says, 'you need to provide external validation that your designs are non-entangling'. And we're like, 'how can we if the system hasn't caught up yet?'" (INT-3)

Q14: "They [fishing captains] have seen that with the biodegradable and non-entangling jelly FAD that it's too weak [to handle currents in fishing area]." (INT-2)

Q15: "I think we're up to something like nearly 80% of the FAD is biodegradable. The [country] fleet has a program in place and they're looking at trying to get—across the fleet—a benchmark of 50% biodegradability within a certain time frame. So, it's possible. Our biggest issue has actually been securing a reliable industrial-scale supply of the materials that you need." (INT-3)

Q16: **"**One of the things that [is being] discussed is to make a best practice to control their weight because physically pulling them [dFADs] out of the water is a big problem. Let's say you develop more [retrieval programs] around the world with stakeholders of the local environment, and they want to prevent damage on their reefs. If physically they can't pull them out of the water, it's really hard." (INT-5)

Q17: **"**We've been involved in trials for that [Jelly FADs] but our fishing masters don't like the designs." (INT-3)

Q18: **"**I think the biggest thing [to ensure low impacts] is to develop more what are called 'FAD Watch' programs. And there's samples around the world. But what they do is a drop in the bucket. But it proves they work." (INT-5)

Q19: "What you require for that [near-shore recovery] is that the vessel owners need to voluntarily participate, and you need the cooperation of the bouy provider to track the data and inform. And then you need an entity, or someone the entity has subcontracted, to be the collector of the of the FAD. And then you need a solution for what to do with the waste from that after it's been recovered. So there are a lot of MPAs that don't have that kind of program.*"* (INT-3)

Q20: **"**I sincerely think that companies have to be responsible for what they put in the water. If you put waste in the water, you should be responsible for it and you should be traceable. This waste has to be traceable to the vessel." (INT-1)

Q21: "Many of them [stranded dFAD buoys] don't have anything [labelled on them]. But then you can see signs also [on some], sometimes letters, sometimes names… So then what does it mean? We don't know. It's hard to, you know, draw any conclusion behind it, but for sure there are things written on them. But the thing that's interesting is that you see they have like Spanish [vessel] names and stuff and their [buoy] brand will be the same, their serial numbers will be close." (INT-18)

Q22: "Most of them [dFAD buoys] still have the number on them. A lot of them have the number melted into the top part of them. And then the I guess they have a country code, which is a 2 to 4 letter code, and then some numbers afterwards, which I guess identifies who the fisherman is? I have no idea." (INT-6)

Q23: "If it's to the point that the industry is willing to pay for retrieval of buoys, that's a significant indicator that this isn't just one or two [showing up]…There was someone in [MPA] who would buy back FADs [from locals], at least the transponders, and send them back [to the companies]." (INT-7)

Q24"Under their [fishing companies'] licensing conditions, they do pay an environmental fee. And some of them are under the assumption that they wipe their hands clean once they've paid a fee and signed a contract…it is such a minimal fee that it won't even fund one [dFAD] recovery trip." (INT-14)

Q25: "At the moment it feels like they [fishing companies] just get away with whatever they, you know, they get away with everything. And I think it's really important that we start calling this out properly and that there is some mechanism in place within [country], that there isn't just this damaging with impunity of ecosystems that are so valuable. The price tag attached to one FAD, you know, destroying an area of coral reef or a coastline that's kind of priceless but it [the mindset] is 'oh well'…There needs to be a real deterrent for FADs being discarded, I think. And if was a case of, 'OK, if we get one of your FADs washed up on our coastline, you have to pay something serious to this organization just for it being there' then that would be a decent deterrent." (INT-15)

Q26: "It [the PNA Arrangement] will gradually come up to a polluter pays model, I do believe that. Eventually. And that's fine, right? That's fair. That's economics. That's good citizenship." (INT-3)

Q27: "We would gather these things [dFADs] up and we would sell them at an auction. And people thought that they were like lights, so they would mount them to the post to make their house look nautical in their front yard or something." (INT-6)

Q28: "They [locals] don't like them [dFADs], they hate them. But they use them because they're here [and] because everything that you want to buy in [country] is so expensive. Some people did the compete light installation of their house with them... [one family] built their bathroom and made a wall only with bamboo coming from FADs." (INT-18)

*One interviewee also suggested a regulation regarding the spatial management of retrievals, i.e. prohibit vessels from fishing a dFAD for a specified time (e.g. one week) after it exits a MPA.
†This does not apply to dFAD buoys, which are unlikely to ever be fully biodegradable.
‡ The Parties to the Nauru Agreement are nine island nations whose EEZs collectively cover ~30 percent of the WCPFC Convention area. These countries manage tuna fishing in their waters through an effort-based program called the Vessel Day Scheme, requiring companies to pay USD 10,000 for a fishing day. The original PNA are: Federated States of Micronesia, Kiribati, Marshall Islands, Nauru, Palau, Papua New Guinea, Solomon Islands, and Tuvalu; Tokelau joined in 2012.

Table S7. Drivers of industry improvements to drifting fish aggregating device (dFAD) design and use as reported by industry interviewees (*n* =5). Acronyms: MSC= Marine Stewardship Council, ISSF = International Seafood Sustainability Foundation, NGO = Non-governmental organization.

Interviewee	Perceived driver	Quote(s)
INT-1	Voluntary best practice guidelines, industry collaboration	"We have 100% non-entangling [dFADs]. When we use netting, we don't use big netting that fish can get in. And also, when we use netting, the ISSF tells you to roll it like a sausage and then you tie it."<br><br>"I think this [multi-stakeholder collaboration] is the most important thing, because sometimes the proposals you get from people in the industry that are not directly related to the [onboard] operation are ideas that are good but are really hard to implement or impossible to implement [at-sea]… they [workshops with different stakeholders] are really good because [they include] fleet managers, the satellite buoy companies, scientists, authorities [and] because many of the things you want to implement have to be done through the RFMO, or the [country] government."
INT-2	Industry collaboration, market pressure, NGO engagement	"It [fishing] is a business, and we want to maintain the business. That is that is our main concern. Everybody has their own secret [for dFAD fishing] but the general consensus is that everybody is collaborating to try to improve practices."<br><br>"The tuna industry now is driven by markets. Really. We are pushing RFMOs because of the market. And who is behind retailers? NGOs. So, I would say, somehow, NGOs are the ones creating impact. I guarantee you, 100%, if NGOs request that retailers only source tuna that is guaranteed [from vessels] that are not fishing in

		a MPA, everybody will run behind them…All the effort we are doing is because of market access. Markets are our driving force [when] looking at FADs, looking at responsible FAD management."<br><br>"Another [area of] progress is the involvement of the authorities, the RFMOS. Regional Fisheries Management Organizations are taking seriously this matter with new conservation management measures that are linked to fishing licenses. If you are an infractor, you take the risk to lose your fishing license. That also this is a big progress. When I started [over a decade ago], we didn't have any CMMs on this. We have also a very important NGO, ISSF, the International Sustainability Foundation, pushing for that. And there is a better technology in tracking, like Global Fishing Watch is helping a lot with everyone. Authorities, private sector, NGOs, and even our buyers are checking us."
INT-3	Voluntary best practice guidelines, market pressure, MSC certification	"Some companies are more advanced than others or more responsible than others about trying to find workable solutions. And you'll always have early adopters and the stragglers…And some of that [recovery programs] is already happening voluntarily because of ISSF requirements. To be able to sell fish to a ISSF member, they've got requirements around participation in FAD recovery programs, bio[degradable] FADs, FAD tracking, submitting data to analyze…that's pushing industry along quicker than the RFMOs are."<br><br>"It's nice on some level that it [MSC conditions] aren't prescriptive because I think that produces more innovation because different companies will try different things perhaps. And then, you know, progress gets made in different ways."
INT-4	Industry collaboration	"A lot of what we do is kind of acting like a bridge between regulators, between NGOs, and between industry. So, we try to find kind of the

		common ground that everyone that everyone needs to push sustainability initiatives forward…no one on their own is going to be able to solve some of the issues that exist within the industry."
INT-5	Voluntary best practice guidelines, industry collaboration	"ISSF have the best practice and design for non-entangling FADs and they also have a biodegradable FAD [design]. And what I have seen, the industry is all good [with it] as long as it works close enough to what they are working with now because they don't want to spend thousands of dollars a day for fuel and the FAD not collect any tuna. ISSF knows that, and they do research with the tuna vessels to figure out which designs are better than others."

Table S8. Summary of MPA practitioner survey data on dFADs floating and stranding in specific Marine Protected Areas. Note: asterisk (*) denotes adjustment made to presence/absence survey question response based on contextual information provided by survey participant in open-ended comments and stranding frequency not provided to account for change (-). Timeframe refers to the number of years the respondent has been affiliated with the MPA; Stranding frequency from survey: 'n/a' = I have never seen one wash ashore in this MPA, 'Periodically'= 2-10 times per year. For MPAs with multiple survey responses, the perspectives of all respondents have been indicated where there is divergence.

Country	MPA	Surveys (n)	Timeframe (years)	dFAD stranding	dFAD stranding frequency	dFAD floating
Australia	Great Barrier Reef	1	5+	Unsure	n/a	Unsure
Belize	Glover's Reef	1	5+	No	n/a	No
Brazil	Monumento Natural Do Arquipélago De São Pedro E São Paulo	1	5+	No	n/a	No
Canada	Laurentian Channel Marine Protected Area	1	3	N/A	n/a	No
	St. Anns Bank Marine Protected Area	1	5	No	n/a	No
	Musquash Estuary Marine Protected Area	1	5+	No	n/a	No
Chile	Rapa Nui	1	5+	Yes (*)	-	No
	Nazca-Desventuradas	1	5+	No	n/a	No
Comoros	Parc National de Mohéli	1	5+	No (*)	-	No (*)
Costa Rica	Cocos Island National Park	1	5+	Yes	Periodically	Yes
Ecuador	Galápagos Islands	3	5+/5+/2	Yes	Monthly (*n*=1)/ Periodically (*n*=2)	Yes
Fed. States of Micronesia	Oroluk Marine Protected Area	1	5+	Yes	Unsure	Unsure
	Ahnd Atoll	1	< 1	Yes (*)	-	No
France (OST)	Mayotte	1	1	Yes	Periodically	Yes
	Archipel des Glorieuses	1	5+	Yes	Periodically	Yes
France	Estuaire De La Gironde Et Mer Des Pertuis	1	5+	No	n/a	No
Gabon	Reserve Aquatique du Grand Sud du Gabon	1	2	Yes	Periodically	Yes
Kenya	Kisite	1	5+	No	n/a	No
Kiribati	Southern Line Islands	1	5+	Yes	Periodically	Yes
Madagascar	Velondriake	1	5+	No	n/a	No
Monaco	Réserve sous-marine du Larvotto	1	1	No	n/a	No
Oman	Daymanyiat Islands Nature Reserve	1	5+	Unsure	n/a	Unsure
Palau	Palau National Marine Sanctuary	1	5+	Yes	Monthly	Yes
Philippines	Tubbataha Reefs Natural Park	1	5+	No	n/a	No

South Africa	iSimangaliso Marine Protected Area	1	5+	Yes	Weekly	Yes
	iSimangaliso Wetland Park	1	2	No	n/a	No
	Addo Elephant Marine Protected Area	1	5+	Yes	Periodically	No
	Table Mountain National Park Marine Protected Area	2	5+/4	Yes (*n*=1), No (*n*=1)	Periodically (*n*=1)/ n/a (*n*=1)	No
	Robben Island Marine Protected Area	1	5+	Yes	Periodically	No
	Namaqua National Park Marine Protected Area	1	5+	No	n/a	No
	West Coast National Park	1	5+	No	n/a	No
	De Hoop Marine Protected Area	1	3	No	n/a	No
	Amathole Marine Protected Area	1	5+	Unsure	n/a	Unsure
United Kingdom (OST)	Pitcairn Islands (Henderson Island)	1	5+	Yes	Periodically	Yes
Uruguay	Laguna Garzón	1	5+	No	n/a	No
	Laguna de Rocha	1	5+	No	n/a	No
United States of America	Papahanaumokuakea Marine National Monument	1	5+	No (*)	-	No
	Pacific Remote Islands Marine National Monument	1	5+	Yes	Monthly	Yes
	Monterey Bay National Marine Sanctuary	1	5+	No	n/a	No
	Greater Farallones National Marine Sanctuary	1	5+	No	n/a	No
	Palmyra Atoll National Wildlife Refuge	2	5+/5+	Yes	Monthly	Yes
	Wake Atoll National Wildlife Refuge	1	5+	Yes	Periodically	Yes
	Biscayne National Park	1	5+	Yes	Periodically	Yes
	Padre Island National Seashore	1	5+	Yes	Periodically	Yes
	Dana Point State Marine Conservation Area	1	3	Unsure	n/a	Unsure

Table S9. Summary of interviewee perspectives on dFAD impacts (observed and perceived) and solutions (existing and proposed). Note: interviewees from industry are indicated with italics. Since interviews were semi-structured, we asked all interviewees from each stakeholder group the same set of open-ended questions (see "Interview questions to MPA and industry stakeholders"). For interview content analysis, themes from each interviewee were identified and coded. As such, a '1' below indicates the presence of a theme noted by a specific interviewee (INT). Importantly, because interviewees were asked open-ended questions, a '0' *does not* mean the interviewee disagrees, nor that they did not answer a question, nor that no question on this topic was asked; a '0' means the interviewee did not mention that theme in response to a question (i.e., it was absent from the conversation).

			**Interviewee (INT)**																	
	Theme	Total	***1***	***2***	***3***	***4***	***5***	**6**	**7**	**8**	**9**	**10**	**11**	**12**	**13**	**14**	**15**	**16**	**17**	**18**
Impact	Unauthorized entry (intentional)	**2**	1	0	0	1	0	0	0	0	0	0	0	0	0	0	0	0	0	0
	Hazard to local vessels	**4**	0	0	0	0	0	0	1	0	1	0	0	0	0	0	1	0	0	1
	Invasive species transport	**2**	0	0	0	0	0	0	0	0	1	1	0	0	0	0	0	0	0	0
	Ecological trap	**4**	0	0	0	0	0	0	0	0	0	1	1	1	0	1	0	0	0	0
	Loss or abandonment: Unauthorized entry (accidental)	**18**	1	1	1	1	1	1	1	1	1	1	1	1	1	1	1	1	1	1
	Entanglement of at-risk species	**13**	0	0	1	0	0	0	1	1	1	1	1	1	1	1	1	1	1	1
	Pollution in protected area	**12**	1	0	1	0	0	1	1	1	1	0	1	0	1	1	1	0	1	1
	Plastic ingestion	**5**	0	0	0	0	0	0	0	1	1	0	0	0	1	0	1	0	1	0
	Habitat degradation	**16**	1	1	1	1	1	0	1	1	1	1	1	0	1	1	1	1	1	1
	Disruption of nesting behaviour	**4**	0	0	0	0	0	1	0	1	1	0	0	0	0	0	1	0	0	0
	Financial and logistical burden	**13**	0	0	0	0	0	1	1	1	1	1	1	1	1	1	1	1	1	1
	Burden on local waste management system	**7**	0	0	0	0	0	0	0	1	1	0	1	1	1	0	1	0	0	1
Solution	Limit interaction: Deployment limits	**6**	1	1	0	1	0	0	1	0	1	0	1	0	0	0	0	0	0	0
	Limit interaction: Spatial management of deployments	**10**	1	1	1	1	1	0	1	0	1	0	0	1	0	1	0	0	1	0
	Limit interaction: Limit de-activation and ensure at-sea retrieval	**7**	0	1	1	1	1	0	0	0	0	0	0	0	0	1	1	0	1	0
	Limit interaction: Other (i.e. shared dFADs, longer time-area closure)	**3**	1	1	0	1	0	0	0	0	0	0	0	0	0	0	0	0	0	0
	Minimize damage: Weight limit	**2**	0	0	0	1	1	0	0	0	0	0	0	0	0	0	0	0	0	0

	Minimize damage: Biodegradable materials	**10**	1	1	1	1	1	0	1	0	1	0	0	0	1	1	0	1	0	0
	Minimize damage: Non-entangling design	**8**	1	1	1	0	1	0	1	0	1	1	0	0	0	0	0	1	0	0
	Minimize damage: Other (i.e. re-usable dFADs, sinking sensors)	**3**	1	0	0	1	1	0	0	0	0	0	0	0	0	0	0	0	0	0
	Minimize damage: Nearshore retrieval programs	**9**	1	1	1	1	1	0	1	0	0	1	0	0	0	1	0	1	0	0
	Minimize damage: Beach cleanups	**10**	1	0	0	0	0	1	0	1	1	1	0	0	0	1	1	1	1	1
	Accountability: Buoy re-purposing	**11**	1	1	1	1	1	0	1	1	1	0	1	0	0	0	0	1	0	1
	Accountability: Standardized labelling and buoy traceability	**10**	1	1	0	0	0	1	1	1	1	1	0	0	0	1	0	0	1	1
	Accountability: Ownership rules and responsibilities	**10**	1	1	0	1	0	0	0	0	1	0	1	0	1	1	1	1	1	0
	Accountability: Polluter-pays model	**7**	1	0	1	0	1	0	0	0	0	0	0	0	1	1	1	1	0	0

Table S10. Adjustments made to account for perceived duplicates in MPA stranding analysis.

MPA name	WDPA ID retained	WDPA ID(s) removed
Moreton Bay	308684	95317
Great Barrier Reef	2628	2571
Phoenix Islands Protected Area	309888	555512002
Coiba	17831	902479 (Coiba National Park and its Special Zone of Marine Protection)
Golfo de Montijo	555705291	29809
Wake Atoll National Wildlife Refuge	555586816	555656182 (Wake Atoll), 400011 (Pacific Remote Islands Marine National Monument), 555672696 (Pacific Remote Islands Marine)
Palmyra Atoll National Wildlife Refuge	300046	555656077 (Palmyra Atoll), 555665485 (Palmyra Atoll National Wildlife Refu), 400011 (Pacific Remote Islands Marine National Monument), 555672696 (Pacific Remote Islands Marine)
Midway Atoll National Wildlife Refuge	18007	555656048 (Midway Atoll), 555512001 (Papahānaumokuākea), 555670040 (Papahanaumokuakea Marine), 220201 (Papahanaumokuakea Marine National Monument), 555672697 (Papahanaumokuakea Marine National Monument Particularly Sensitive Sea Area National Monument)
Rock Island Southern Lagoon	555547992	555645502 (Rock Island Southern Lagoon Management Area)
Marae Moana (Cook Islands Marine Park)	555624907	n/a (duplicate MPA names removed based on same WDPA ID: Marine Protected Area (Mangaia), Marine Protected Area (Rarotonga), Marine Protected Area (Aitutaki & Ngaputora), Marine Protected Area (Pukapuka/ Nassau), Marine Protected Area (Rakahanga/ Manihiki), Marine Protected Area (Penrhyn)
Qoliqoli Cokovata	555547828	555629253 (Macuata/Dreketi/Sasa/Mali Districts – different name, identical area)
Rakiraki District	555547769	555547844 (Navolau District – different name, identical area)

REFERENCES

1. C. M. Duarte, S. Agusti, E. Barbier, G. L. Britten, J. C. Castilla, J. P. Gattuso, R. W. Fulweiler, T. P. Hughes, N. Knowlton, C. E. Lovelock, H. K. Lotze, M. Predragovic, E. Poloczanska, C. Roberts, B. Worm, Rebuilding marine life. *Nature* **580**, 39–51 (2020).

2. UNEP-WCMC, IUCN, "Protected planet report 2024" (United Nations Environmental Programme; International Union for the Conservation of Nature, 2024); www.unep-wcmc.org.

3. Convention on biological diversity, "Kunming-Montreal Global Biodiversity Framework (CBD/COP/15/L.25)" (2022).

4. Présidence de la Polynésie française, Le modele Polynésien de préservation et de protection de l'océan salué par la communauté internationale [Presidency of French Polynesia: The Polynesian Model for Ocean Preservation and Protection Hailed by the International Community] (2025). https://www.presidence.pf/le-modele-polynesien-de-preservation-et-de-protection-de-locean-salue-par-la-communaute-internationale/.

5. Republic of Palau, Palau National Marine Sanctuary Act (Senate Bill 9-30, SD2, HD3) (2015).

6. Metabolic Consulting, *"On Track or Off Course? – Assessing Progress Toward the 30x30 Target for the Ocean"* (Bloomberg Ocean Fund, Campaign for Nature, the Marine Conservation Institute and Skytruth, 2024).

7. L. Claassens, C. M. Hernández, P. Biondi, J. Jaskiel, C. M. Karanassos, M. Mesengei, V. Nestor, E. I. Otto, H. Renguul, R. D. Rotjan, G. Sartori, L. Tatebe, Navigating large scale ocean science in a Pacific small island developing state. *Pac. Conserv. Biol.* **30**, 10.1071/pc24062 (2024).

8. C. M. Hernández, J. Witting, C. Willis, S. R. Thorrold, J. K. Llopiz, R. D. Rotjan, Evidence and patterns of tuna spawning inside a large no-take marine protected area. *Sci. Rep.* **9**, 10772 (2019).

9. J. Lynham, J. C. Villaseñor-Derbez, Evidence of spillover benefits from large-scale marine protected areas to purse seine fisheries. *Science* **386**, 1276–1281 (2024).

10. K. Boerder, A. Bryndum-Buchholz, B. Worm, Interactions of tuna fisheries with the Galápagos marine reserve. *Mar. Ecol. Prog. Ser.* **585**, 1–15 (2017).

11. S. Medoff, J. Lynham, J. Raynor, Spillover benefits from the world's largest fully protected MPA. *Science* **378**, 313–316 (2022).

12. T. Aqorau, J. Bell, J. N. Kittinger, Good governance for migratory species. *Science* **361**, 1208–1209 (2018).

13. K. Grorud-Colvert, J. Sullivan-Stack, C. Roberts, V. Constant, B. H. E. Costa, E. P. Pike, N. Kingston, D. Laffoley, E. Sala, J. Claudet, A. M. Friedlander, D. A. Gill, S. E. Lester, J. C. Day, E. J. Gonçalves, G. N. Ahmadia, M. Rand, A. Villagomez, N. C. Ban, G. G. Gurney, A. K. Spalding, N. J. Bennett, J. Briggs, L. E. Morgan, R. Moffitt, M. Deguignet, E. K. Pikitch, E. S. Darling, S. Jessen, S. O. Hameed, G. D. Carlo, P. Guidetti, J. M. Harris, J. Torre, Z. Kizilkaya, T. Agardy, P. Cury, N. J. Shah, K. Sack, L. Cao, M. Fernandez, J. Lubchenco, The MPA guide: A framework to achieve global goals for the ocean. *Science* **373**, eabf0861 (2021).

14. A. C. Widyatmoko, B. D. Hardesty, C. Wilcox, Detecting anchored fish aggregating devices (AFADs) and estimating use patterns from vessel tracking data in small-scale fisheries. *Sci. Rep.* **11**, 17909 (2021).

15. Oppian, *Halieutica, with an English Translation by A.W. Mair* (Harvard Univ. Press, 1928).

16. L. Dagorn, K. N. Holland, V. Restrepo, G. Moreno, Is it good or bad to fish with FADs? What are the real impacts of the use of drifting FADs on pelagic marine ecosystems? *Fish Fish.* **14**, 391–415 (2013).

17. L. Escalle, J. Mourot, P. Hamer, S. R. Hare, N. B. Phillip, G. M. Pilling, Towards non-entangling and biodegradable drifting fish aggregating devices – Baselines and transition in the world's largest tuna purse seine fishery. *Mar. Policy* **149**, 105500 (2023).

18. J. Lopez, G. Moreno, I. Sancristobal, J. Murua, Evolution and current state of the technology of echo-sounder buoys used by Spanish tropical tuna purse seiners in the Atlantic, Indian and Pacific Oceans. *Fish. Res.* **155**, 127–137 (2014).

19. M. Pons, D. Kaplan, G. Moreno, L. Escalle, F. Abascal, M. Hall, V. Restrepo, R. Hilborn, Benefits, concerns, and solutions of fishing for tunas with drifting fish aggregation devices. *Fish Fish.* **24**, 979–1002 (2023).

20. P. Guillotreau, F. Salladarré, M. Capello, A. Dupaix, L. Floc, A. Tidd, M. Tolotti, L. Dagorn, Is FAD fishing an economic trap? Effects of seasonal closures and other management measures on a purse-seine tuna fleet. *Fish Fish.* **25**, 151–167 (2024).

21. R. Gillett, M. Fong, "*Fisheries in the economies of Pacific Island countries and territories (Benefish Study 4)*" (Pacific Community, 2023).

22. D. Gershman, A. Nickson, M. O'Toole, "*Estimating The Use of FADs Around the World*" (2015); http://pewtrusts.org/~/media/assets/2015/11/global_fad_report.pdf?la=en.

23. L. Schiller, N. G. D'Costa, B. Worm, The global footprint of drifting fish aggregating devices. *Sci. Adv.* **11**, eads2902 (2025).

24. Marine Stewardship Council, The MSC theory of change: Commercial fishing, *msc.org* (2026). https://msc.org/en-au/what-we-are-doing/msc-theory-of-change/commercial-fishing.

25. A. Fonteneau, E. Chassot, N. Bodin, Global spatio-temporal patterns in tropical tuna purse seine fisheries on drifting fish aggregating devices (DFADs): Taking a historical perspective to inform current challenges. *Aquat. Living Resour.* **26**, 37–48 (2013).

26. J. D. Filmalter, M. Capello, J.-L. Deneubourg, P. D. Cowley, L. Dagorn, Looking behind the curtain: Quantifying massive shark mortality in fish aggregating devices. *Front. Ecol. Environ.* **11**, 291–296 (2013).

27. G. Gomez, S. Farquhar, H. Bell, E. Laschever, S. Hall, The IUU nature of FADs: Implications for tuna management and markets. *Coast. Manag.* **48**, 534–558 (2020).

28. D. J. Curnick, D. A. Feary, G. H. Cavalcante, Risks to large marine protected areas posed by drifting fish aggregation devices. *Conserv. Biol.* **35**, 1222–1232 (2021).

29. Q. Hanich, R. Davis, G. Holmes, E.-R. Amidjogbe, B. Campbell, Drifting fish aggregating devices (FADs): Deploying, soaking and setting—When is a FAD "fishing"? *Int. J. Mar. Coast. Law* **34**, 731–754 (2019).

30. Galapagos Conservation Trust, Galapagos National Park Directorate, "*Plastic Pollution Free Galapagos: 5 Years of Science to Solutions*" (2024).

31. Ascension Island MPA, What threatens the objectives of the MPA? (2023). https://ascensionmpa.ac/explore/what-threatens-the-mpa.

32. S. D. Balderson, L. E. C. Martin, "Environmental impacts and causation of 'beached' Drifting Fish Aggregating Devices around Seychelles Islands: A preliminary report on data collected by Island Conservation Society [IOTC–2015–WPEB11–39]" (Island Conservation Society, 2015).

33. A. J. Burt, J. Raguain, C. Sanchez, J. Brice, F. Fleischer-Dogley, R. Goldberg, S. Talma, M. Syposz, J. Mahony, J. Letori, C. Quanz, S. Ramkalawan, C. Francourt, I. Capricieuse, A. Antao, K. Belle, T. Zillhardt, J. Moumou, M. Roseline, J. Bonne, R. Marie, E. Constance, J. Suleman, L. A. Turnbull, The costs of removing the unsanctioned import of marine plastic litter to small island states. *Sci. Rep.* **10**, 14458 (2020).

34. A. Maufroy, E. Chassot, R. Joo, D. M. Kaplan, Large-scale examination of spatio-temporal patterns of drifting fish aggregating devices (dFADs) from tropical tuna fisheries of the Indian and Atlantic Oceans. *PLOS ONE* **10**, e0128023 (2015).

35. T. Imzilen, C. Lett, E. Chassot, A. Maufroy, M. Goujon, D. M. Kaplan, Recovery at sea of abandoned, lost or discarded drifting fish aggregating devices. *Nat. Sustainability* **5**, 593–602 (2022).

36. L. Escalle, P. Hamer, P. N. A. Office, "Spatial and temporal description of drifting FAD use in the WCPO derived from analyses of the FAD tracking programmes and observer data

[WCPFC-SC19-2023/EB-WP-05]" (Western and Central Pacific Fisheries Commission, 2023).

37. J. Lopez, M. H. Román, C. E. Lennert-Cody, M. N. Maunder, N. Vogel, L. M. Fuller, "Floating-object fishery indicators: A 2022 Report [DOCUMENT FAD-07-01]" (Inter-American Tropical Tuna Commission, 2022).

38. F. Paolo, D. Kroodsma, J. Raynor, T. Hochberg, P. Davis, J. Cleary, L. Marsaglia, S. Orofino, C. Thomas, P. Halpin, Satellite mapping reveals extensive industrial activity at sea. *Nature* **625**, 85–91 (2024).

39. D. A. Kroodsma, J. Mayorga, T. Hochberg, N. A. Miller, K. Boerder, F. Ferretti, A. Wilson, B. Bergman, T. D. White, B. A. Block, P. Woods, B. Sullivan, C. Costello, B. Worm, Tracking the global footprint of fisheries. *Science* **359**, 904–908 (2018).

40. Y. Baidai, L. Dagorn, M. J. Amande, D. Gaertner, M. Capello, "Aggregation process of tuna under drifting fish aggregating devices (dFADs) assessed through fisher's echosounder buoy in the Atlantic Ocean" (International Commission for the Conservation of Atlantic Tunas, 2020).

41. G. Moreno, L. Dagorn, G. Sancho, D. Itano, Fish behaviour from fishers' knowledge: The case study of tropical tuna around drifting fish aggregating devices (DFADs). *Can. J. Fish. Aquat. Sci.* **64**, 1517–1528 (2007).

42. E. Dyer, "Indian Ocean purse seine tuna fisheries using drifting fish aggregating devices: Compliance and environmental impacts," thesis, University of Exeter (2025).

43. E. Kimak, D. W. Kerstetter, T. D. Pitchford, Caribbean FAD tracking project dataset, Dataset (2021).

44. I. MacMillan, M. J. Attrill, T. Imzilen, C. Lett, S. Walmsley, C. Chu, D. M. Kaplan, Spatio-temporal variability in drifting fish aggregating device (dFAD) beaching events in the Seychelles archipelago. *ICES J. Mar. Sci.* **79**, 1687–1700 (2022).

45. J. Mourot, T. Thellier, J. Lopez, L. Fuller, M. Roman, D. David, D. Ochavillo, W. Smith, T. R. Nicholas, B. Tibatt, K. Stevens, L. Viapuna, B. Bigler, F. Prioul, M. Lercari, K. Pollock, K. Mesebeluu, S. A. Fook, M. Iakopo, J. Mesepitu, C. Halumwane, M. Batty, N. Doutreloux, B. Mugneret, J. M. Lynch, H. Tait, P. Hamer, L. Escalle, "Analyses of the regional database of stranded drifting fish aggregating devices (dFADs) in the Pacific Ocean: A 2024 update" (Inter-American Tropical Tuna Commission, 2025).

46. M. Clark, C. C. Mees, "Update on the catch and bycatch composition of illegal fishing in the British Indian Ocean Territory (BIOT) and a summary of abandoned and lost fishing gear [IOTC–2015–WPEB11–48]" (MRAG, 2015).

47. IPNLF, "Sustained systematic non-compliance of drifting fish aggregating devices (dFADs) with Resolution 19/02 'Procedures on a Fish Aggregating Devices (FADs) Management Plan' [IOTC-2023-CoC20-INF01_Rev1]" (International Pole and Line Foundation, 2023).

48. E. E. Kimak, "The distribution, composition, and management of drifting fish aggregating devices (Dfads) in the North Atlantic Ocean," thesis, Nova Southeastern University (2021).

49. P. Zarate, J. A. Juarez-Ceron, A. L. Sarti-Martinez, P. H. Dutton, "First results of the East Pacific green turtle, *Chelonia mydas*, nesting population assessment in the Galapagos Islands (NOAA Technical Memorandum NMFS-SEFSC-503)" in *22nd Annual Symposium on Sea Turtle Biology and Conservation* (2003).

50. M. I. Olendo, G. M. Okemwa, C. N. Munga, L. K. Mulupi, L. D. Mwasi, H. B. Mohamed, M. Sibanda, H. O. Ong'Anda, The value of long-term, community-based monitoring of marine turtle nesting: A study in the Lamu archipelago, Kenya. *Oryx* **53**, 71–80 (2019).

51. W. Dow, K. Eckert, M. Palmer, P. Kramer, "An atlas of sea turtle nesting habitat for the wider Caribbean region (WIDECAST Technical Report No. 6)" (The Wider Caribbean Sea Turtle Conservation Network and The Nature Conservancy, 2007); www.widecast.org.

52. L. Escalle, J. S. Phillips, J. Lopez, J. M. Lynch, H. Murua, S. J. Royer, Y. Swimmer, J. Murua, A. S. Gupta, V. Restrepo, G. Moreno, Simulating drifting fish aggregating device

trajectories to identify potential interactions with endangered sea turtles. *Conserv. Biol.* **38**, e14295 (2024).

53. E. S. Germanov, A. D. Marshall, I. G. Hendrawan, R. Admiraal, C. A. Rohner, J. Argeswara, R. Wulandari, M. R. Himawan, N. R. Loneragan, Microplastics on the menu: Plastics pollute Indonesian manta ray and whale shark feeding grounds. *Front. Mar. Sci.* **6**, 679 (2019).

54. J.-P. Hallier, D. Gaertner, Drifting fish aggregation devices could act as an ecological trap for tropical tuna species. *Mar. Ecol. Prog. Ser.* **353**, 255–264 (2008).

55. A. Dupaix, L. Dagorn, J. L. Deneubourg, M. Capello, Quantifying the impact of habitat modifications on species behavior and mortality: A case study of tropical tuna. *Ecol. Appl.* **34**, e3018 (2024).

56. T. Davies, D. Curnick, J. Barde, E. Chassot, "Potential environmental impacts caused by beaching events of drifting fish aggregating devices and identification of management solutions and uncertainties (IOTC-2017-WGFAD01-08 Rev_1)" (Indian Ocean Tuna Commission, 2017); http://iotc.org/documents/fad-management-plans.

57. C. Sieben, J. Gascoigne, S. des Clers, "Marine stewardship council (MSC) public certification report: Atlantic Ocean tropical tuna French purse seine fishery" [Control Union (UK) Limited, 2024].

58. L. Escalle, J. S. Phillips, M. Brownjohn, S. Brouwer, A. S. Gupta, E. V. Sebille, J. Hampton, G. Pilling, Environmental versus operational drivers of drifting FAD beaching in the Western and Central Pacific Ocean. *Sci. Rep.* **9**, 14005 (2019).

59. D. J. Amon, B. R. C. Kennedy, K. Cantwell, K. Suhre, D. Glickson, T. M. Shank, R. D. Rotjan, Deep-sea debris in the Central and Western Pacific Ocean. *Front. Mar. Sci.* **7**, 369 (2020).

60. P. Venter, P. Timm, G. Gunn, E. L. Roux, C. Serfontein, P. Smith, E. Smith, M. Bensch, D. Harding, P. Heemstra, Discovery of a viable population of coelacanths (Latimeria chalumnae Smith, 1939) at Sodwana Bay, South Africa. *S. Afr. J. Sci.* **96**, 567–568 (2000).

61. Shark Life Conservation, Responding to a floating FAD in iSimangaliso [video] (Instagram, 05 December 2025). https://instagram.com/p/DR4G-ssjKnw/.

62. J. D. Baker, T. C. Johanos, H. Ronco, B. L. Becker, J. Morioka, K. O'Brien, M. J. Donohue, Four decades of Hawaiian monk seal entanglement data reveal the benefits of plastic debris removal. *Science* **385**, 1491–1495 (2024).

63. Z. L. R. Botterell, F. Ribeiro, D. Alarcón-Ruales, E. Alfaro, J. Alfaro-Shigueto, N. Allan, N. Becerra, L. Braunholtz, S. Cardenas-Diaz, D. de Veer, G. Escobar-Sanchez, M. V. Gabela-Flores, B. J. Godley, I. Grønneberg, J. A. Howard, D. Honorato-Zimmer, J. S. Jones, C. Lewis, J. C. Mangel, M. Martin, J. P. M. Pérez, S. E. Nelms, C. Ortiz-Alvarez, A. Porter, M. Thiel, T. S. Galloway, Plastic pollution transcends marine protected area boundaries in the eastern tropical and south-eastern Pacific. *Mar. Pollut. Bull.* **201**, 116271 (2024).

64. Tri Marine, "Tri Marine: 2019 Sustainability Report" (2020).

65. TUNACONS, "Politica Pública de Gestión de Pantados para la CM 3.7 de ISSF" (2021).

66. J. Murua, G. Moreno, L. Dagorn, D. Itano, M. Hall, H. Murua, V. Restrepo, Improving sustainable practices in tuna purse seine fish aggregating device (FAD) fisheries worldwide through continued collaboration with fishers. *Front. Mar. Sci.* **10**, 1074340 (2023).

67. H. Murua, I. Zudaire, M. Tolotti, J. Murua, M. Capello, O. C. Basurko, I. Krug, M. Grande, I. Arregui, J. Uranga, J. M. Ferarios, P. Sabarros, J. Ruiz, Y. Baidai, M. L. Ramos, J. C. Báez, F. Abascal, H. Arrizabalaga, G. Moreno, L. Dagorn, J. Santiago, Lessons learnt from the first large-scale biodegradable FAD research experiment to mitigate drifting FADs impacts on the ecosystem. *Mar. Policy* **148**, 105394 (2023).

68. J. Murua, G. Moreno, M. Hall, L. Dagorn, D. Itano, V. Restrepo, "Towards global non-entangling fish aggregating device (FAD) use in tropical tuna purse seine fisheries through a participatory approach" (International Seafood Sustainability Foundation, 2017).

69. ISSF, "Non-entangling & biodegradable FADs guide" (International Seafood Sustainability Foundation, 2019).

70. L. Schiller, M. Bailey, Rapidly increasing eco-certification coverage transforming management of world's tuna fisheries. *Fish Fish.* **22**, 592–604 (2021).

71. V. Restrepo, L. Dagorn, G. Moreno, J. Murua, F. Forget, A. Justel-Rubio, "Report of the International Workshop on Mitigating Environmental Impacts of Tropical Tuna Purse Seine Fisheries" (International Seafood Sustainability Foundation, 2019).

72. J. Murua, N. Cuevas, M. Erauskin-Extramiana, L. Lopetegui-Eguren, I. Onandia, J. Ruiz, A. Salgado, G. Moreno, H. Murua, J. Santiago, "Have nonentangling DFAD reduced ghost fishing in the Indian Ocean?[IOTC-2025-WPEB21(AS)-33]" (Indian Ocean Tuna Commission, 2025).

73. IOTC, "Resolution 24/02 On Management of Drifting Fish Aggregating Devices (FADs) in the IOTC Area of Competence" (Indian Ocean Tuna Commission, 2024).

74. ICCAT, "Recommendation By ICCAT Replacing Recommendation 22–01 On A Multi-Annual Conservation And Management Programme For Tropical Tunas (Rec. 24–01)" (International Commission for the Conservation of Atlantic Tunas, 2024).

75. I. Zudaire, G. Moreno, J. Murua, P. Hamer, H. Murua, M. T. Tolotti, M. Roman, M. Hall, J. Lopez, M. Grande, G. Merino, L. Escalle, O. C. Basurko, M. Capello, L. Dagorn, M. L. Ramos, F. J. Abascal, J. C. Báez, P. J. Pascual-Alayón, S. Déniz, J. Santiago, Biodegradable drifting fish aggregating devices: Current status and future prospects. *Mar. Policy* **153**, 105659 (2023).

76. Republic of Guatemala, "Statement by the Republic of Guatemala regarding the implementation of the timeline for biodegradable FADs on the occasion of the 29th Meeting of the International Commission for the Conservation of Atlantic Tunas (ICCAT) [PLE_131/2025]" (International Commission for the Conservation of Atlantic Tunas, 2025).

77. G. Moreno, J. Salvador, I. Zudaire, J. Murua, J. L. Pelegrí, J. Uranga, H. Murua, M. Grande, J. Santiago, V. Restrepo, The Jelly-FAD: A paradigm shift in the design of biodegradable Fish Aggregating Devices. *Mar. Policy* **147**, 105352 (2023).

78. I. Zudaire, J. Santiago, M. Grande, H. Murua, P.-A. Adam, P. Nogués, T. Collier, M. Morgan, N. Khan, F. Baguette, J. Moron, I. Moniz, M. Herrera, "FAD watch: A collaborative initiative to minimize the impact of FADs in coastal ecosystems" (AZTI Technalia, 2018).

79. G. Moreno, G. Morán, P. Guerrero, "First International Workshop on FAD retrieval, Galápagos 2024 (8th Meeting of the IATTC Ad-Hoc Working Group on FADs)" (Inter-American Tropical Tuna Commission, 2024).

80. T. Imzilen, C. Lett, E. Chassot, D. M. Kaplan, Spatial management can significantly reduce dFAD beachings in Indian and Atlantic Ocean tropical tuna purse seine fisheries. *Biol. Conserv.* **254**, 108939 (2021).

81. V. Schatz, Assessing drifting fish aggregating device (dFAD) abandonment under International Marine Pollution Law. *Transnatl. Environ. Law* **13**, 243–263 (2024).

82. L. Escalle, S. Hare, P. Hamer, G. Pilling, "Pacific dFAD retrieval feasibility study [WCPFC-SC17-2021/EB-IP-17]" (Western and Central Pacific Fisheries Commission, 2021).

83. A. D. Sechi, R. Pietrasanta, F. Caruso, A. Bocconcelli, T. D. Lorenzi, A. Morello, M. Rosso, Anchored fish aggregating devices (FADs) are overlooked and growing source of pollution in Western Mediterranean Sea. *Mar. Pollut. Bull.* **219**, 118305 (2025).

84. A. S. Heile, E. Dyer, R. Bealey, M. Bailey, Drifting fish aggregating devices in the Indian ocean impacts, management, and policy implications. *NPJ Ocean Sustain.* **3**, 60 (2024).

85. H. Welch, T. Clavelle, T. D. White, M. A. Cimino, J. V. Osdel, T. Hochberg, D. Kroodsma, E. L. Hazen, Hot spots of unseen fishing vessels. *Sci. Adv.* **8**, eabq2109 (2022).

86. A. J. Nowakowski, S. W. J. Canty, N. J. Bennett, C. E. Cox, A. Valdivia, J. L. Deichmann, T. S. Akre, S. E. Bonilla-Anariba, S. Costedoat, M. McField, Co-benefits of marine protected areas for nature and people. *Nat. Sustainability* **6**, 1210–1218 (2023).

87. J. Rolfe, J. D. Valck, Values for protecting the Great Barrier Reef: A review and synthesis of studies over the past 35 years. *Mar. Pollut. Bull.* **169**, 112531 (2021).

88. G. Mentansan, M. Nauw, R. Awom, M. Ayorbaba, J. Lumi, D. Reeve, Sasi local wisdom as a cultural capital for sustainable tourism development in Raja Ampat Regency, West Papua. *I. J. Green Tourism Res. Appl.* **5**, 52–59 (2023).

89. Republic of the Seychelles, Nature Reserves and Conservancy (Seychelles Marine Spatial Plan) Regulations, 2025, *Nature Reserves and Conservancy Act*.

90. R. B. Johnson, A. J. Onwuegbuzie, Mixed methods research: A research paradigm whose time has come. *Educ. Res.* **33**, 14–26 (2004).

91. Pew, Mapping shark sanctuaries around the world [Data visualization] (2016), https://pew.org/en/research-and-analysis/data-visualizations/2016/shark-sanctuaries-around-the-world.

92. IUCN, The IUCN Red List of Threatened Species (version 2025-1). (2025). https://iucnredlist.org.

93. B. Worm, S. Orofino, E. S. Burns, N. G. D'costa, L. M. Feitosa, M. L. D. Palomares, L. Schiller, D. Bradley, Global shark fishing mortality still rising despite widespread regulatory change. *Science* **383**, 225–230 (2024).

94. M. Hennink, B. N. Kaiser, Sample sizes for saturation in qualitative research: A systematic review of empirical tests. *Soc. Sci. Med.* **292**, 114523 (2022).

95. O. A. Adeoye-Olatunde, N. L. Olenik, Research and scholarly methods: Semi-structured interviews. *J. Am. Coll. Clin. Pharm.* **4**, 1358–1367 (2021).

96. W. C. Adams, "Conducting semi-structured interviews" in *Handbook of Practical Program Evaluation: Fourth Edition* (Wiley Blackwell, 2015), pp. 492–505.

97. L. Escalle, D. Gaertner, P. Chavance, H. Murua, M. Simier, P. J. Pascual-Alayón, F. Ménard, J. Ruiz, F. Abascal, B. Mérigot, Catch and bycatch captured by tropical tuna purse-seine fishery in whale and whale shark associated sets: Comparison with free school and FAD sets. *Biodivers. Conserv.* **28**, 467–499 (2019).

98. Republic of Kiribati, *Environment Act 1999: Southern Line Islands Marine Protected Area Regulations 2020* (2020).

99. A. Maufroy, D. M. Kaplan, N. Bez, A. D. De Molina, H. Murua, L. Floch, E. Chassot, Massive increase in the use of drifting fish aggregating devices (dFADs) by tropical tuna purse seine fisheries in the Atlantic and Indian oceans. *ICES J. Mar. Sci.* **74**, 215–225 (2017).

100. L. Bergé, “Efficient estimation of maximum likelihood models with multiple fixed-effects: The R package FENmlm” (2018).

101. W. K. Newey, K. D. West, A. Simple, Positive semi-definite, heteroskedasticity and autocorrelation consistent covariance matrix. *Econometrica* **55**, 703–708 (1987).

102. L. Escalle, J. Mourot, T. Thellier, J. Lopez, L. Fuller, J. Wichman, S. J. Royer, L. Hood, B. Bigler, B. Jaugeon, T. R. Nicholas, K. Pollock, F. Prioul, A. Marks, M. Kutan, J. Jones, J. M. Lynch, H. Tait, P. Hamer, “Analyses of the regional database of stranded fish aggregating devices (dFADs) in the Pacific Ocean” (La Jolla, 2022).

103. IOTC, “Dynamics of drifting fish aggregating devices used in the large-scale purse seine fishery of the western Indian Ocean” (2023).

104. G. M. Velásquez, J. B. Medina, G. Unda, “FADs retrieval from the tuna purse seine fishery in the Galapagos Islands (2022 – May 2025)” (2025).

105. Parties to the Nauru Agreement, “A fourth arrangement implementing the Nauru Agreement Relating to Fish Aggregating Device (FAD) Tracking and FAD Buoy Registration” (2022).

106. Satlink, “2023 Sustainability Report” (2023).
